# Systematic evaluation of the isolated effect of tissue environment on the transcriptome using a single-cell RNA-seq atlas dataset

**DOI:** 10.1186/s12864-025-11614-w

**Published:** 2025-04-29

**Authors:** Daigo Okada, Jianshen Zhu, Kan Shota, Yuuki Nishimura, Kazuya Haraguchi

**Affiliations:** 1 Center for Genomic Medicine, Graduate School of Medicine, Kyoto University, Kyoto, 606 - 8507 Japan; 2 Discrete Mathematics Laboratory, Applied Mathematics and Physics Course, Graduate School of Informatics, Kyoto University, Kyoto, 606 - 8501 Japan; 3 Institute for Advanced Study, Gifu University, Gifu, 501 - 1193 Japan

**Keywords:** Single cell RNA-seq, Effect of tissue environment, Graph theory, Maximal biclique enumeration

## Abstract

**Background:**

Understanding cellular diversity throughout the body is essential for elucidating the complex functions of biological systems. Recently, large-scale single-cell omics datasets, known as omics atlases, have become available. These atlases encompass data from diverse tissues and cell-types, providing insights into the landscape of cell-type-specific gene expression. However, the isolated effect of the tissue environment has not been thoroughly investigated. Evaluating this isolated effect is challenging due to statistical confounding with cell-type effects, which arises from the highly limited subset of tissue-cell-type combinations that are biologically realized compared to the vast number of theoretical possibilities.

**Results:**

This study introduces a novel data analysis framework, named the Combinatorial Sub-dataset Extraction for Confounding Reduction (COSER), which addresses statistical confounding by using graph theory to enumerate appropriate sub-datasets. COSER enables the assessment of isolated effects of discrete variables in single cells. Applying COSER to the Tabula Muris Senis single-cell transcriptome atlas, we characterized the isolated impact of tissue environments. Our findings demonstrate that some genes are markedly affected by the tissue environment, particularly in modulating intercellular diversity in immune responses and their age-related changes.

**Conclusion:**

COSER provides a robust, general-purpose framework for evaluating the isolated effects of discrete variables from large-scale data mining. This approach reveals critical insights into the interplay between tissue environments and gene expression.

**Supplementary Information:**

The online version contains supplementary material available at 10.1186/s12864-025-11614-w.

## Background

Understanding the cell diversity across the entire body and the underlying molecular mechanisms is essential for elucidating the complex functions of biological systems. Cells that differentiate from a fertilized egg develop into a wide variety of cell-types, existing in appropriate proportions within each tissue. Single-cell omics enables the acquisition of detailed omics information at the individual cell level and serves as a powerful tool for investigating this cell diversity [20, 27]. Recently, large-scale single-cell omics datasets, referred to as omics atlases, have become available [5, 6, 12]. These datasets, which comprise data for a variety of tissues and cell-types, are valuable for comprehensive analyses of the effects of cellular features on gene expression using statistical models. However, while cell-type-related gene markers have been extensively studied in single-cell omics research [2, 3], the impacts of tissue environments have received limited attention.

Although omics atlases encompassing multiple tissues are publicly available, evaluating the isolated effect of the tissue environment on gene expression presents considerable challenges. A primary issue in such evaluations is statistical confounding with cell-type effects due to substantial missing and biases in the combinations of tissues and cell-types within the body. For example, while blood cells and fibroblasts are present in many tissues, certain cell-types are tissue-specific, such as hepatocytes in the liver or pancreatic cells in the pancreas. Furthermore, in tissues where cell collection is difficult, data may be limited to only a few cell-types. These factors contribute to statistical confounding between tissues and cell-types. Effectively addressing these challenges requires the development of novel data analysis techniques that account for the interrelationships among discrete variables.

Quantifying isolated tissue effects, though challenging, is fundamentally important for elucidating cellular diversity. When genes are expressed in the various cell-types that comprise a specific tissue but not in other tissues, it suggests that factors inherent to the tissue environment, such as cellular niches or secreted proteins, play a critical role in regulating gene expression. Conversely, when genes are expressed in multiple tissues within a given cell-type but not in different cell-types, internal cell-type effects, such as epigenetic status, are likely to be critical. Assessing the isolated effect of the tissue environment on gene expression is important for achieving a systems-level understanding of intercellular diversity within the transcriptome. Indeed, evidence from previous studies has highlighted the influence of the tissue environment on transcriptome data. For example, distinct age-related variations in gene expression have been observed in the same cell-types derived from different tissues [14]. Additionally, fibroblasts have been shown to exhibit heterogeneity among different tissues [19].

In this study, we introduce a novel data analysis framework, referred to here as Combinatorial Sub-dataset Extraction for Confounding Reduction (COSER). COSER enables the evaluation of the isolated effects of discrete variables in cells by overcoming statistical confounding by enumerating appropriate sub-datasets using graph theory. Application of this method to a large mouse scRNA-seq atlas dataset revealed the landscape of isolated tissue environment effects.

## Results

### Visualization of missing and bias in tissue and cell-type combinations in a single cell RNA-seq atlas

This study utilized the Tabula Muris Senis (TMS) dataset, a large-scale publicly available mouse single-cell RNA-seq dataset [5]. The TMS dataset serves as a valuable resource for aging research [4, 30], encompassing data from cells derived from 23 tissues collected from 30 mouse individuals across six age groups (1, 3, 18, 21, 24, and 30 months-old). The dataset includes a sex distribution of 19 males and 11 females. All cells in the dataset have been annotated with cell-types by the TMS project. We obtained log-transformed, pre-processed data from the TMS dataset, which comprises two subsets generated using distinct experimental methodologies: fluorescence-activated cell sorting (FACS) and droplet-based sequencing (Droplet). The FACS dataset contains expression data for 22,966 genes across 110,824 cells, while the Droplet dataset includes data for 20,138 genes across 245,389 cells.

**Fig. 1:**
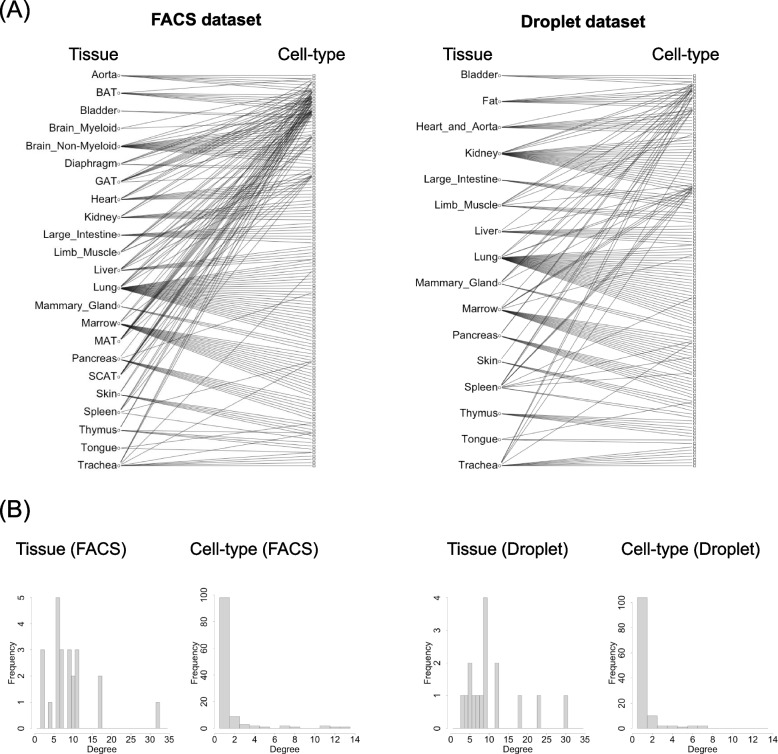
Bipartite graphs of tissue and cell-type combinations in the TMS dataset. **A** Visualization of the bipartite graphs in FACS and Droplet dataset. The missing and bias in combinations of tissues and cell-types are shown, highlighting the imbalance in their representation. All edges of these graphs are shown in Supplementary File 1. **B** The histograms of degrees of tissues and cell types in bipartite graphs of FACS and Droplet datasets

We visualized the missing and bias in the tissue and cell-type combinations in each dataset (Fig. 1A). A bipartite graph was constructed wherein the edges represent existing combinations between tissues and cell-types in the dataset. The graphs were constructed based on 207 observed combinations of 23 tissues and 120 cell types derived from the FACS dataset, representing only 7.5% of all theoretically possible combinations between tissues and cell types. Similarly, the Droplet dataset included 169 observed combinations of 20 tissues and 123 cell types, representing only 6.9% of all theoretically possible tissue-cell-type combinations. All edges of these graphs are shown in Supplementary File 1. The highly limited subset of tissue-cell-type combinations that are biologically realized compared to the vast number of theoretical possibilities would lead to statistical confounding, potentially hindering the accurate evaluation of individual effects.

The degree distribution of tissues and cell types in the bipartite graph presented in Fig. 1A is depicted in Fig. 1B. The average degree of tissues was 9 in the FACS dataset and 10.6 in the Droplet dataset. Conversely, the average degree of cell types was 1.7 in the FACS dataset and 1.4 in the Droplet dataset. While some cell types are present in multiple tissues, many are restricted to a single tissue or a limited number of tissues, indicating a bias in tissue-cell type associations. By restricting the analysis to cell types present in multiple tissues, one can achieve a more accurate and statistically robust quantification of the isolated effect of tissues.

Our research addresses the challenge of statistical confounding among discrete variables, including tissue and cell-type, when assessing the effects of the tissue environment on gene expression levels. Statistical confounding arises from the missing and bias in the combinations of these discrete variables, making it difficult to evaluate individual effects. Methods for preventing statistical confounding have been well researched in the field of experimental design in medical statistics, but there were no methods that could be used to solve problems of this kind in large-scale omics data mining. In theory, confounding can be resolved if all possible combinations of discrete variables were represented in the dataset. However, achieving this representation in real-world datasets is often impractical, necessitating the development of robust analytical methods to manage incomplete or biased combinations effectively.

### Brief description of the COSER framework

**Fig. 2:**
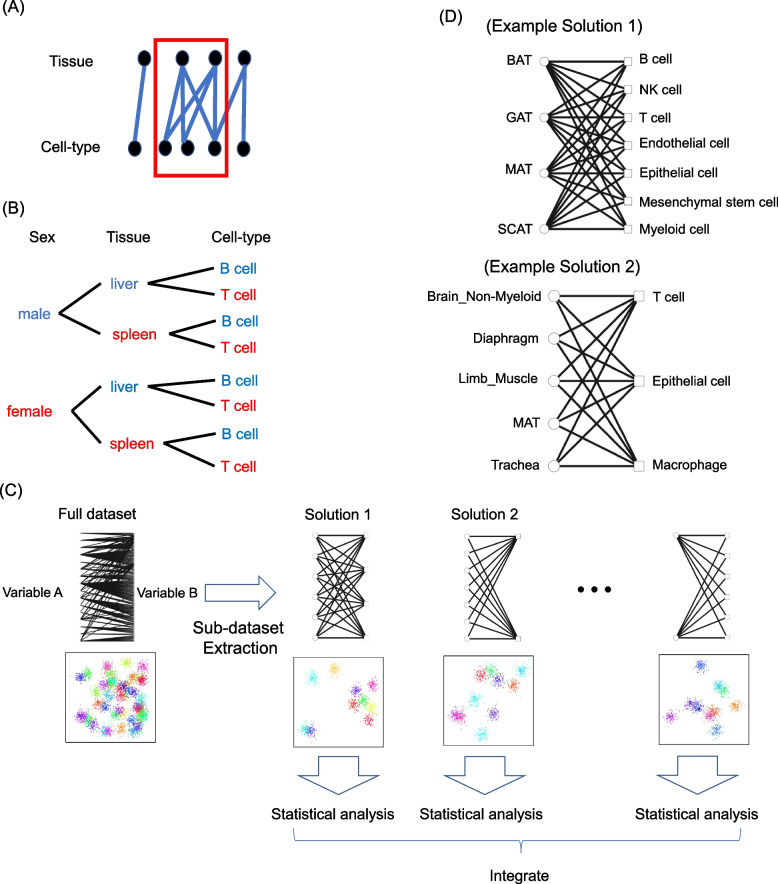
Extension of the maximal biclique enumeration problem to *k*-partite hypergraphs and the COSER framework. **A** Illustration of a maximal biclique. **B** An example of extending bicliques to *k*-partite hypergraphs, where the solution [[male, female], [liver, spleen], [T cell, B cell]] ensures the presence of all eight combinations shown in the tree diagram within the dataset. **C** Graphical overview of the COSER framework. These combinations of discrete variables in dataset are represented as a *k*-partite hypergraph. The subgraphs that contain all possible combinations are identified as solutions. For each solution, a sub-dataset is created that contains only the cells corresponding to the included combinations. Independent statistical analyses are conducted on sub-datasets, and their results are integrated to derive a consensus, ensuring robust insights into the variables’ isolated effects. **D** Examples of maximal bicliques in the bipartite graph of FACS dataset shown in Fig. 1

To address the issue of missing and bias in the combination of discrete variables such as tissue and cell-type, we propose a novel data analysis framework called Combinatorial Sub-dataset Extraction for Confounding Reduction (COSER). By constructing a bipartite graph where the edges represent combinations of variables (e.g., Fig. 1A), within this graph, bicliques, i.e., subgraphs containing all possible combinations of connected variables, are identified (Fig. 2A). These bicliques provide a robust foundation for statistical analysis by ensuring that variable combinations are comprehensively represented.

While identifying maximal bicliques in bipartite graphs is a well-established concept in graph theory, many biological datasets involve more than two discrete variables. To address this, we extended the maximal biclique enumeration problem to *k*-partite hypergraphs. For example, consider the combinations of three discrete variables of a single cell: sex, tissue, and cell-type. If all eight combinations exist in the dataset: male/liver/T-cell, male/liver/B-cell, male/spleen/T-cell, male/spleen/B-cell, female/liver/T-cell, female/liver/B-cell, female/spleen/T-cell, female/spleen/B-cell (Fig. 2B), [[male, female], [liver, spleen], [T cell, B cell]] forms an extended biclique in a *k*-partite hypergraph (k = 3). The developed algorithm accepts as input a table representing combinations of discrete variables that exist in the dataset. The algorithm detects the maximal solutions with ensuring that each solution includes at least two distinct values for all variables represented in the dataset. Further details of this extension and the algorithm developed to enumerate all maximal solutions in *k*-partite hypergraphs are provided in the Method section.

Figure 2C shows an overview of the COSER framework as applied to scRNA-seq. First, all of the combinations of discrete variables in the dataset are listed. These combinations of discrete variables are represented as a *k*-partite hypergraph. The developed algorithm then enumerates subgraphs that contain all possible combinations, identifying them as solutions. For each solution, a sub-dataset is created that contains only the cells corresponding to the included combinations. Statistical analyses are performed independently on each sub-dataset, allowing for unbiased statistical evaluation of the effects of individual variables on cellular phenotypes, such as gene expression levels. By integrating the results of these independent statistical analyses, a consensus conclusion is reached, providing robust insights into the isolated effects of the variables.

As an example implementation, we applied the COSER framework to the bipartite graph of the FACS dataset (Fig. 1A). As a result, 31 maximal solutions were identified (Supplementary File 2). These solutions represent suitable units of analysis for evaluating the impacts of the tissue environment or cell-type effects on cell phenotypes within the dataset. For example, Example 1 in Fig. 2D contains four adipose sub-tissues (i.e., brown adipose tissue (BAT), gonadal adipose tissue (GAT), mesenteric adipose tissue (MAT), and subcutaneous adipose tissue (SCAT)), and represents the solution with the largest number of edges. In contrast, Example 2 in Fig. 2D features another maximal solution and includes a more anatomically diverse set of tissues. These maximal solutions form bipartite cliques, which means that all of their combinations are included in the original dataset. Researchers can explore these enumerated maximal solutions to identify solutions that align with their research questions.

### Quantitative assessment of the isolated effect of the tissue environment

We used COSER to examine the isolated effect of the tissue environment on single-cell transcriptome profiles. This analysis specifically targeted cells from mice aged three months, focusing on the combinations of individuals, tissues, and cell-types. Only combinations containing more than ten cells were selected for downstream analysis. We applied COSER to a three-column table comprising individual, tissue, and cell-type combinations to enumerate the maximal solutions. We then quantified the tissue effect on gene expression levels using a generalized liner model (GLM) for each sub-dataset. In this model, gene expression values were treated as the objective variable, while individual, tissue, and cell-type were used as explanatory variables.

**Fig. 3:**
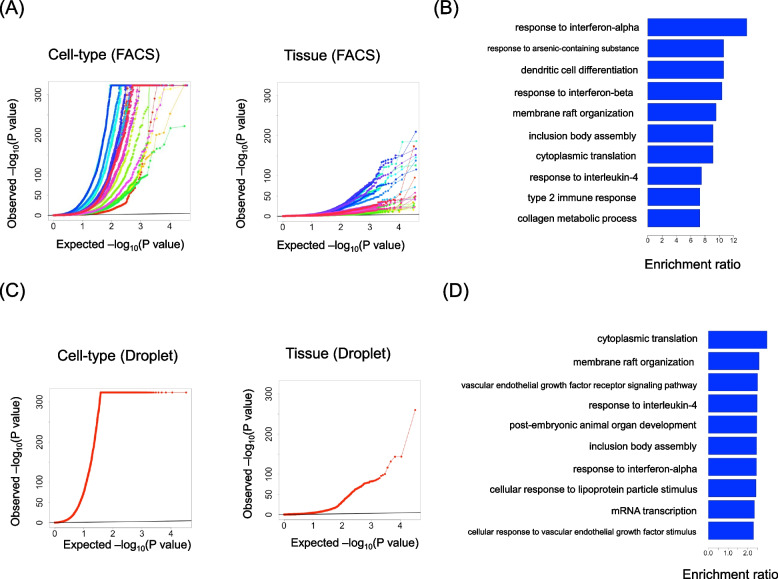
Isolated effects of the tissue environment observed throughout the body. **A** QQ plot showing P-values for the effect of tissue and cell-type in 24 sub-datasets from the FACS dataset. Each line corresponds to a sub-dataset. Zero P-values were replaced with the minimum non-zero P-value before log transformation. **B** GO terms with the top ten enrichment scores for genes affected by the tissue environment in the FACS dataset. **C** QQ plot showing P-values for the effect of the tissue and cell-type in one sub-dataset from the FACS dataset. **D** GO terms with the top ten enrichment scores for genes affected by the tissue environment in the Droplet dataset

In the FACS dataset, 24 maximal solutions for individual $\times$ tissue $\times$ cell-type combinations were identified (Table 1, Supplementary File 3). Figure 3A shows a QQ plot of the P-values obtained for the tissue and cell-type effects in each solution, demonstrating that not only cell-type but also tissue has an isolated effect on gene expression. The P-values for each gene in all sub-datasets are listed in Supplementary File 4. Genes exhibiting a significant tissue effect in more than 12 of the 24 sub-datasets (FDR < 0.05) were identified as tissue environment-susceptible genes. A total of 253 such genes were identified. Enrichment analysis of Gene Ontology (GO) Biological Processes for these genes revealed 135 significantly enriched GO terms within this gene set (FDR < 0.05) (Supplementary File 5). The ten GO terms with the highest enrichment scores are shown in Fig. 3B. The most significantly enriched biological process was GO:0035455 (response to interferon-alpha). Other biological processes related to immune responses, such as GO:0097028 (dendritic cell differentiation), GO:0035456 (response to interferon-beta), and GO:0070670 (response to interleukin- 4), were also highly represented. Additionally, a fundamental cellular function, GO:0002181 (cytoplasmic translation), also appeared among the enriched terms.

In the Droplet dataset, a single maximal solution comprising two tissues (limb muscle and mammary gland) was identified (Table 1). As with the FACS dataset, isolated tissue effects were observed. A statistically significant contribution from the tissue environment was detected in 3,581 genes (FDR < 0.05). The P-values for all genes are shown in Supplementary File 6. Figure 3C shows a QQ plot of the P-values obtained for tissue effects. Among these genes, 264 GO terms were significantly enriched (FDR < 0.05) (Supplementary File 7). The 10 GO terms with the highest enrichment scores are shown in Fig. 3D. Despite this result being based on a comparison between limb muscle and mammary gland, processes related to immune response and cytoplasmic translation were prominently represented, which is consistent with the FACS dataset.

**Table 1:** All maximal solutions for the combinations of individuals, tissues, and cell-types. BAT: brown adipose tissue, GAT: gonadal adipose tissue, MAT: mesenteric adipose tissue, SCAT: subcutaneous adipose tissue, LM: limb muscle, MG: mammary gland, MSC: mesenchymal stem cell/mesenchymal stem cell of adipose, MC: myeloid cell, BC: B cell, TC: T cell, SC: skeletal muscle satellite cell, MAC: macrophage, EC: endothelial cell

Name	No. of individuals	Tissue	Cell-type
FACS_solution1	7	GAT, SCAT	MSC, MC
FACS_solution2	5	MAT, SCAT	EC, MSC
FACS_solution3	5	BAT, SCAT	EC, MC
FACS_solution4	4	BAT, MAT	BC, EC
FACS_solution5	4	GAT, SCAT	EC, MSC, MC
FACS_solution6	4	Diaphragm, LM	MSC, SC
FACS_solution7	3	BAT, SCAT	BC, MC
FACS_solution8	3	GAT, MAT, SCAT	MSC, MC
FACS_solution9	3	BAT, GAT, SCAT	MSC, MC
FACS_solution10	3	GAT, MAT, SCAT	EC, MSC
FACS_solution11	3	BAT, GAT, SCAT	EC, MC
FACS_solution12	2	MAT, Spleen	BC, CD4+alpha-betaTC
FACS_solution13	2	BAT, MAT	BC, EC, MSC
FACS_solution14	2	BAT, SCAT	BC, MSC, MC
FACS_solution15	2	BAT, MAT	BC, EC, MC
FACS_solution16	2	BAT, SCAT	BC, EC, MC
FACS_solution17	2	BAT, Diaphragm	BC, EC
FACS_solution18	2	Diaphragm, LM	BC, MSC, SC
FACS_solution19	2	MAT, Trachea	EC, MAC
FACS_solution20	2	MAT, SCAT	EC, MSC, MC
FACS_solution21	2	BAT, GAT, SCAT	EC, MSC, MC
FACS_solution22	2	BAT, GAT, MAT, SCAT	EC, MSC
FACS_solution23	2	BAT, MAT, SCAT	EC, MC
FACS_solution24	2	Diaphragm, LM	EC, MSC, SC
Droplet_solution1	2	LM, MG	BC, TC, EC, MAC

By integrating the results of statistical analyses from the sub-datasets corresponding to each solution, it is possible to compare isolated tissue effects among tissues. To achieve this, a directed graph was constructed with tissues represented as nodes, based on the order of the coefficients of the tissue effects obtained from all 24 analyses in the FACS dataset. If this directed graph forms a directed acyclic graph (DAG), then the tissue effects are considered to have a partial ordering structure. Using the constructed DAG, a consistent order of tissue effects on gene expression was obtained. Among the identified tissue environment-susceptible genes, a DAG was successfully constructed for 54 genes (Supplementary File 8). Notably, four transcription factors were identified within these genes (*Fosb*, *Klf4*, *Tbx15*, *Wt1*).

**Fig. 4:**
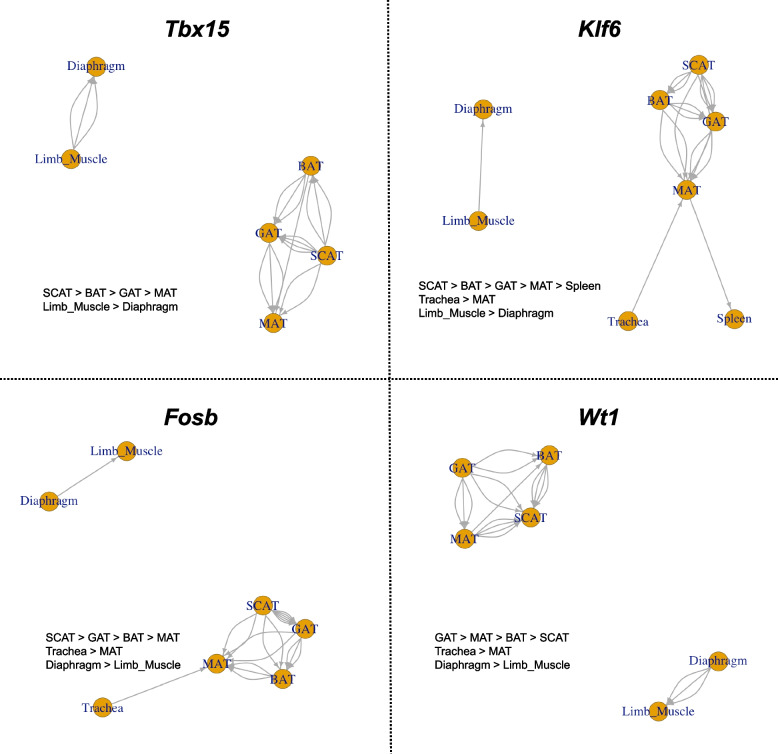
DAGs representing the relative magnitude of the isolated effect of tissue environments on the expression of four transcription factors (*Fosb*, *Klf4*, *Tbx15*, *Wt1*). The inequalities of the isolated tissue effects derived from the graphs are also included

Figure 4 shows the DAGs constructed for the four transcription factor genes. These DAGs provide valuable insights into the differences in gene expression among adipose sub-tissues. The effect of the tissue environment on *Tbx15* gene expression follows the order: SCAT > BAT > GAT > MAT. It has been reported that *Tbx15* is highly expressed in brown adipose tissue and essential for differentiating brown adipocytes [10]. The effect of the tissue environment on *Wt1* gene expression follows the order: GAT > MAT > BAT > SCAT. *Wt1* is known to be expressed in fat cell progenitors in visceral white adipose tissue but is absent in energy-storing subcutaneous white adipose tissue and brown adipose tissue (BAT) [15].Differences in gene expression among adipose sub-tissues have been previously reported for these genes. Our analysis suggests that these differences are driven more by the effects of the tissue microenvironment rather than by specific cell types. While there are no known differences in the expression of *Fosb*, *Klf4* between adipose sub-tissues, these genes are reported to be involved in adipogenesis and adipocyte differentiation [16, 18, 23]. Although further research is needed to understand how differences in the tissue environment affect gene expression, this analysis suggests that differences in the adipose tissue environment may affect the expression of these transcription factors, and this may control adipocyte differentiation and adipogenesis. By integrating the results of statistical model analyses for each sub-dataset into a graph where nodes represent tissues, this approach enables a systematic evaluation of the relative magnitude of tissue environment effects on gene expression.

### Detection of divergent aging patterns between different tissues

We investigated transcriptomic changes associated with aging in different tissue environments. Previous studies have demonstrated that age-related changes in gene expression can be specific to both tissue and cell-type [11, 26, 30]. In addition, data-mining analyses of the TMS dataset have identified genes that exhibit age-related increases in expression in specific sex-tissue-cell-type combinations while showing decreases in others (or vice versa) [21]. Understanding the underlying mechanisms driving these differences in age-related changes is crucial for advancing our knowledge of tissue-specific aging processes.

Using COSER, we investigated the occurrence of opposite aging effects within different tissue environments. For both the FACS and Droplet datasets, we focused on different combinations of sex, tissue, cell-type, and age. Donor age was defined as “Young” for three-month-old cells, “Old” for cells aged $\ge$ 18 months, and cells from one-month-old donors were excluded. Only combinations harboring more than 25 cells were selected for further analysis. When we applied COSER to this four-column table to identify sub-datasets, four maximal solutions were obtained using the FACS dataset while none were obtained using the Droplet dataset (Table 2).

**Table 2:** All maximal solutions for the combinations of tissue, cell-type, sex, and age

Name	Sex	Tissue	Cell-type	Age
FACS_solution1	Male, Female	BAT, SCAT	BC, EC, MC	Old, Young
FACS_solution2	Male, Female	GAT, SCAT	MSC, MC	Old, Young
FACS_solution3	Male, Female	MAT, SCAT	BC, EC, MSC	Old, Young
FACS_solution4	Male, Female	BAT, MAT, SCAT	BC, EC	Old, Young

We focused on two solutions from the FACS dataset, each containing at least three cell-types (FACS_solution1 and FACS_solution3). The sub-dataset corresponding to FACS_solution1 facilitates a comparative analysis of gene expression between GAT and SCAT. In contrast, the sub-dataset corresponding to FACS_solution3 facilitates the exploration of differences between MAT and SCAT.

**Fig. 5:**
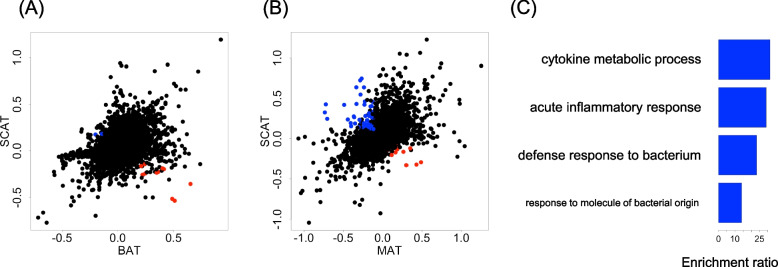
Comparison of age-related changes between different tissue environments. **A** Scatter plot of regression coefficient of the “Young” to gene expression level. Genes exhibiting the opposite effect of aging are colored red (X-axis is positive) or blue (Y-axis is positive). **B** Scatter plot of the “Young” regression coefficient versus the gene expression level (MAT vs. SCAT). **C** GO terms with the top ten enrichment scores for genes exhibiting the opposite effect of aging (BAT vs. SCAT)

We performed GLM analysis on cells from each tissue in a sub-dataset, and calculated the regression coefficients and P-values for the “Young” category across all genes in each tissue. We identified genes where the sign of the “Young” coefficient was opposite between tissues and both were statistically significant (FDR < 0.05). Scatter plots of the regression coefficients for the “Young” category in the two tissues are shown in Fig. 5A and B. A total of 14 genes in the BAT vs. SCAT comparison and 119 genes in the MAT vs. SCAT comparison exhibited different directions of significant age-related gene expression change. Detailed results, including regression coefficients for BAT vs. SCAT and MAT vs. SCAT, are shown in Supplementary File 9 and Supplementary File 10, respectively.

The findings showed that opposite aging effects are present even among different adipose sub-tissues. Specifically, four GO terms were significantly associated with genes exhibiting age-related expression changes in opposite directions between BAT and SCAT. These terms include GO:0042742 (defense response to bacterium), GO:0042107 (cytokine metabolic process), GO:0002237 (response to molecule of bacterial origin), and GO:0002526 (acute inflammatory response), as shown in Fig. 5C and Supplementary File 11. These results suggest that gene expression related to immunity and inflammation changes with age in opposing directions, depending on the tissue environment. Such findings highlight the importance of accounting for the influence of the tissue environment when studying age-related changes in immunity and inflammation. In the case of MAT vs. SCAT, no GO terms were significantly enriched.

## Discussion

In this study, we showed that a substantial number of genes are affected by the tissue environment. Specifically, various immune response pathways were associated with genes exhibiting differential expression affected by tissue environments. In addition, biological processes related to translation showed a strong association with the tissue environment. These findings highlight the importance of considering the tissue environment when analyzing cellular gene expression patterns. This investigation represents a pioneering effort to systematically evaluate the isolated effects of the tissue environment through data mining of a large-scale scRNA-seq atlas.

Aging analysis suggested that age-related changes in gene expression are influenced by the tissue environment. Specifically, genes exhibiting age-related changes were predominantly enriched in biological functions associated with immune responses. This decline in immune function with age, referred to as immunosenescence, is an important aspect of the aging process in individuals [1, 28]. While the tissue environment typically regulates the cellular states involved in the immune responses, aging may disrupt this regulatory process.

The biological significance of the phenomenon whereby some cell types are shared between different tissues, while many cell types are restricted to specific tissues, is one of the essential but unresolved questions. The definition of “cell type” itself is non-trivial, and it is not easy to give a clear answer to this question. However, one biological reason is that cells that carry out common and essential functions such as defense and immune surveillance, such as immune cells, can be cited. These cells can move throughout the body via the blood and lymphatic circulations and exist in many tissues. In this study, too, many of the main cell types observed across multiple tissues are immune system cells, suggesting a link to these universal functions.

There are several limitations to this study. First, the tissues were not evenly distributed in the detected solutions. For example, although the FACS dataset contains 23 tissues, only 8 were included in any of the 24 identified solutions. On the other hand, certain tissues, such as adipose tissue, were repeatedly detected in multiple solutions, allowing for detailed examination but preventing a comprehensive evaluation of all tissues in the body. In addition, when comparing the FACS dataset and the Droplet dataset from the same project, there was a significant difference in their applicability for evaluating tissue effects. While Droplet dataset contains more than two times of cells than FACS dataset, the solutions from Droplet dataset were more limited than FACS dataset. This discrepancy arises because the TMS dataset was not specifically designed to quantify tissue effects. Instead, the findings highlight the potential application of COSER to future research in experimental planning and statistical modeling for omics atlases in advance. Another limitation is that the main focus of this study is limited to mice aged 3 months, which are included in the TMS dataset. The TMS dataset is a prominent large-scale dataset that contains many mouse tissues throughout the body and cells of various ages. At present, such datasets are limited, but it is expected that more diverse datasets will appear in the future. By applying the developed method to datasets obtained under different species and conditions, it will be possible to analyze the effects of the tissue environment on cells in more detail and gain further insights.

In terms of future prospects, the key issue will be to elucidate the specific mechanisms by which the tissue environment affects gene expression. Changes in gene expression are affected not only by endogenous control via transcription factors and epigenetic regulatory mechanisms that cells inherently possess but also by signals received from surrounding cells and the extracellular matrix. However, this study focused on statistically quantifying the magnitude of the tissue effect and did not delve into the regulatory mechanisms at the molecular level. To elucidate such detailed mechanisms, it will be necessary to use experimental approaches to investigate interactions with the extracellular environment.

In this study, we developed novel data analysis framework, COSER, to enumerate suitable sub-datasets based on the combinations of discrete variables within a dataset. While selecting sub-datasets is an effective strategy for mitigating statistical confounding, manual extraction of suitable analysis units can be challenging. COSER addresses this issue by systematically selecting sub-datasets through the extension of the maximal bipartite clique enumeration problem to a *k*-partite hypergraph. From a future perspective, applying this approach to datasets from various omics layers has the potential to uncover the overall diversity and functional landscape at the cellular level, thereby contributing to advances in the life sciences. Although this study primarily focused on the effect of the tissue environment in a single scRNA-seq atlas dataset, the COSER framework could be applied to any dataset containing multiple discrete variables.

## Method

### Acquisition of data

The pre-processed scRNA-seq data of TMS dataset were obtained using the “TabulaMurisSenisData” package in R [25]. The list of mouse transcription factors was obtained from AnimalTFDB [24].

### Algorithm for sub-dataset extraction by extending the maximal biclique enumeration problem to *k*-partite hypergraphs

We model a table on experimental results (i.e., a dataset) by a hypergraph. A *hypergraph* $\textsf{H}=(V,\mathcal {E})$ is a pair of a set *V* of *vertices* and a set $\mathcal {E}$ of *hyperedges*, where $\mathcal {E}\subseteq 2^V$. For an integer $k \ge 2$, $\textsf{H}$ is *k-partite* if there is a partition $V = V_1 \cup V_2 \cup \cdots \cup V_k$ such that, for every hyperedge $H \in \mathcal {E}$, $|H \cap V_i| = 1$, $i = 1, 2, \dots , k$ holds. A conventional bipartite graph is a 2-partite hypergraph in this terminology. For a subset $S\subseteq V$, we denote $S_i:=S\cap V_i$, $i=1,2,\dots ,k$. For a subset *S* such that all of $S_1,S_2,\dots ,S_k$ are non-empty and $q\in \{1,2,\dots ,k\}$, we define $\Pi _q(S)\triangleq \{\{v_1,v_2,\dots ,v_q\}\mid v_i\in S_i,\ i=1,2,\dots ,q\}$. In other words, $\Pi _q(S)$ is the set of all combinations of vertices that are taken from $S_1,S_2,\dots ,S_q$ one by one, respectively.

Let $\textsf{H}=(V,\mathcal {E})$ be a *k*-partite hypergraph and $\theta _1,\theta _2,\dots ,\theta _k$ be positive integers. We call a tuple $(\textsf{H};\theta _1,\theta _2,\dots ,\theta _k)$ an *instance*. We call $S\subseteq V$ a *solution to the instance* if

- $|S_i|\ge \theta _i$ holds for all $i=1,2,\dots ,k$; and
- $$\Pi _k(S)\subseteq \mathcal {E}$$ holds.

A solution *S* is *maximal* if there is no solution that is a proper superset of *S*. It is easy to see that, when $k=2$ and $\theta _1=\theta _2=1$, a solution corresponds to a biclique in a bipartite graph. Parameter $\theta _i$, $i=1,2,\dots ,k$ is determined by users and represents a threshold on the number of entries in $V_i$ that is regarded as significant.

In our context, a table like Fig. 5A can be represented by a *k*-partite hypergraph such that each vertex corresponds to an entry in the table (e.g., “Female”, “BAT”, “BC”, “Old”); each subset $V_i$, $i=1,2,\dots ,k$ in the partition corresponds to a column of the table (e.g., “Sex”, “Tissue”, “Cell-type”, “Age”); and each hyperedge $H\in \mathcal {E}$ corresponds to a row of the table. A solution *S* to the instance $(\textsf{H};\theta _1,\theta _2,\dots ,\theta _k)$ corresponds to a set of rows in the dataset such that all possible $|S_1|\times |S_2|\times \dots \times |S_k|$ combinations of entries appear, where $|S_i|\ge \theta _i$, $i=1,2,\dots ,k$ holds. In our experiments, we will construct $\textsf{H}$ from a given dataset as above, and set $\theta _1=\theta _2=\cdots =\theta _k:=2$.

We consider the problem of enumerating all maximal solutions. Let $n:=|V|$, $m:=|\mathcal {E}|$, and *N* denote the number of all maximal solutions. It is inevitable that the enumeration task takes $\Omega (N)$ time, and *N* can be up to an exponential number with respect to *n* and *m*. For example, when $k=2$, there exist $2^{\min \{|V_1|,|V_2|\}}$ maximal solutions in crown graphs. A natural question is to ask whether or not we can enumerate all maximal solutions in polynomial time with respect to *n*, *m* and *N* (i.e., output-polynomial time [13]). Unfortunately, it is hopeless to obtain such an algorithm since it is NP-complete to decide whether there exists a solution even for the case of $k=2$ and $\theta _1=\theta _2$ [9]. This indicates that there is no polynomial-time algorithm to find a maximal solution unless P$=$NP.

Based on the hardness of the problem, we decide to focus on developing an algorithm that completes the enumeration task for our datasets in practical time while the time complexity bound is trivial $O^*(2^n)$, where $O^*(\cdot )$ ignores polynomial factors. For space complexity, the algorithm uses $O^*(2^n)$ space to store all candidates of maximal solutions. However, the algorithm is efficient enough for our datasets.

Let us introduce notations for preparation. For a vertex $v\in V$, we denote by $\mathcal {E}(v)$ the set of all hyperedges in $\mathcal {E}$ that contain *v*. We define $\hat{\mathcal {E}}(v)$ to be the family of all vertex subsets that are obtained by deleting *v* from a hyperedge in $\mathcal {E}(v)$; i.e., $\hat{\mathcal {E}}(v)\triangleq \{H\setminus \{v\}\mid H\in \mathcal {E}(v)\}$. For $i\in \{1,2,\dots ,k\}$, let $U\subseteq V_i$. We define $\hat{\mathcal {E}}(U):=\bigcap _{v\in U}\hat{\mathcal {E}}(v)$. For any subset $F\in \hat{\mathcal {E}}(U)$ and vertex $v\in U$, the union $F\cup \{v\}$ is a hyperedge in $\mathcal {E}$. For a *k*-partite hypergraph $\textsf{H}$, let us define an auxiliary bipartite graph $\textsf{B}_\textsf{H}=(V_k\cup W_{\textsf{H}},E_\textsf{H})$ such that $W_{\textsf{H}}:=\bigcup _{v\in V_k}\hat{\mathcal {E}}(v)$ and $E_{\textsf{H}}:=\{(v,\hat{H})\mid v\in V_k,\hat{H}\in W_{\textsf{H}}, \{v\}\cup \hat{H}\in \mathcal {E}\}$. Note that each node $\hat{H}\in W_{\textsf{H}}$ is a subset of $V_1\cup V_2\cup \dots \cup V_{k-1}$.

In order to avoid redundant search, we restrict the search space by using two necessary conditions that should be satisfied by any maximal solution. Let $\mathcal {I}=(\textsf{H}=(V,\mathcal {E});\theta _1,\theta _2,\dots ,\theta _k)$. First, if *S* is a maximal solution to $\mathcal {I}$, then there is a maximal solution *B* to the auxiliary instance $\mathcal {J}=(\textsf{B}_\textsf{H};\theta _k,1)$ such that $S_k=B\cap V_k$. This indicates that $S_k$ of maximal solutions *S* to $\mathcal {I}$ are within maximal solutions to $\mathcal {J}$, where the latter solutions can be efficiently computed by existing biclique enumeration algorithms. Second, if *S* is a maximal solution to $\mathcal {I}$, then $S\setminus S_k=S_1\cup S_2\cup \dots \cup S_{k-1}$ must be a maximal solution in the reduced instance $\mathcal {I}'=(\textsf{H}';\theta _1,\theta _2,\dots ,\theta _{k-1})$, where $\textsf{H}':=(V_1\cup V_2\cup \dots \cup V_{k-1},\hat{\mathcal {E}}(S_k))$. Using this property, we generate the candidates of $S_k,S_{k-1},\dots ,S_1$ recursively and maintain $S_k\cup S_{k-1}\cup \dots \cup S_1$ as a candidate of a maximal solution to $\mathcal {I}$. After generating all candidates, we output those which are inclusion-wise maximal.

The two necessary conditions are summarized as Lemmas 1 and 2 as follows.

#### Lemma 1

For an instance $\mathcal {I}=(\textsf{H}=(V,\mathcal {E});\theta _1,\theta _2,\dots ,\theta _k)$, if $S\subseteq V$ is a maximal solution to $\mathcal {I}$, then $S_k\cup \hat{\mathcal {E}}(S_k)$ is a maximal solution to $\mathcal {J}=(\textsf{B}_\textsf{H};\theta _k,1)$.

#### Proof

We see that $\hat{\mathcal {E}}(S_k)=(\bigcap _{v\in S_k}\hat{\mathcal {E}}(v))\subseteq (\bigcup _{v\in V_k}\hat{\mathcal {E}}(v))=W_{\textsf{H}}$. For every $u\in S_k$ and $\hat{H}\in \hat{\mathcal {E}}(S_k)$, it holds that $(u,\hat{H})\in E_\textsf{H}$ since $\hat{H}\in \hat{\mathcal {E}}(S_k)=\bigcap _{v\in S_k}\hat{\mathcal {E}}(v)\subseteq \hat{\mathcal {E}}(u)$, indicating that $\{u\}\cup \hat{H}\in \mathcal {E}$. Then $S_k\cup \hat{\mathcal {E}}(S_k)$ is a solution to $\mathcal {J}$, where the maximality is obvious. $\square$

#### Lemma 2

Suppose that we are given an instance $\mathcal {I}=(\textsf{H}=(V,\mathcal {E});\theta _1,\theta _2,\dots ,\theta _k)$. Let *S* be a solution to $\mathcal {I}$ and $\textsf{H}':=(V_1\cup V_2\cup \dots \cup V_{k-1},\hat{\mathcal {E}}(S_k))$. (i) The hypergraph $\textsf{H}'$ is $(k-1)$-partite. (ii) If *S* is a maximal solution to $\mathcal {I}$, then $S\setminus S_k$ is a maximal solution to $\mathcal {I}':=(\textsf{H}'; \theta _1,\theta _2,\dots ,\theta _{k-1})$.

#### Proof

(i) We see that $\hat{\mathcal {E}}(S_k)=\bigcap _{v\in S_k}\hat{\mathcal {E}}(v)$ by the definition, where each $\hat{H}\in \hat{\mathcal {E}}(v)$ satisfies $|\hat{H}\cap V_j|=1$ for $j=1,2,\dots ,k-1$ since $\textsf{H}$ is *k*-partite. This shows that $\textsf{H}'$ is $(k-1)$-partite.

(ii) By (i), $\textsf{H}'$ is $(k-1)$-partite. Obviously $|S_i|\ge \theta _i$ holds for $i=1,2,\dots ,k-1$. Let $H:=\{v_1,v_2,\dots ,v_{k-1}\}\in \Pi _{k-1}(S)$. For every $v_k\in S_k$, we have $H\cup \{v_k\}\in \mathcal {E}(v_k)\subseteq \mathcal {E}$ since *S* is a solution to $\mathcal {I}$. Then $H\in \bigcap _{v_k\in S_k}\hat{\mathcal {E}}(v_k)=\hat{\mathcal {E}}(S_k)$ holds, where we see that $S\setminus S_k$ is a solution to $\mathcal {I}'$. If $S\setminus S_k$ is not maximal, then there would be a solution $S^+$ to $\mathcal {I}'$ such that $S^+\supsetneq S\setminus S_k$. For $u\in S^+\setminus (S\setminus S_k)$, it is easy to see that $S\cup \{u\}$ is a solution to $\mathcal {I}$, contradicting the maximality of *S*. $\square$

Now we are ready to present an algorithm to enumerate all maximal solutions. The algorithm is summarized as EnumMaxSol in Algorithm 1.

**Figure:**
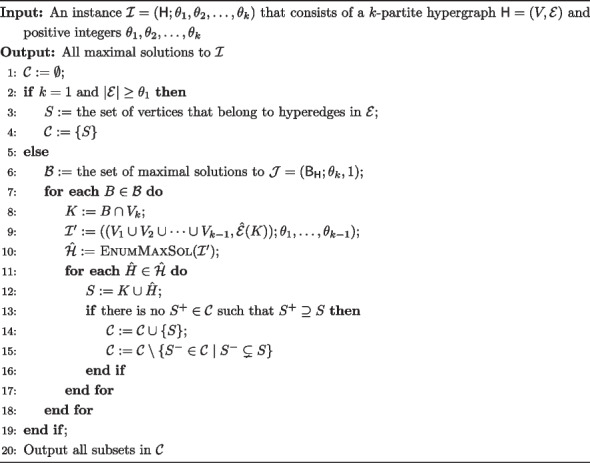
**Algorithm 1** An algorithm EnumMaxSol $(\mathcal {I})$ to output all maximal solutions to a given instance $\mathcal {I}$

#### Theorem 1

For an instance $\mathcal {I}$, Algorithm 1 enumerates all maximal solutions.

#### Proof

We show the correctness of the algorithm by induction on *k*. If $k=1$, then every hyperedge in $\mathcal {E}$ consists of precisely one vertex in *V*. The unique maximal solution is the set *S* of vertices that are contained in hyperedges and hence $|S|=|\mathcal {E}|$ holds. We see that Algorithm 1 outputs *S* if $k=1$ and $|S|\ge \theta _1$.

Suppose $k>1$ and that Algorithm 1 works correctly for $k-1$. We show that every subset *S* in $\mathcal {C}$ is a solution (which may not be maximal) at any time in the execution of the algorithm; *S* is constructed by taking the union $S=K\cup \hat{H}$ in line 12. The set *K* is a subset of $V_k$ (line 8) and also a subset of a maximal solution *B* to $\mathcal {J}$ that is generated in line 6. Then $|K|\ge \theta _k$ holds. Also, $\hat{H}$ is a solution to $\mathcal {I}'$ in line 9 that satisfies $|\hat{H}\cap V_i|\ge \theta _i$, $i=1,2,\dots ,k-1$. One readily sees that $\Pi _k(S)\subseteq \mathcal {E}$ holds and hence *S* is a solution.

We show that any maximal solution *S* to $\mathcal {I}$ belongs to $\mathcal {C}$ when the algorithm terminates. By Lemma 1, there is a maximal solution *B* to $\mathcal {J}$ such that $S_k=B\cap V_k$, where *B* is exactly generated in line 6. Let us denote $\hat{H}:=S\setminus S_k$. By Lemma 2(ii), $\hat{H}$ is a maximal solution to $\mathcal {I}'$, and by the induction assumption, the recursive call in line 10 exactly generates $\hat{H}$. Then $S=S_k\cup \hat{H}$ is added to $\mathcal {C}$ in line 14 since it is inclusion-wise maximal and hence no maximal solution $S^+\supsetneq S$ to $\mathcal {I}$ exists. Once *S* is added to $\mathcal {C}$, it is not excluded from $\mathcal {C}$ in line 15.

A non-maximal solution cannot belong to $\mathcal {C}$ at the end of the algorithm since all maximal solutions are contained in $\mathcal {C}$ and any non-maximal solution has not been included to $\mathcal {C}$ by line 13 or has been excluded from $\mathcal {C}$ in line 15. Then $\mathcal {C}$ is the set of all maximal solutions when the algorithm terminates. $\square$

Let us make remarks on our Python implementation of the algorithm. In line 6, we enumerate bicliques in $\textsf{B}_\textsf{H}$ by the algorithm of [29]. We use data structure set to realize the family $\mathcal {C}$ of candidate solutions, which is essentially a hash table.

### Generalized linear model analysis

A GLM was used to evaluate the contribution of discrete variables to each gene’s expression level. Discrete variables, such as sex, tissue, and cell-type, were used as explanatory variables. Our model can be expressed as follows.

$$\begin{aligned} y_i = b_{sex} \times sex_i + b_{tissue} \times tissue + b_{celltype} \times celltype + error \end{aligned}$$

$y_i$ is the gene expression level of the *i*-th cell, and $sex_i$, $tissue_i$, and $celltype_i$ are the attributes of the *i*-th cell. the error term is the random error under a gaussian distribution. $b_{sex}$, $b_{tissue}$, $b_{celltype}$ are the partial regression coefficient of each discrete variable and represents the isolated effect of that variable. The partial regression coefficients and *P*-values for the discrete variables were calculated using the “glm” function in R with default settings. The *P*-values for each discrete variable were calculated using Type II ANOVA, implemented with the “Anova” function from the R package “car” (version 3.1.3) [8].

### Gene Ontology analysis

Gene Ontology analysis was performed for the biological interpretation of the results derived from the omics analysis performed using the proposed method. Gene Ontology analysis was performed using the “WebGestaltR” package (version 0.4.6) in R [17]. The “WebGestaltR” function within this package was used for the analysis, and Over-Representation Analysis (ORA) was specified as the method. The analysis focused on the GO category “Biological Process”, using the database setting “geneontology_Biological_Process_noRedundant”.

### Data visualization

The bipartite graph was visualized using the “igraph” (version 1.6.0) and “multigraph” (version 0.99) packages in R [7, 22].

### Software availability and requirements

 

- Project name: k-partite-hypergraph
- Project home page: https://github.com/ku-dml/k-partite-hypergraph
- Operating system(s): Platform independent
- Programming language: Python
- Other requirements: Python 3.9 or higher, pandas $\ge$ 1.4.2
- License: MIT License
- Any restrictions to use by non-academics: License needed

## Supplementary information

Supplementary File 1. All combinations of tissues and cell-types in the FACS and Droplet datasets.

Supplementary File 2. All maximal solutions identified from the combinations of tissues and cell-types in the FACS dataset.

Supplementary File 3. All maximal solutions identified from the combinations of individuals, tissues, and cell-types in the FACS dataset.

Supplementary File 4. *P*-values for tissue and cell-type effects for all genes from the statistical analysis of 24 sub-datasets derived from the FACS dataset.

Supplementary File 5. Complete results of the Gene Ontology (GO) analysis for genes affected by the tissue environment in the FACS dataset. The table was generated using the “WebGestaltR” package in R.

Supplementary File 6. P-values for tissue and cell-type effects for all genes from the statistical analysis of one sub-dataset derived from the Droplet dataset.

Supplementary File 7. Complete results of the Gene Ontology (GO) analysis for genes significantly affected by the tissue environment in the sub-dataset of the Droplet data (limb muscle vs. mammary gland). The table was generated using the “WebGestaltR” package in R.

Supplementary File 8. Directed acyclic graphs (DAGs) constructed for all 54 genes identified in this analysis.

Supplementary File 9. Complete results of the regression analysis from the aging study (BAT vs. SCAT). The table shows regression coefficients, *P*-values, and false discovery rate (FDR) values for “Young” category in each tissue.

Supplementary File 10. Complete results of the regression analysis from the aging study (MAT vs. SCAT). The table shows regression coefficients, *P*-values, and false discovery rate (FDR) values for the “Young” category in each tissue.

Supplementary File 11. Complete results of the Gene Ontology (GO) analysis for genes with expression changes in opposite directions between BAT and SCAT. The table was generated using the “WebGestaltR” package in R.

## Data Availability

The R code used for the simulation and data analysis is available at https://github.com/DaigoOkada/scRNAseq-biclique. The developed software is available at https://github.com/ku-dml/k-partite-hypergraph.
