# Supplementary material for: Systematic evaluation of the isolated effect of tissue environment on the transcriptome using a single-cell RNA-seq atlas dataset
Source: BMC Genomics. 2025 Apr 29;26:416. doi: 10.1186/s12864-025-11614-w (PMC12039055; doi:10.1186/s12864-025-11614-w)
Supplement: Supplementary file 8 — Directed acyclic graphs (DAGs) constructed for all 54 genes identified in this analysis. [file 12864_2025_11614_MOESM8_ESM.pdf]

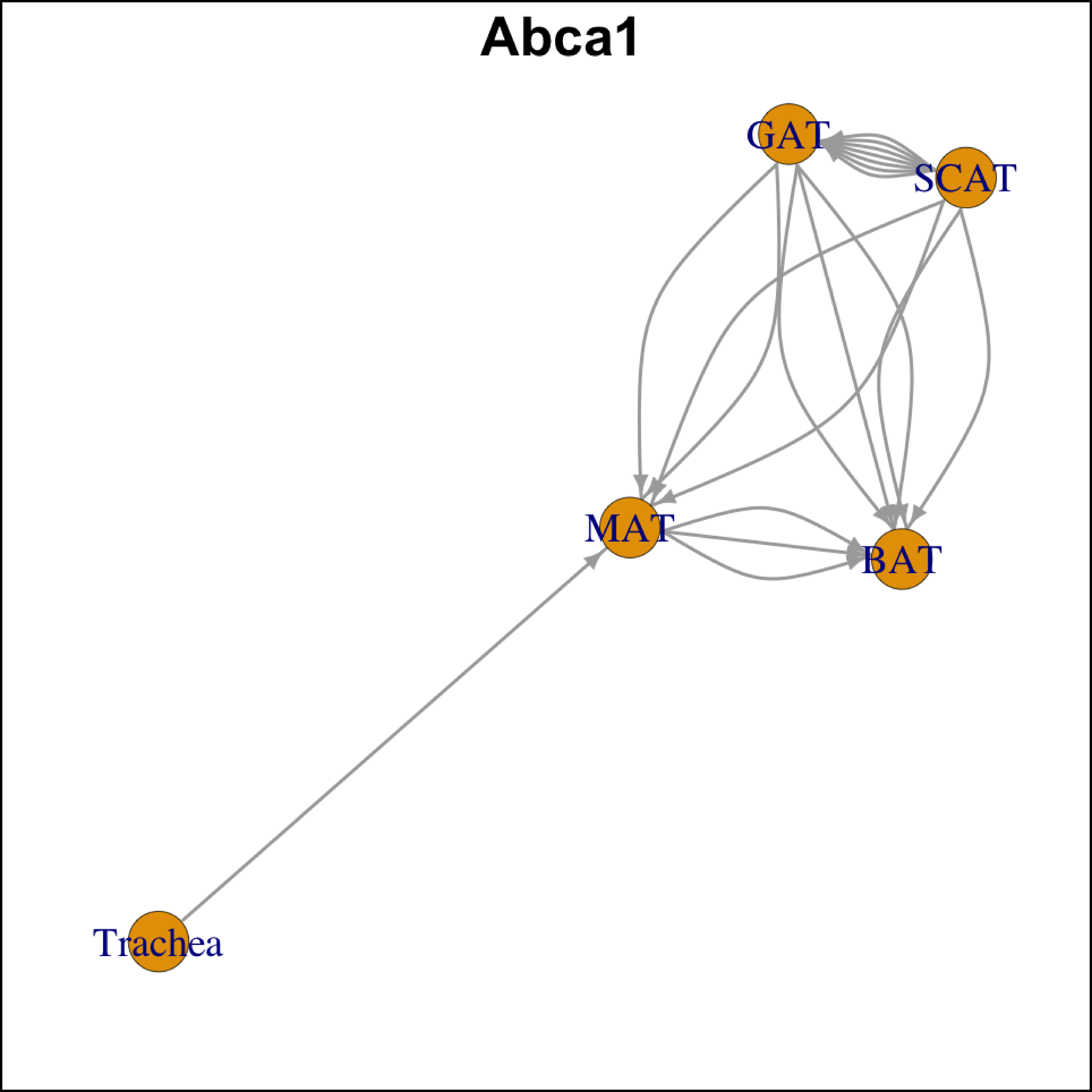
Abca1
GAT
SCAT
MAT
BAT
Trachea

Abca8a

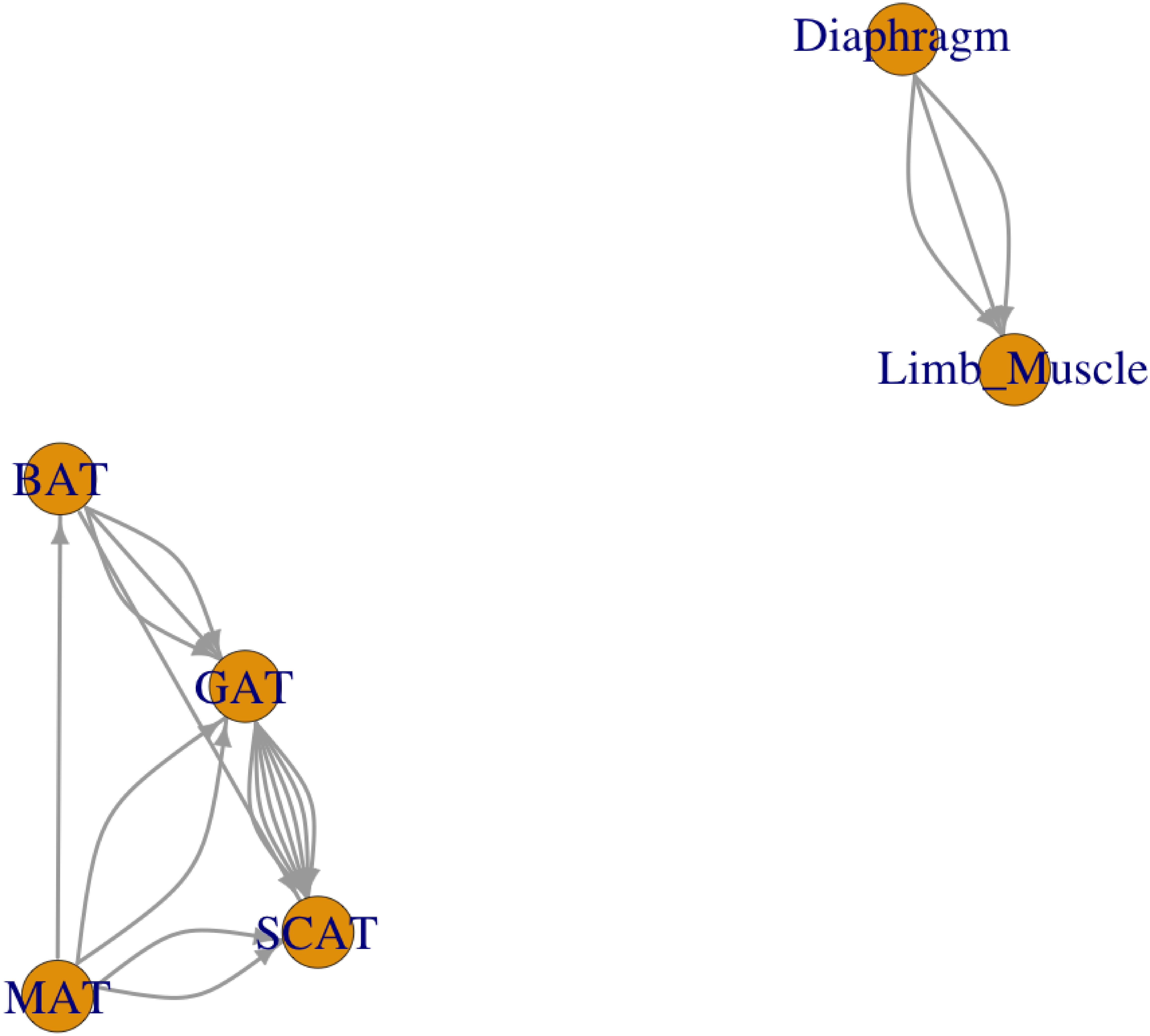

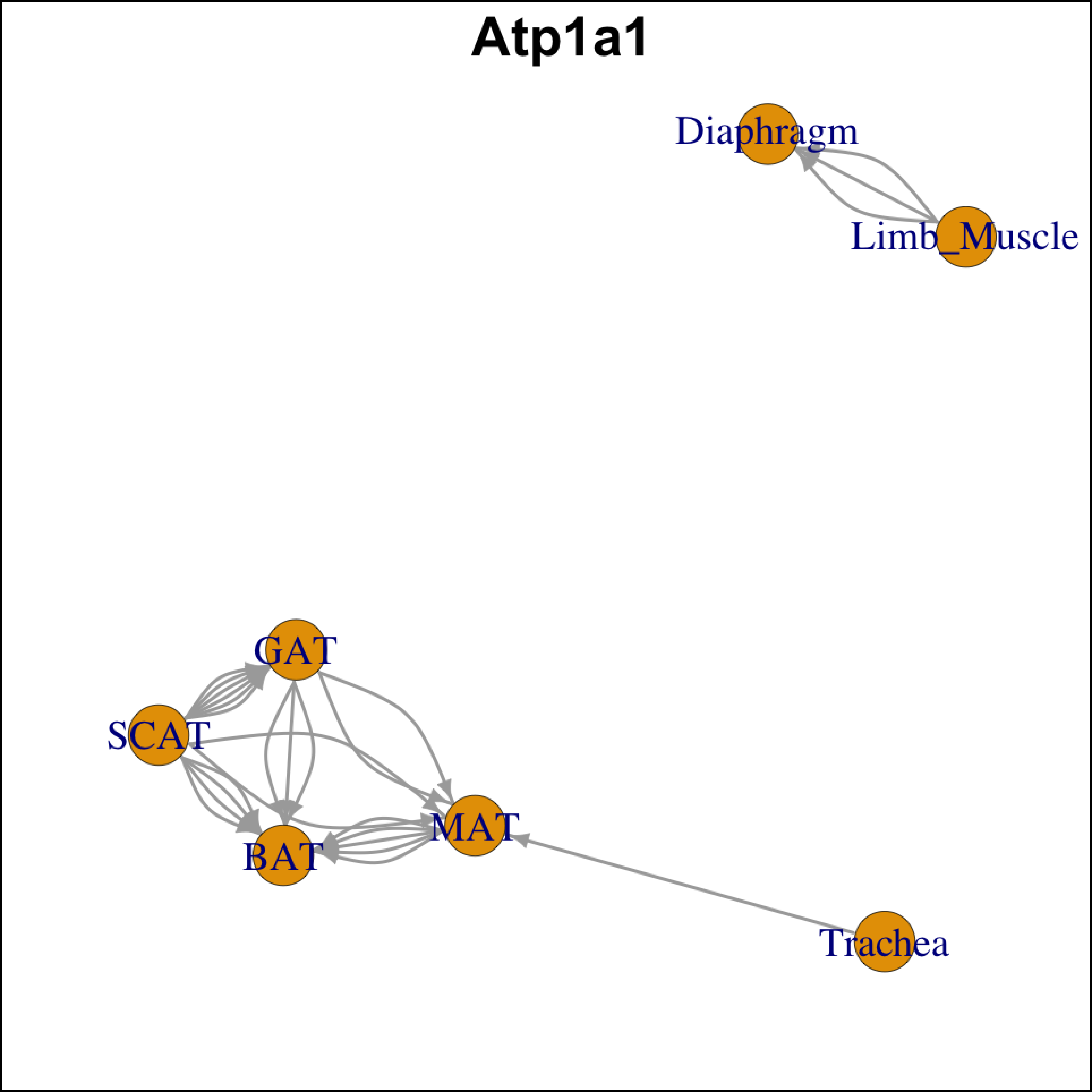
Atp1a1
Diaphragm
Limb_Muscle
GAT
SCAT
MAT
BAT
Trachea

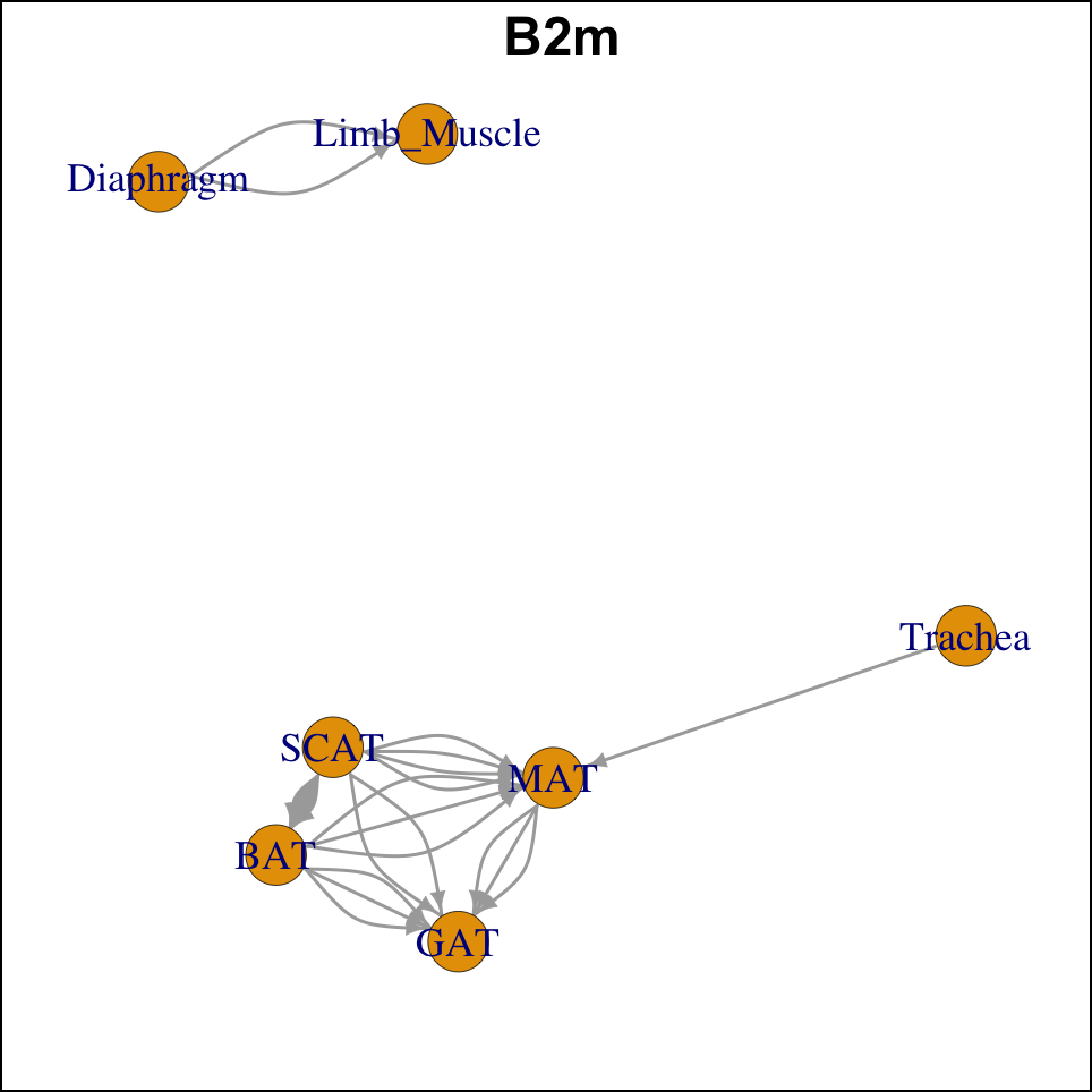
B2m
Limb_Muscle
Diaphragm
Trachea
SCAT
MAT
BAT
GAT

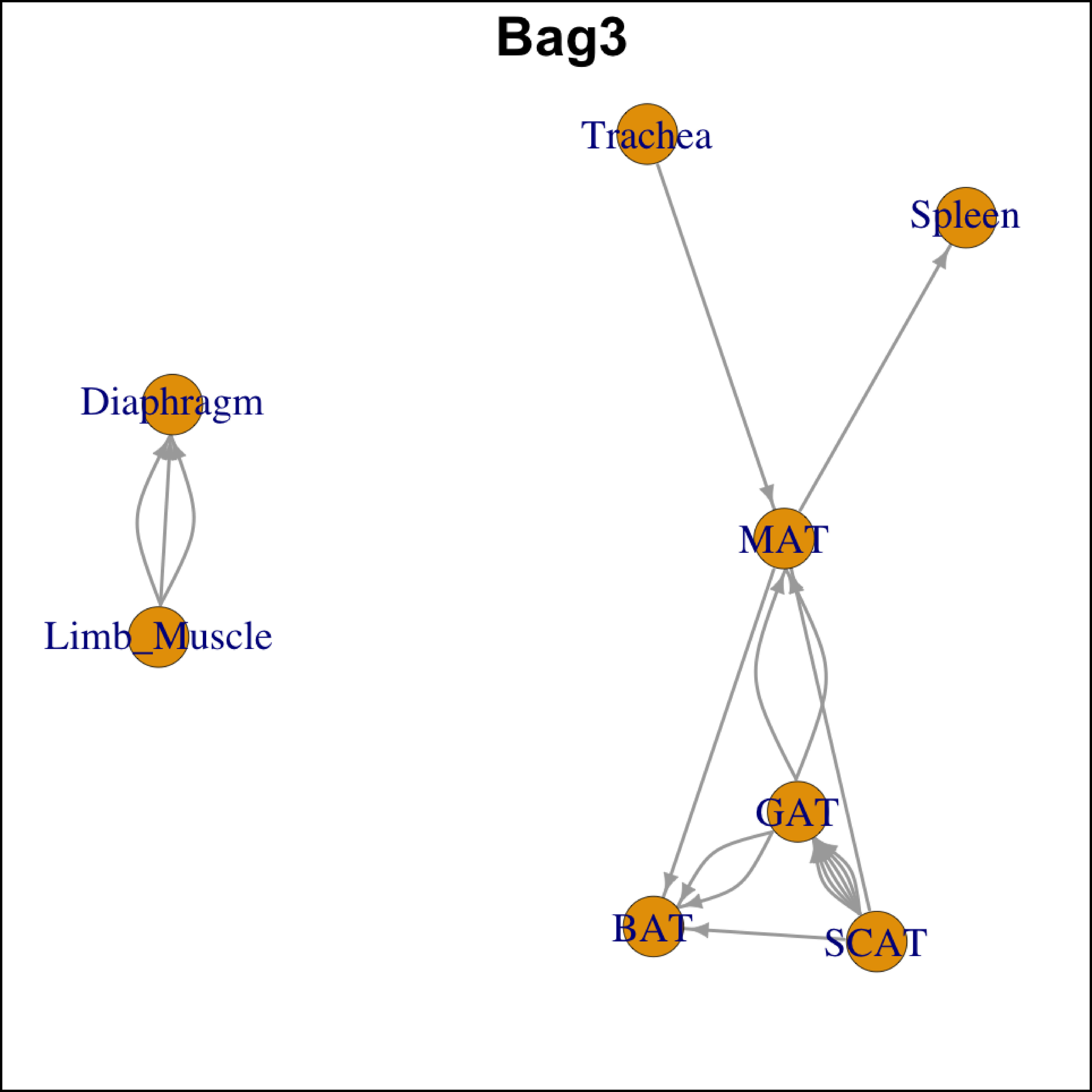
Bag3
Trachea
Spleen
Diaphragm
MAT
Limb_Muscle
GAT
BAT
SCAT

Bzw1

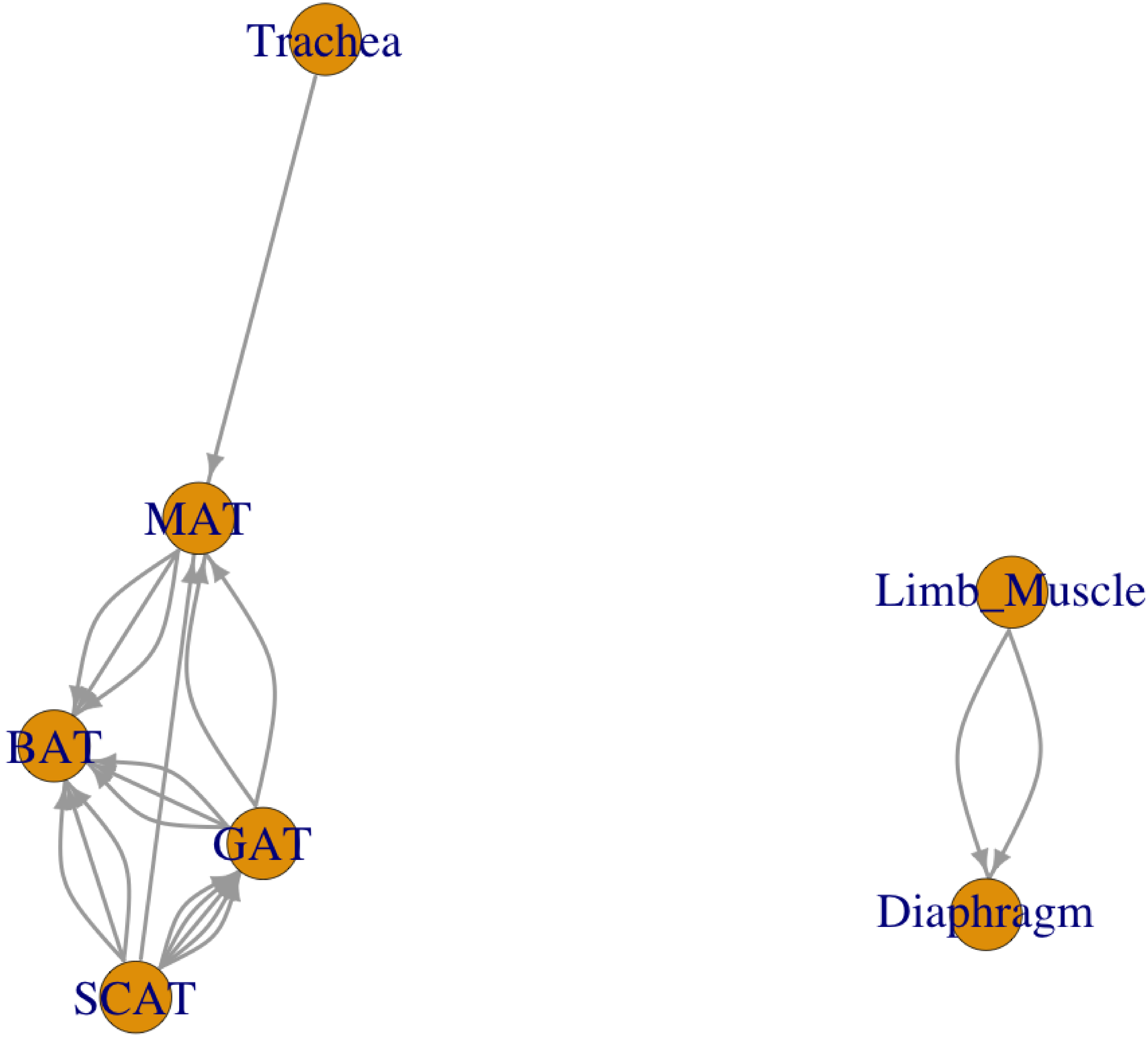

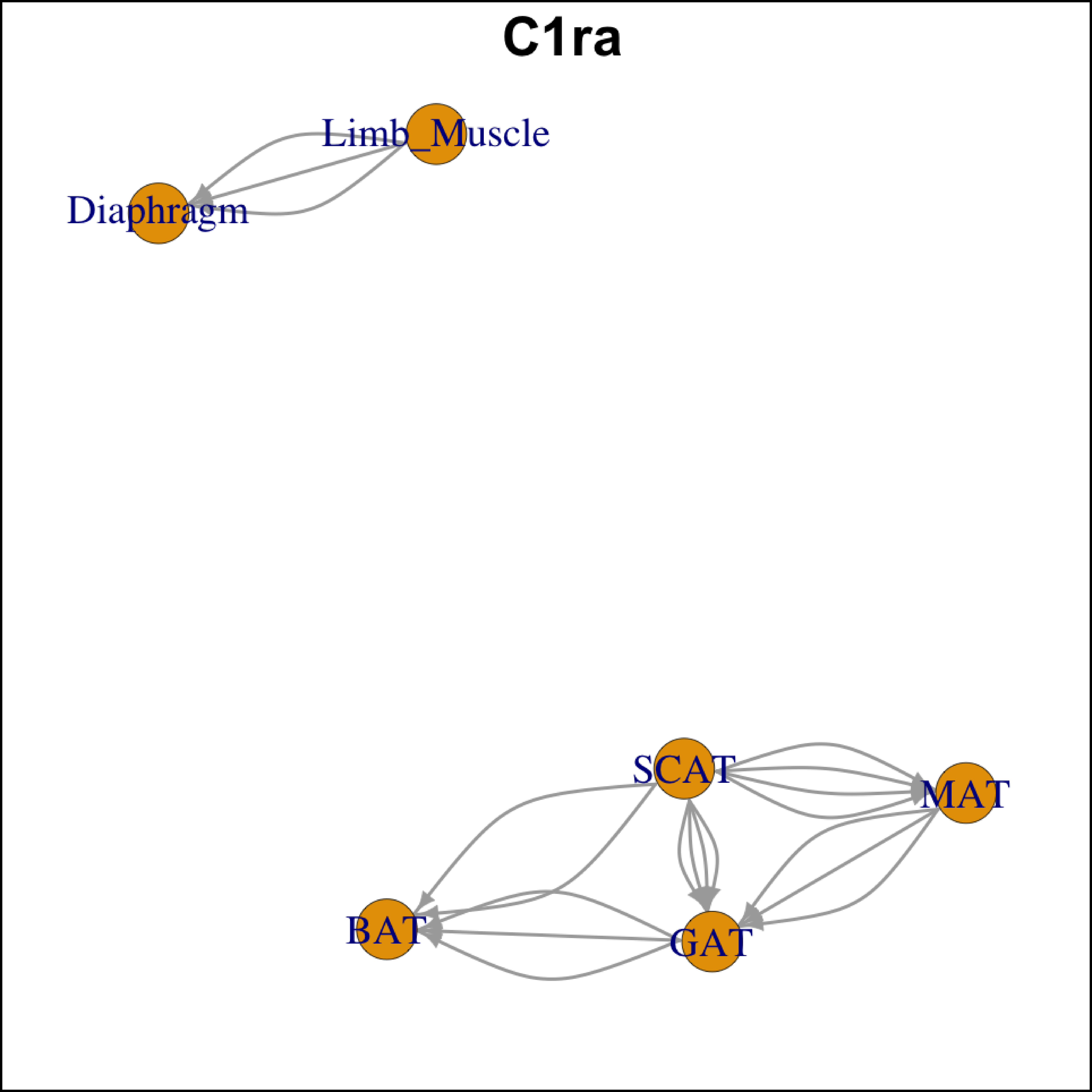

C1ra
Limb_Muscle
Diaphragm
SCAT
MAT
BAT
GAT

Calu

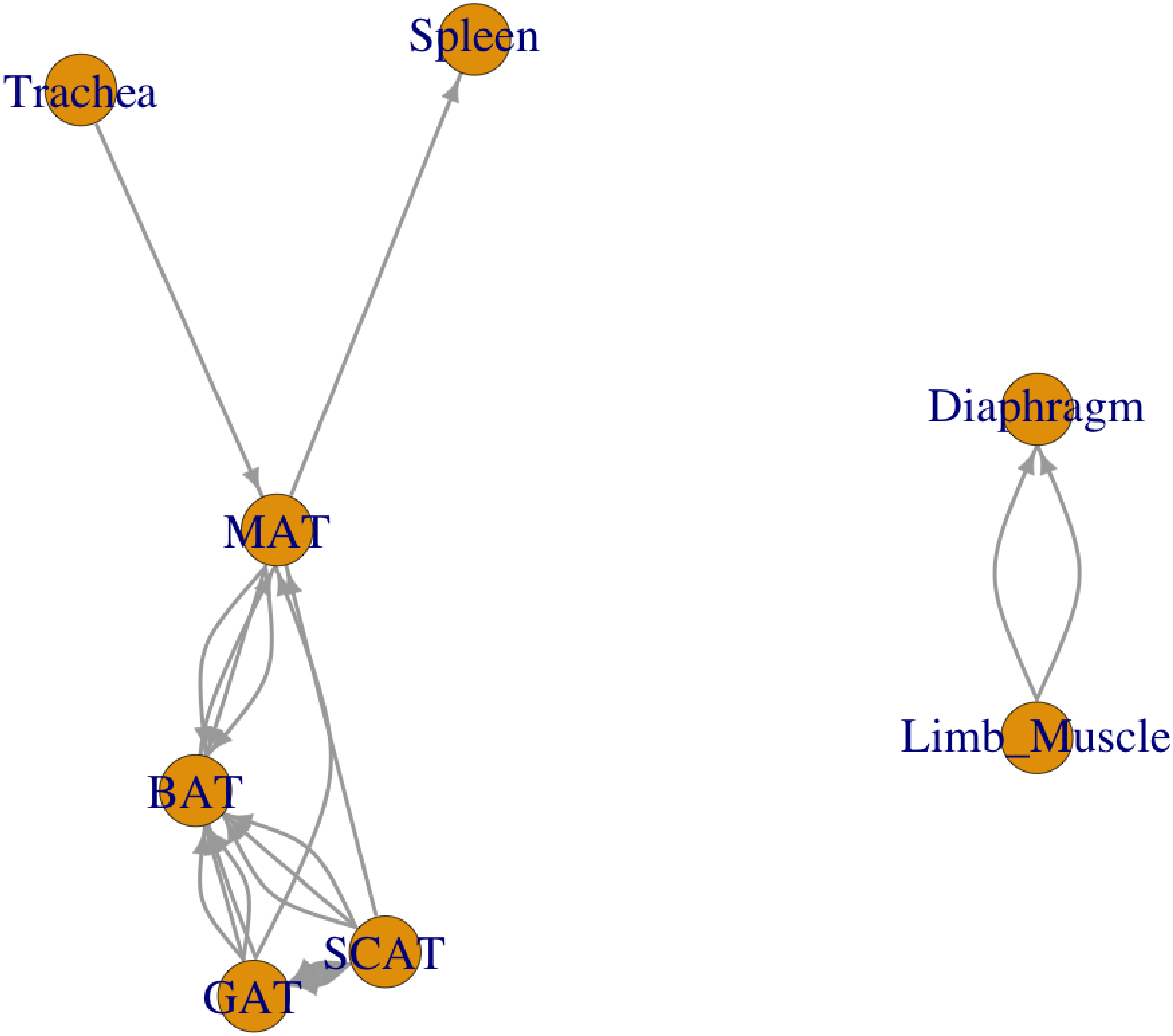

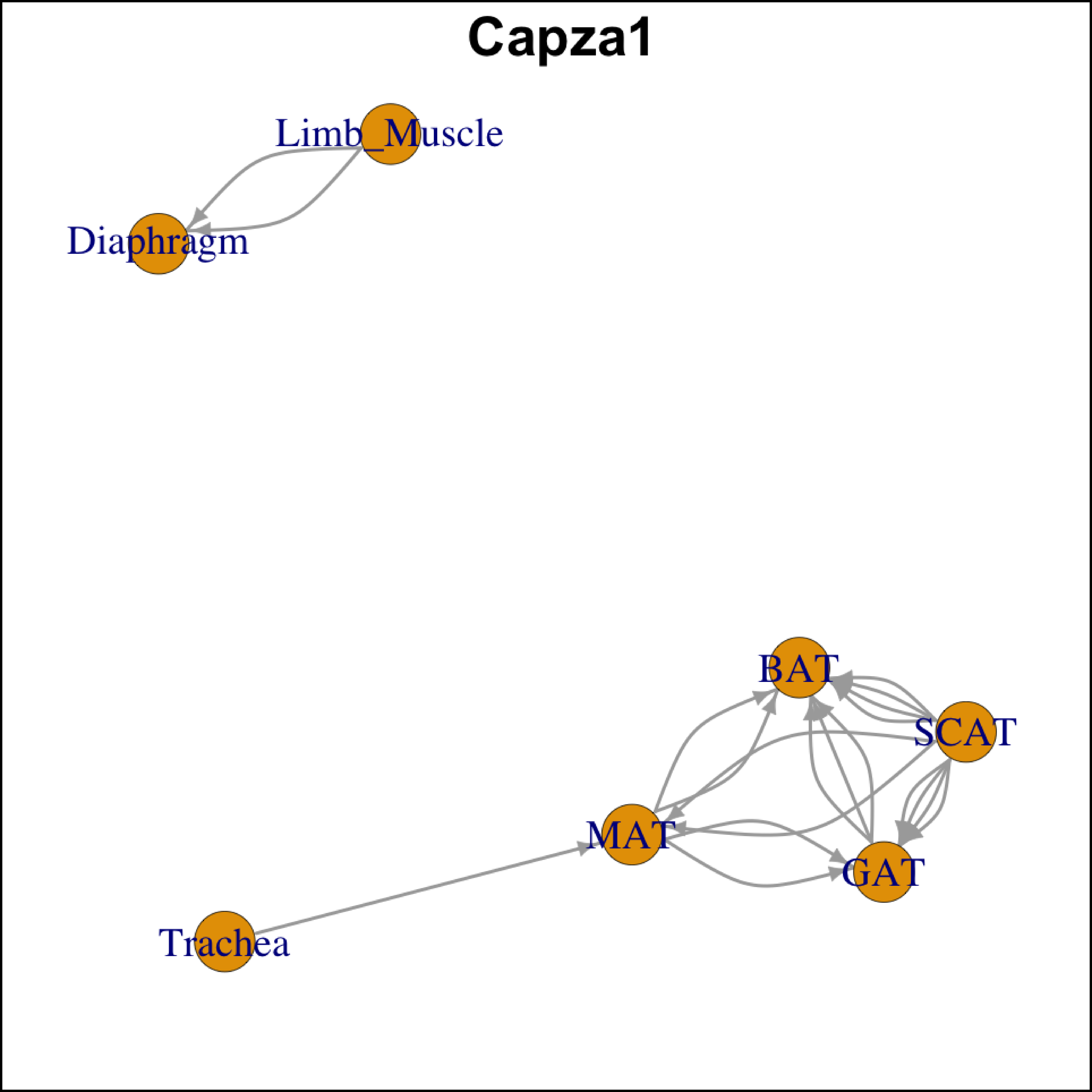
Capza1
Limb_Muscle
Diaphragm
BAT
SCAT
MAT
GAT
Trachea

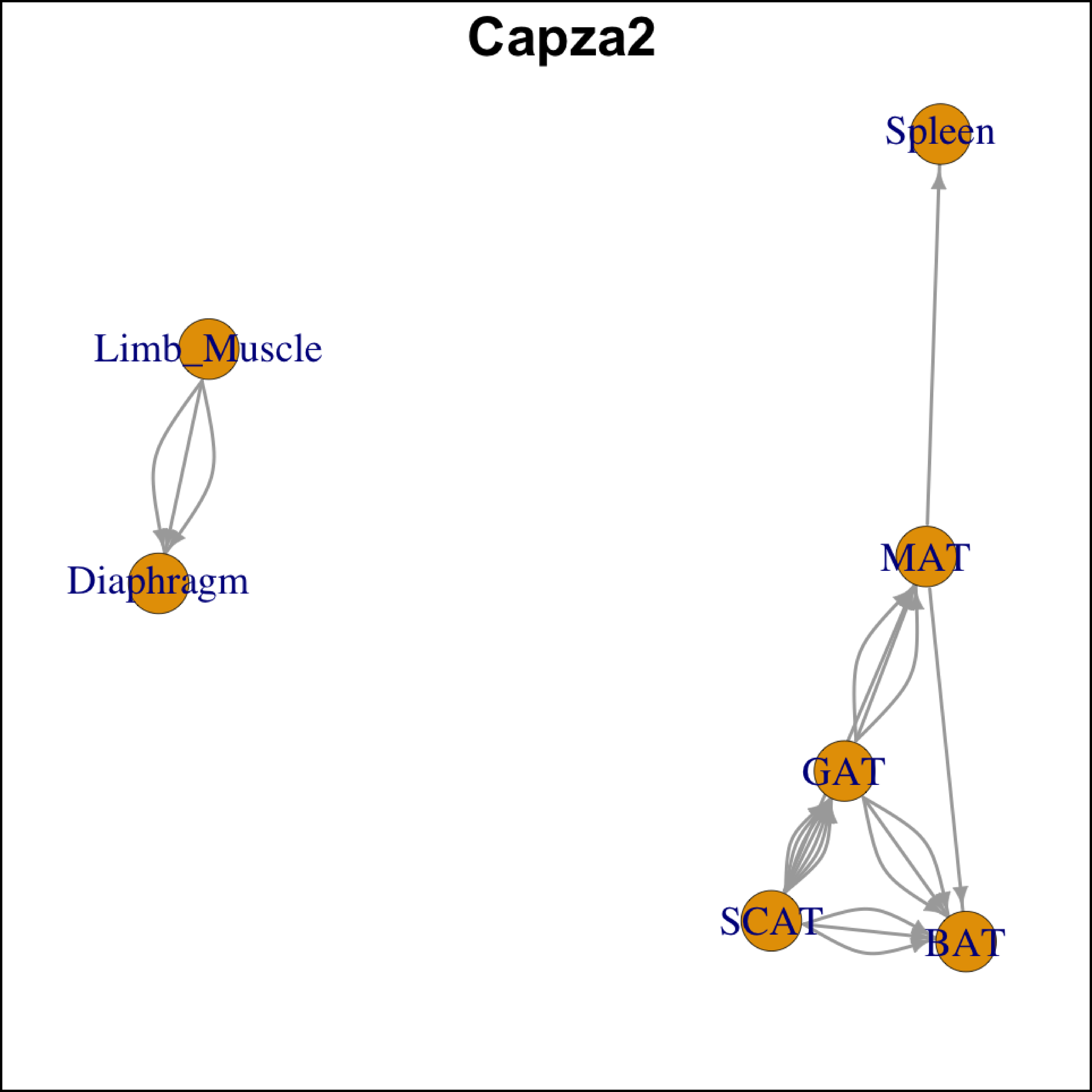
Capza2
Spleen
Limb_Muscle
MAT
Diaphragm
GAT
SCAT
BAT

Cd38

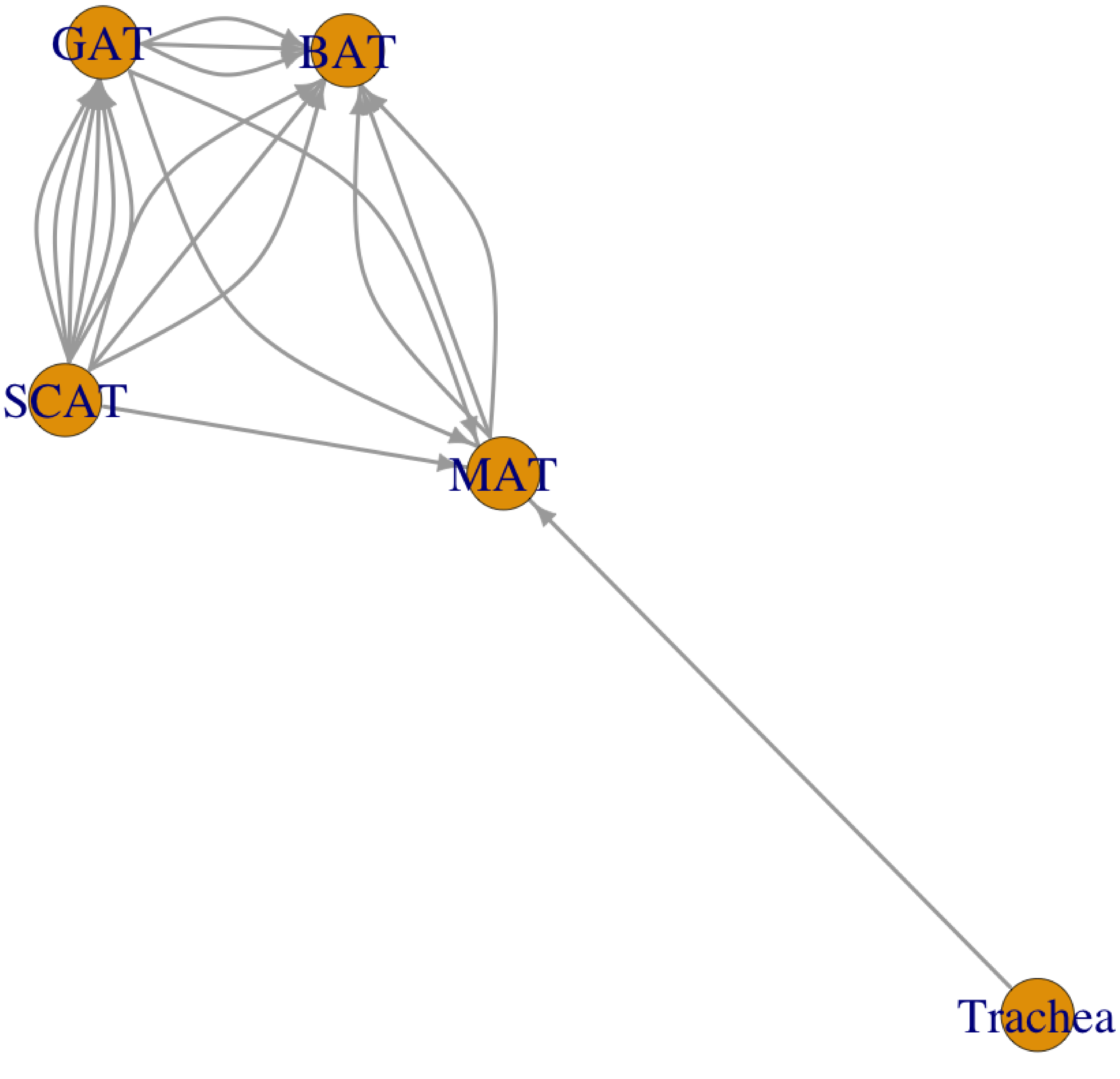

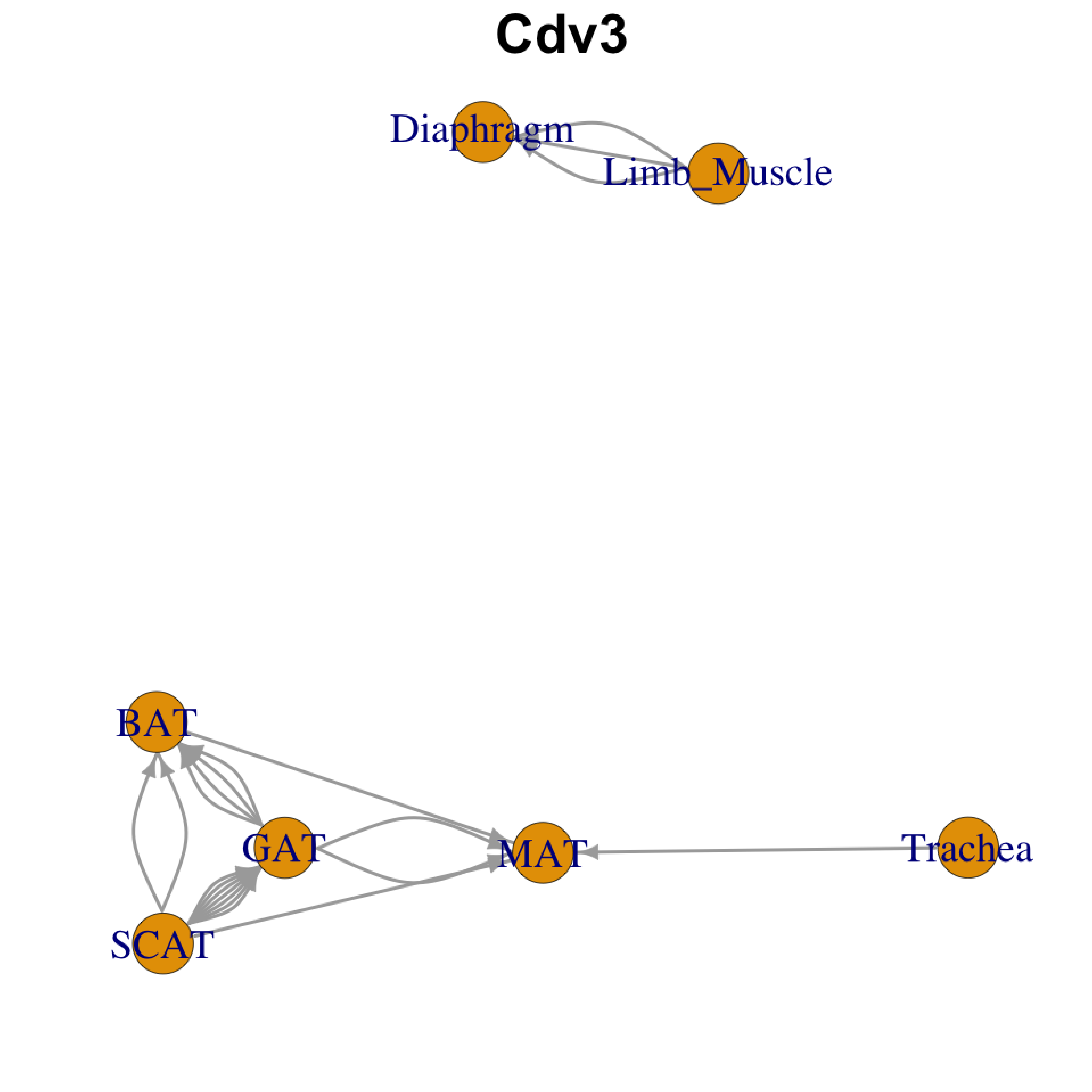
Cdv3
Diaphragm
Limb_Muscle
BAT
GAT
MAT
Trachea
SCAT

Cfb

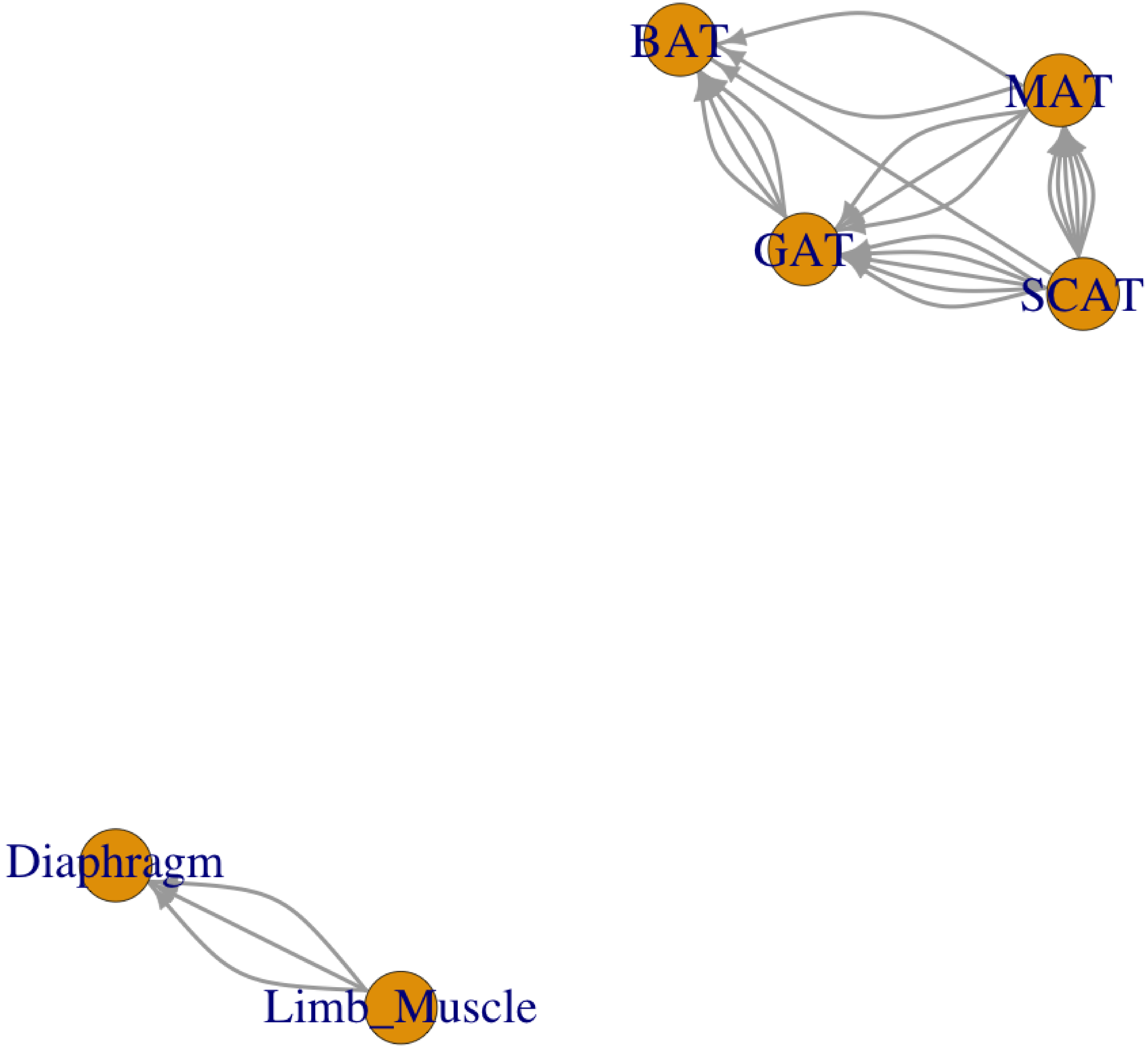

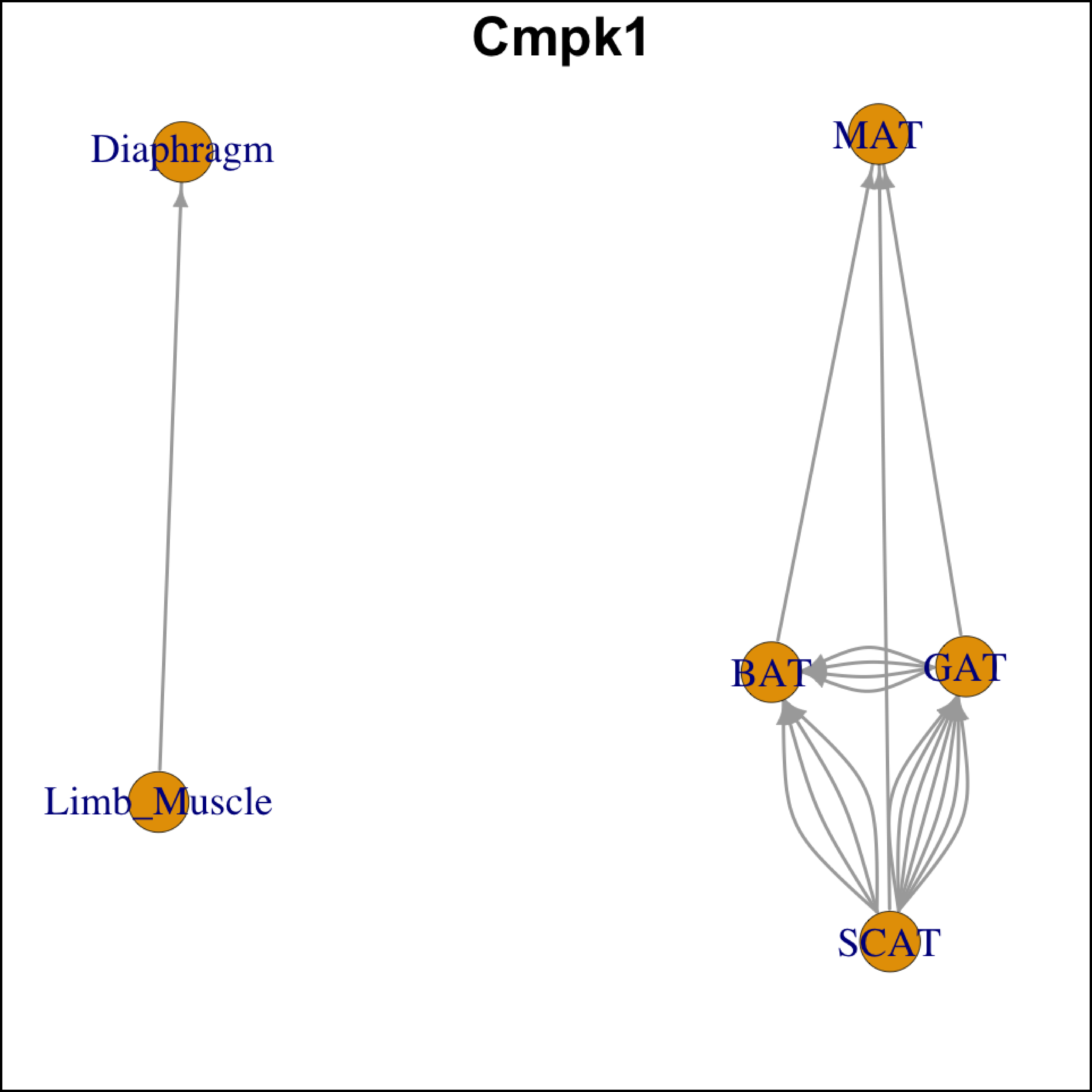
Cmpk1
Diaphragm
MAT
Limb_Muscle
BAT
GAT
SCAT

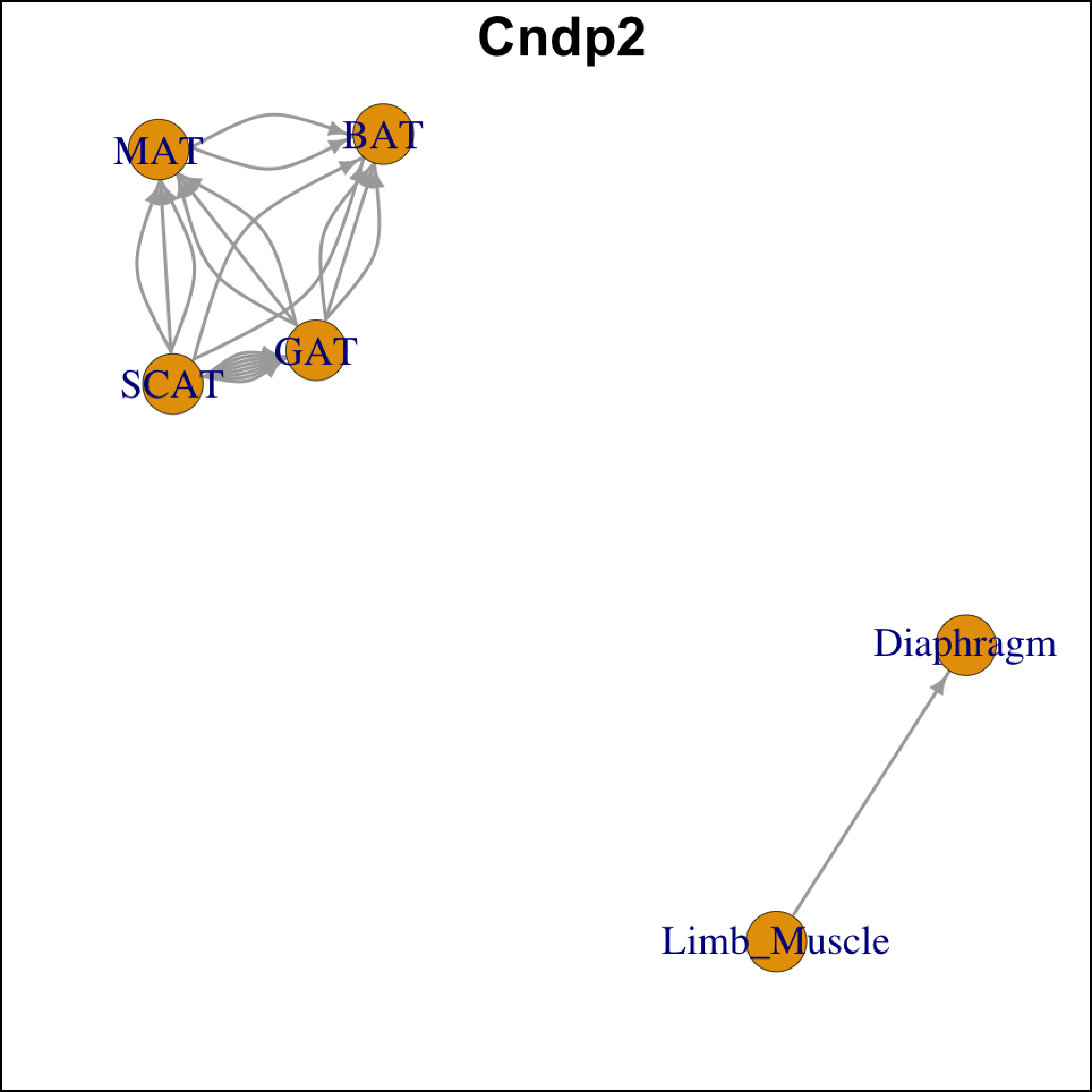
Cndp2
MAT
BAT
GAT
SCAT
Diaphragm
Limb_Muscle

Col1a1

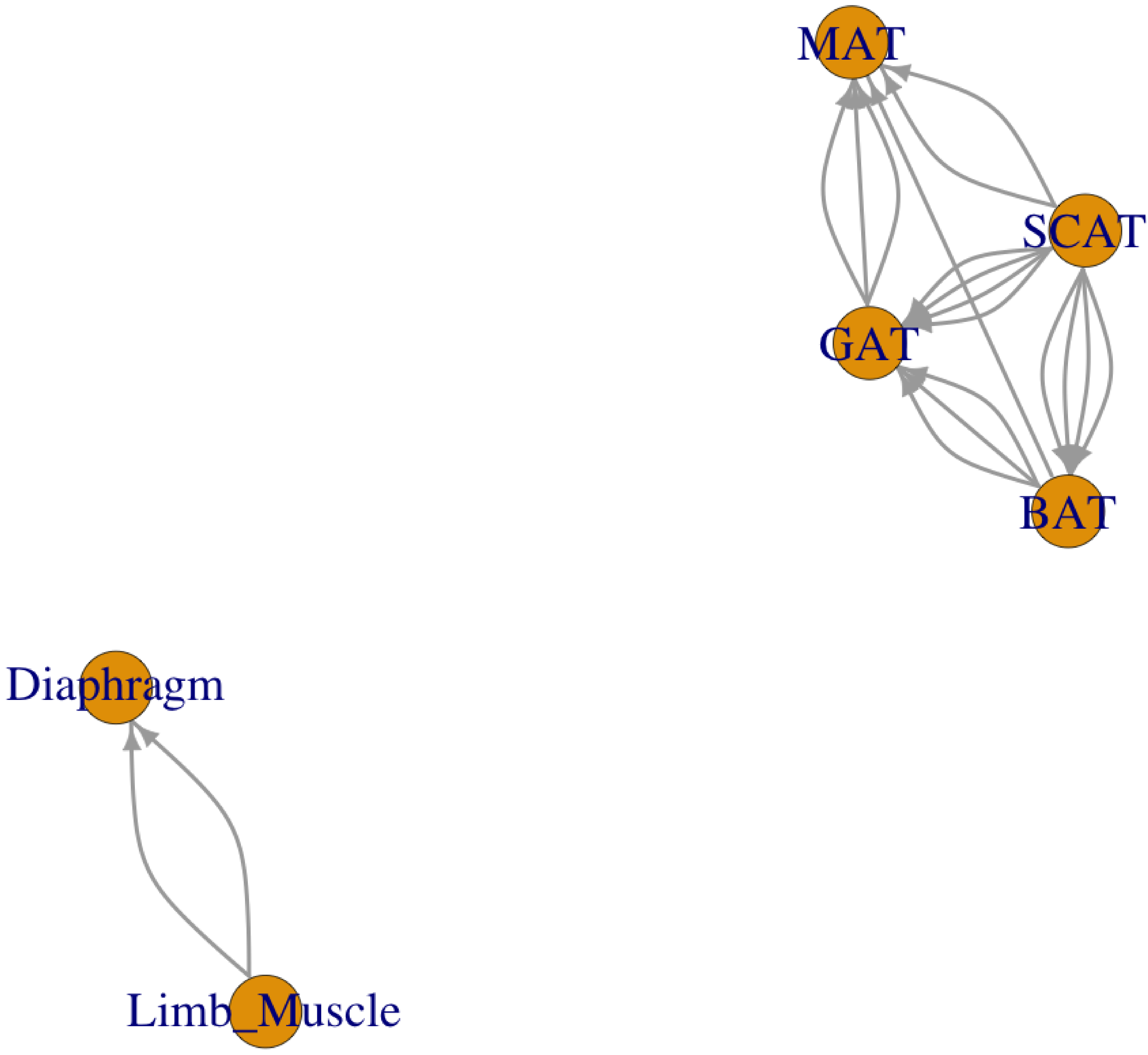

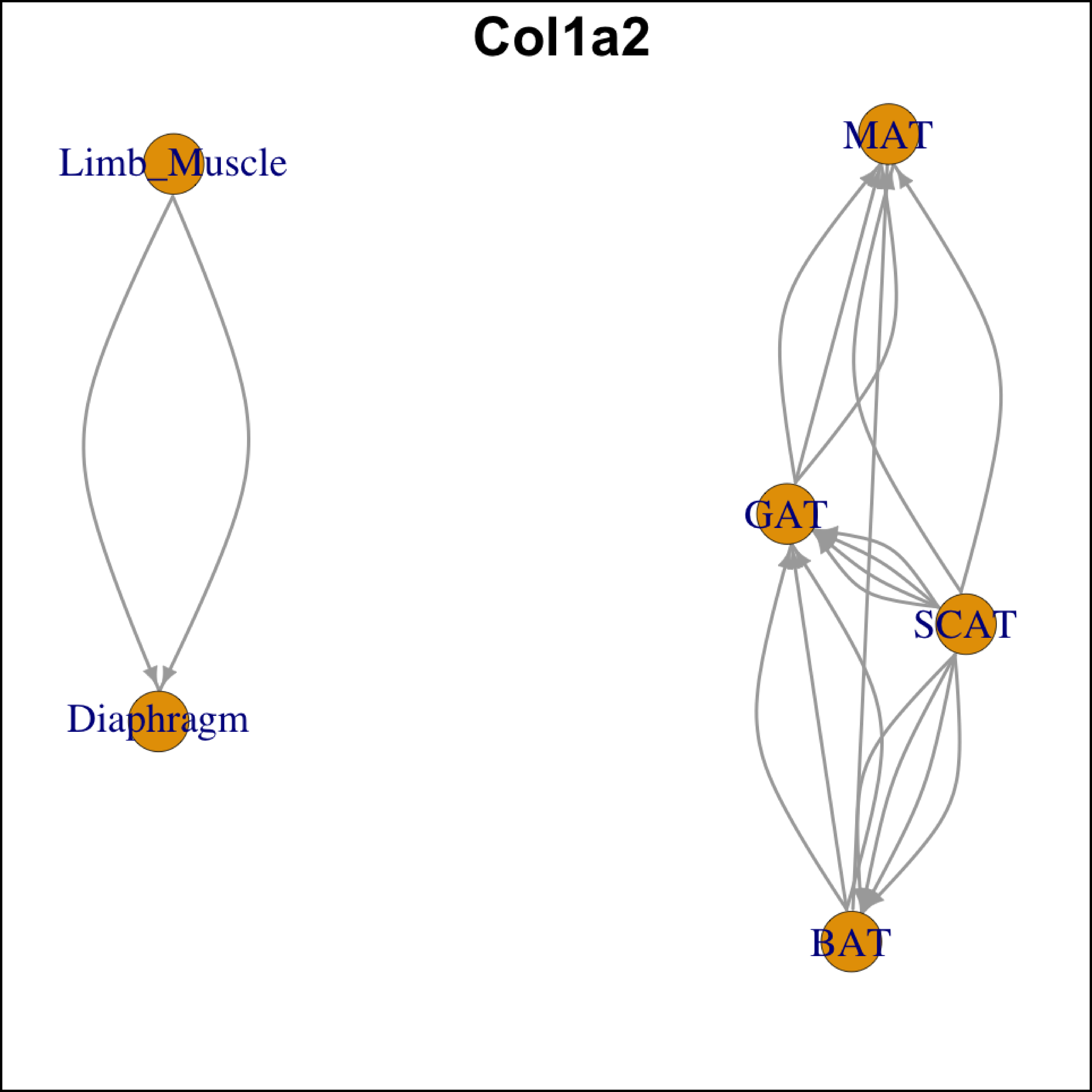
Col1a2
Limb_Muscle
Diaphragm
MAT
GAT
SCAT
BAT

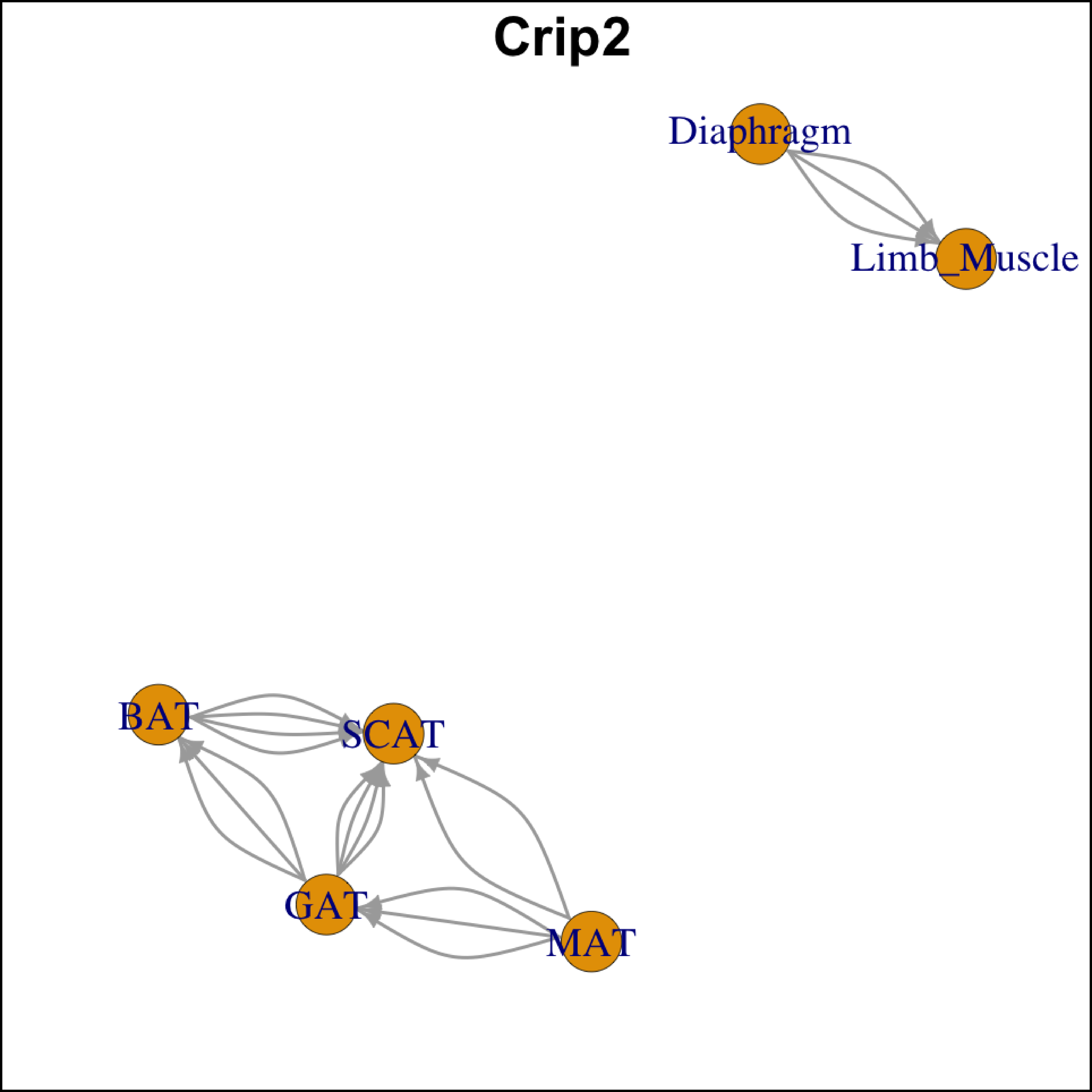
Crip2
Diaphragm
Limb_Muscle
BAT
SCAT
GAT
MAT

Cyb561

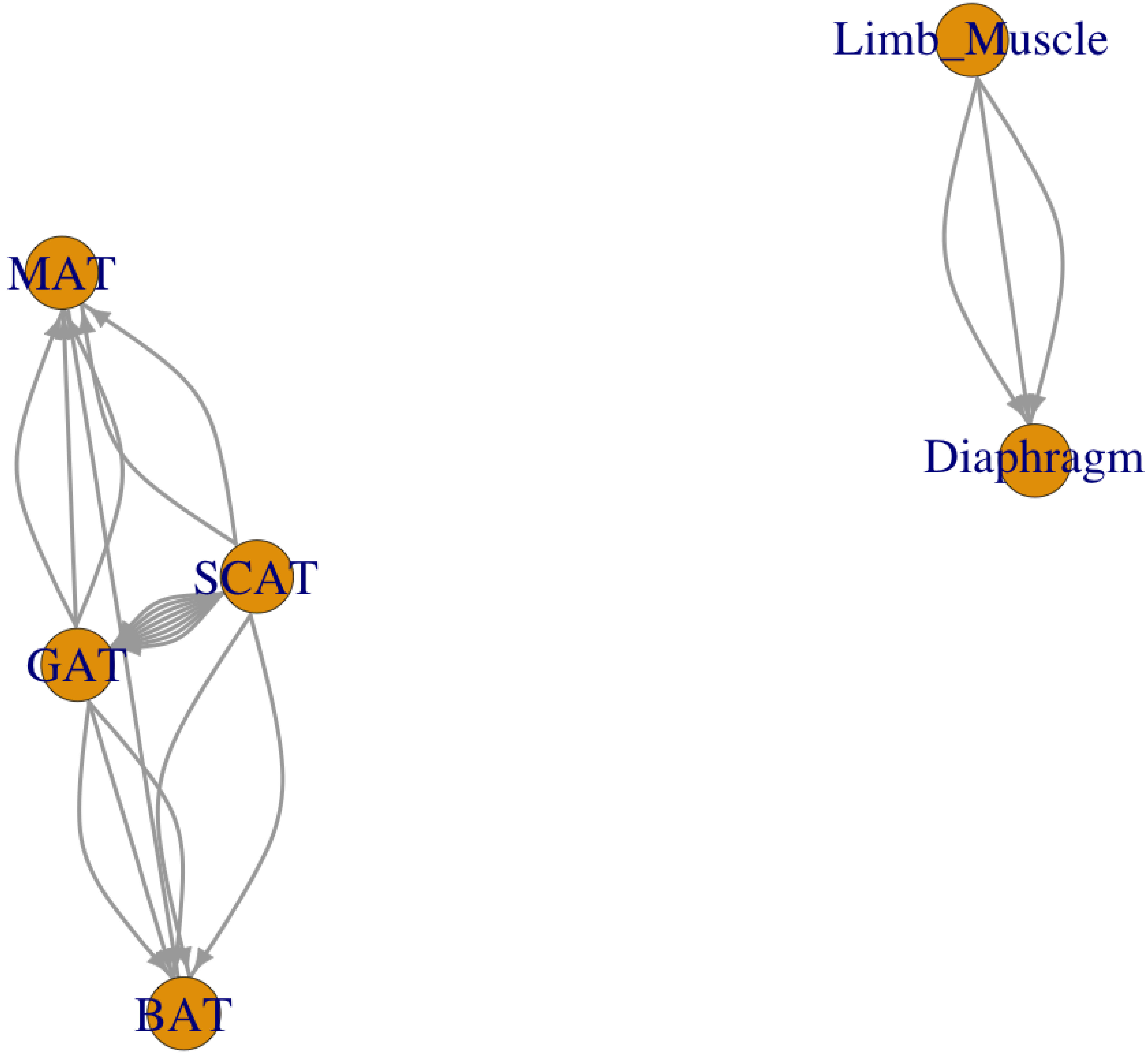

Cyb5r3

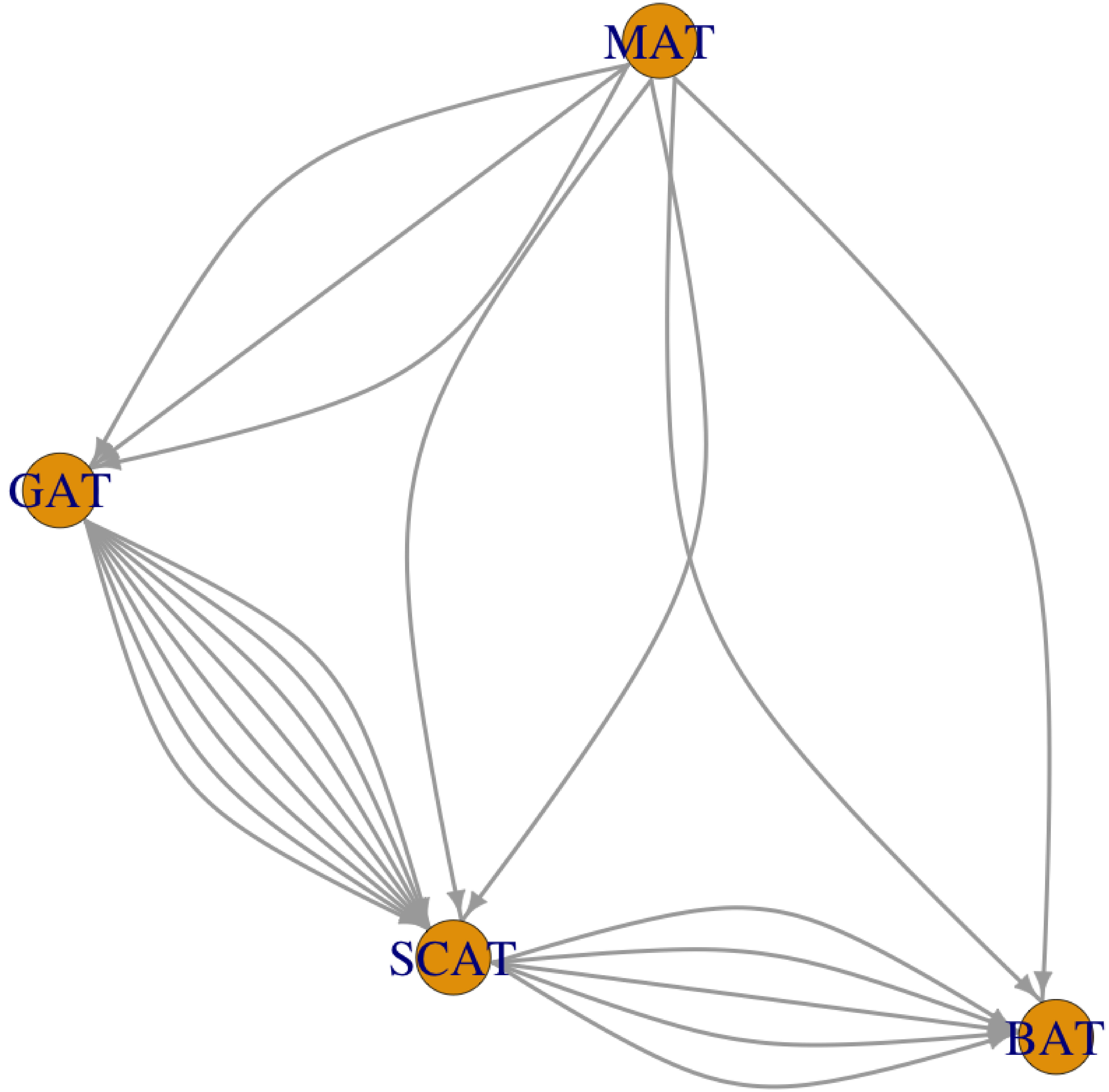

Dcn

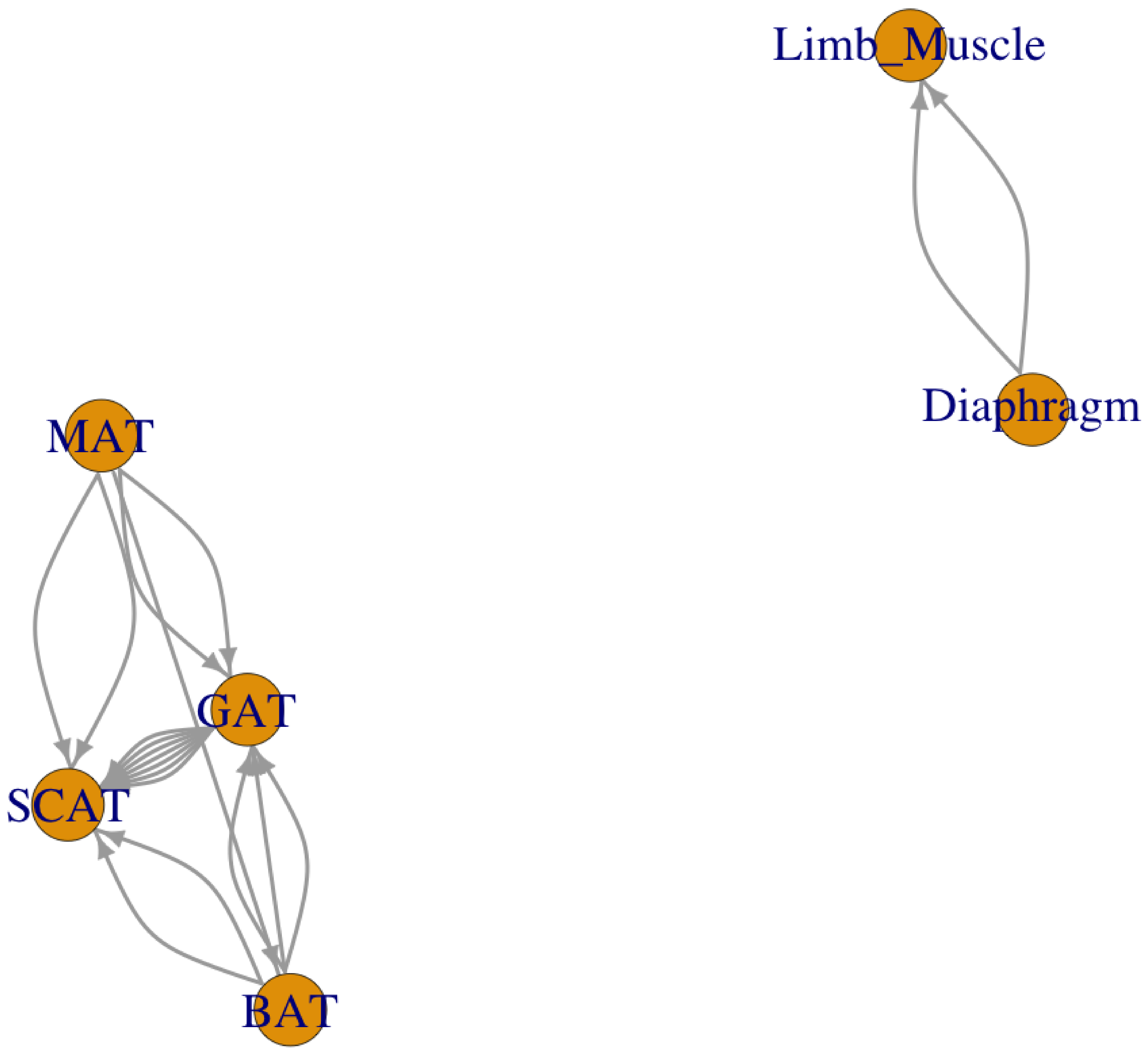

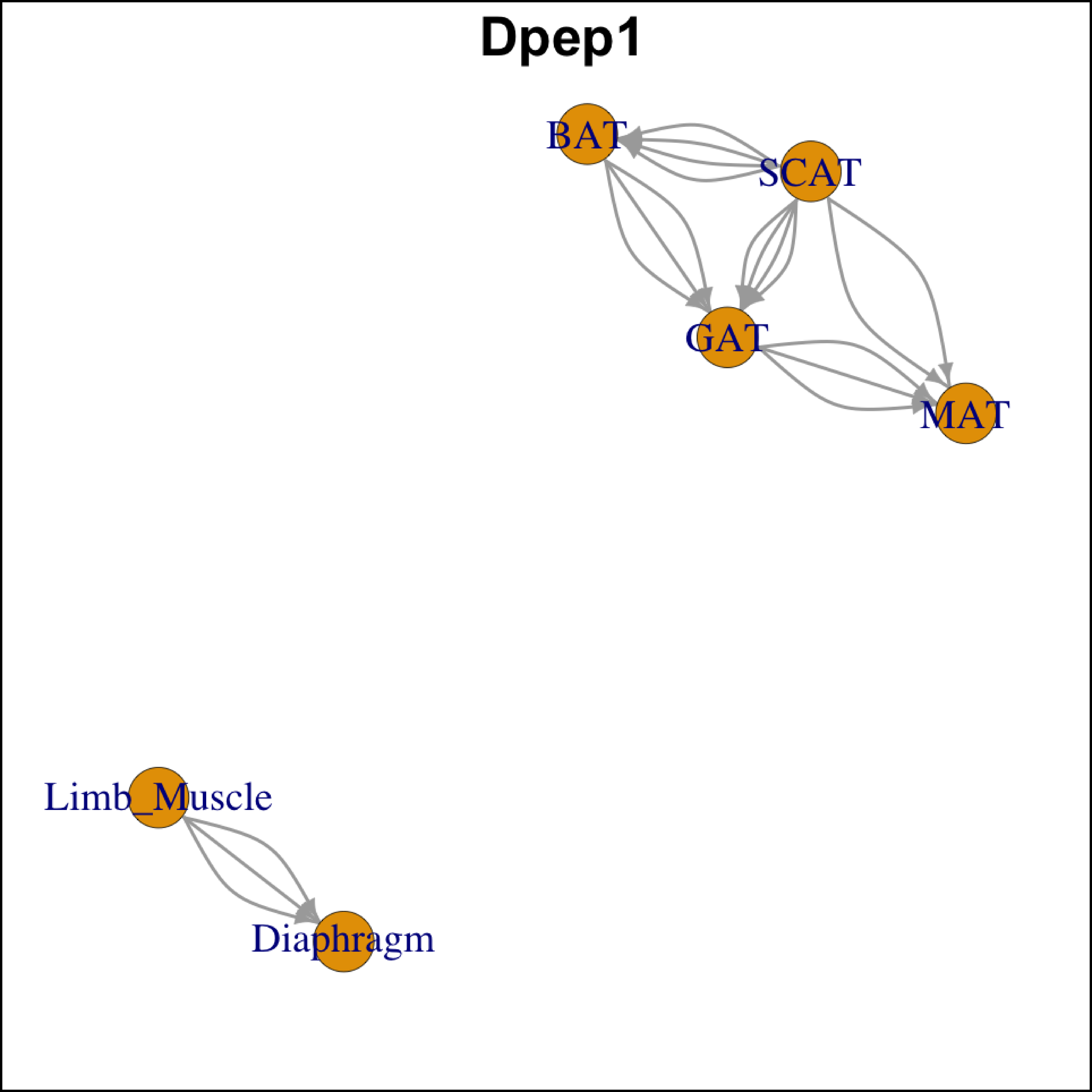
Dpep1
BAT
SCAT
GAT
MAT
Limb_Muscle
Diaphragm

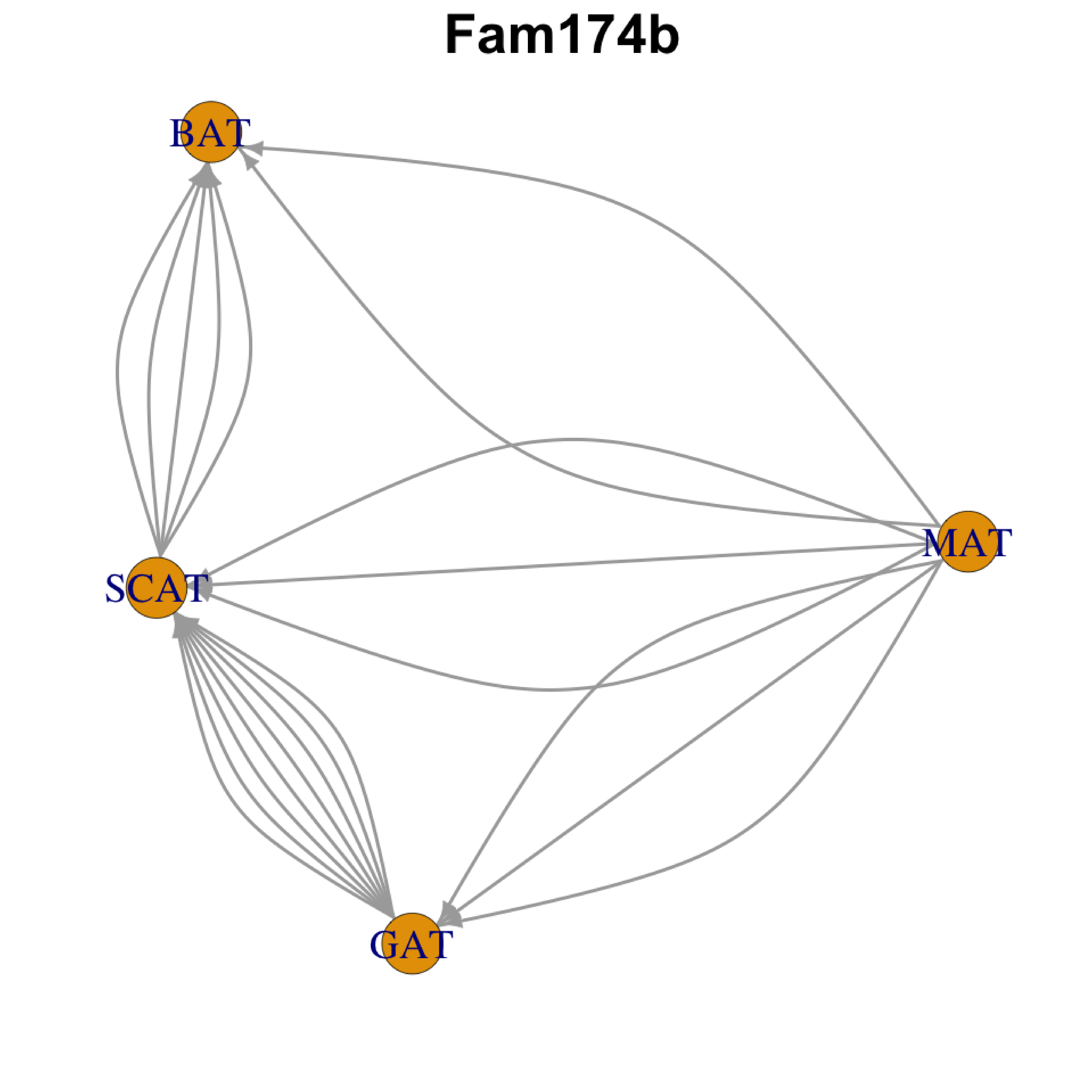
Fam174b
BAT
MAT
SCAT
GAT

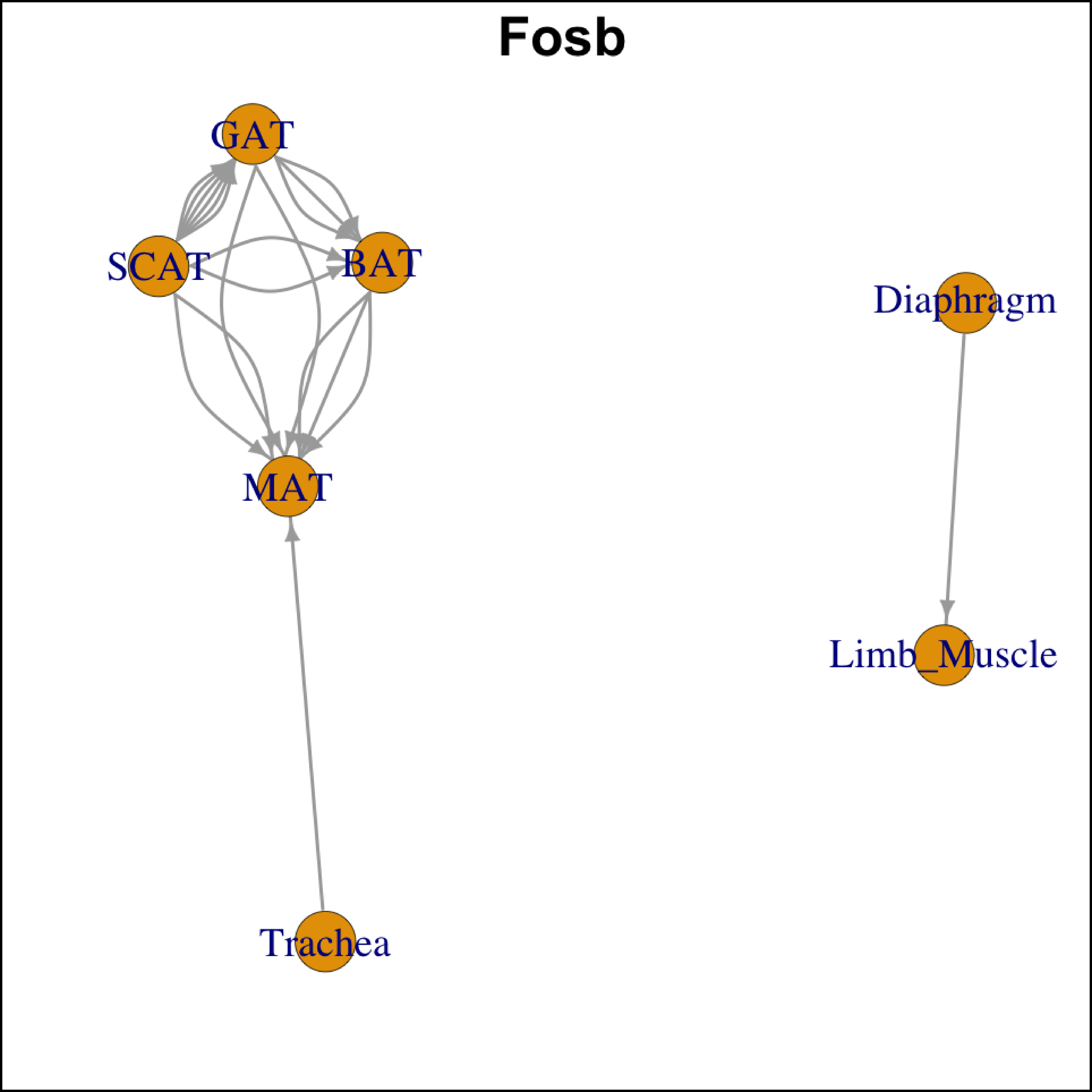
Fosb
GAT
SCAT
BAT
Diaphragm
MAT
Limb_Muscle
Trachea

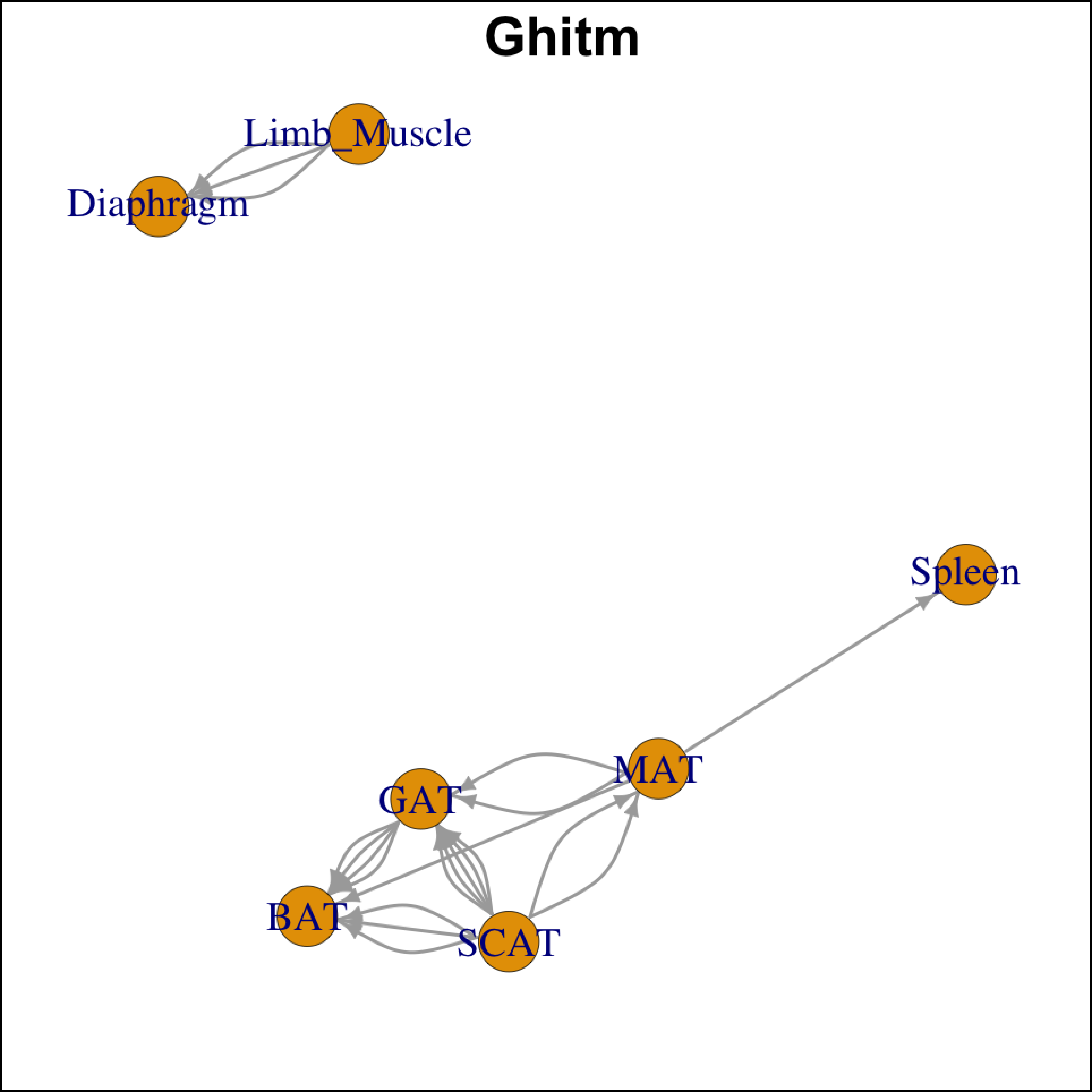
Ghitm
Limb_Muscle
Diaphragm
Spleen
MAT
GAT
BAT
SCAT

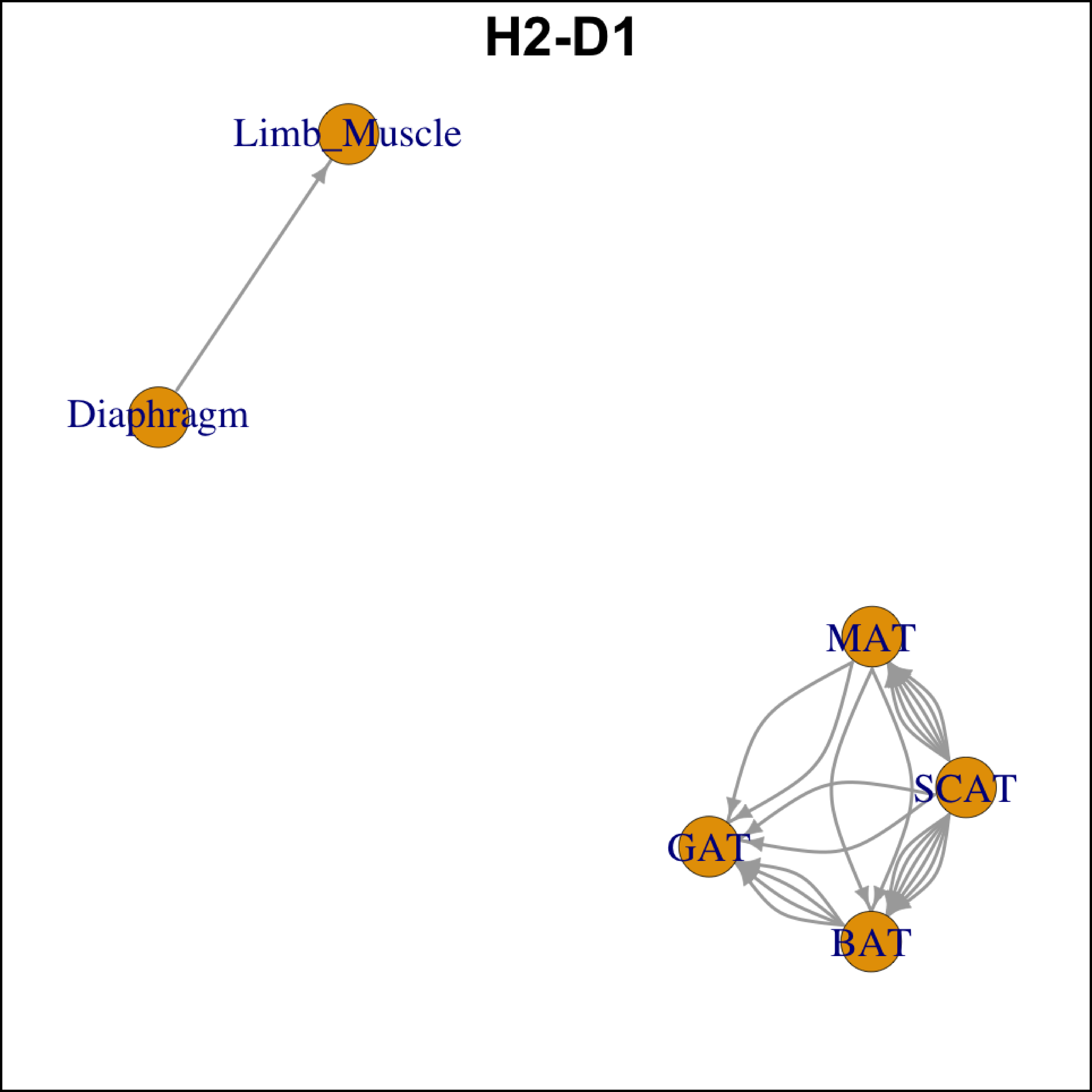
H2-D1
Limb_Muscle
Diaphragm
MAT
SCAT
GAT
BAT

Hnrnpf

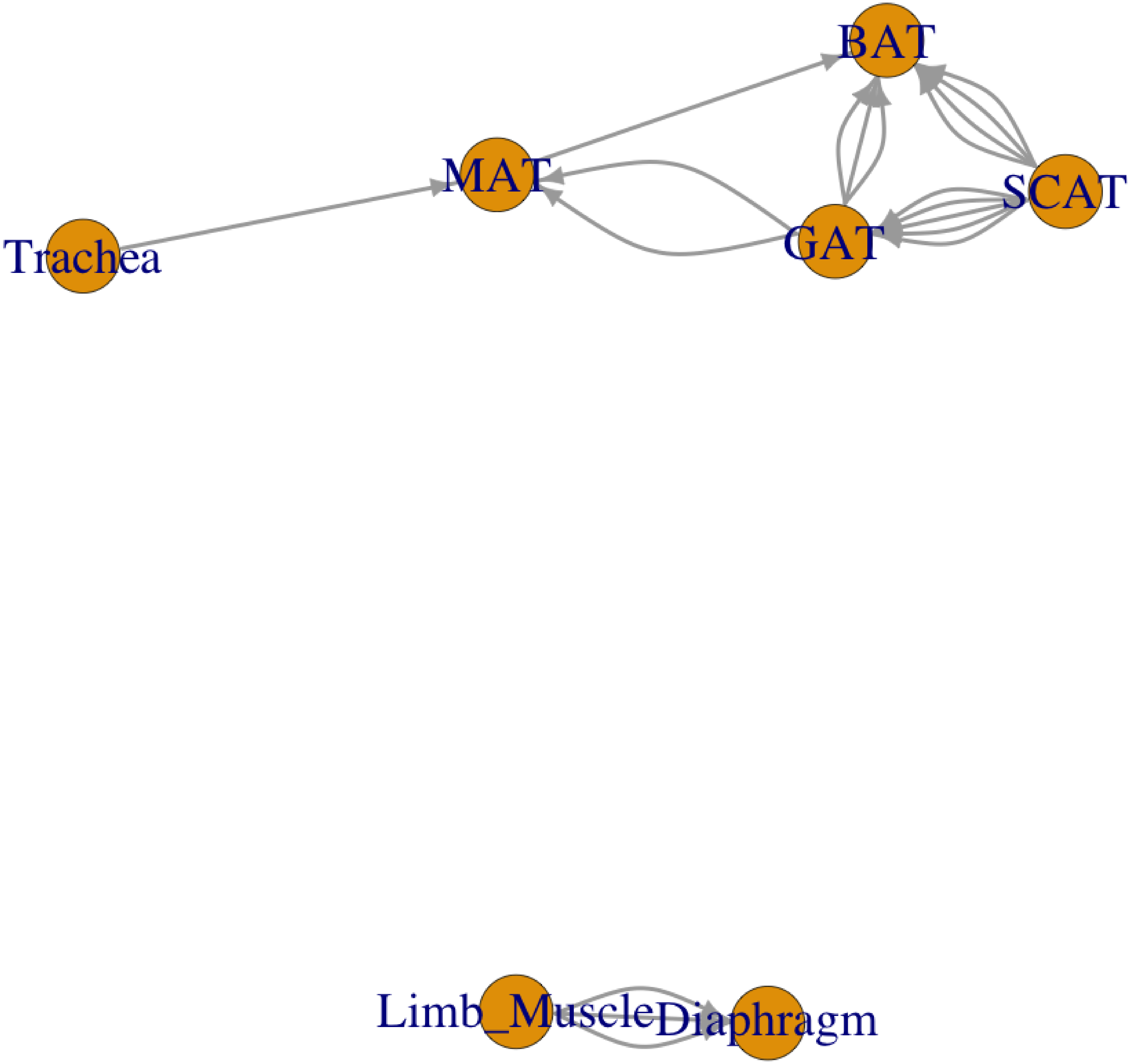

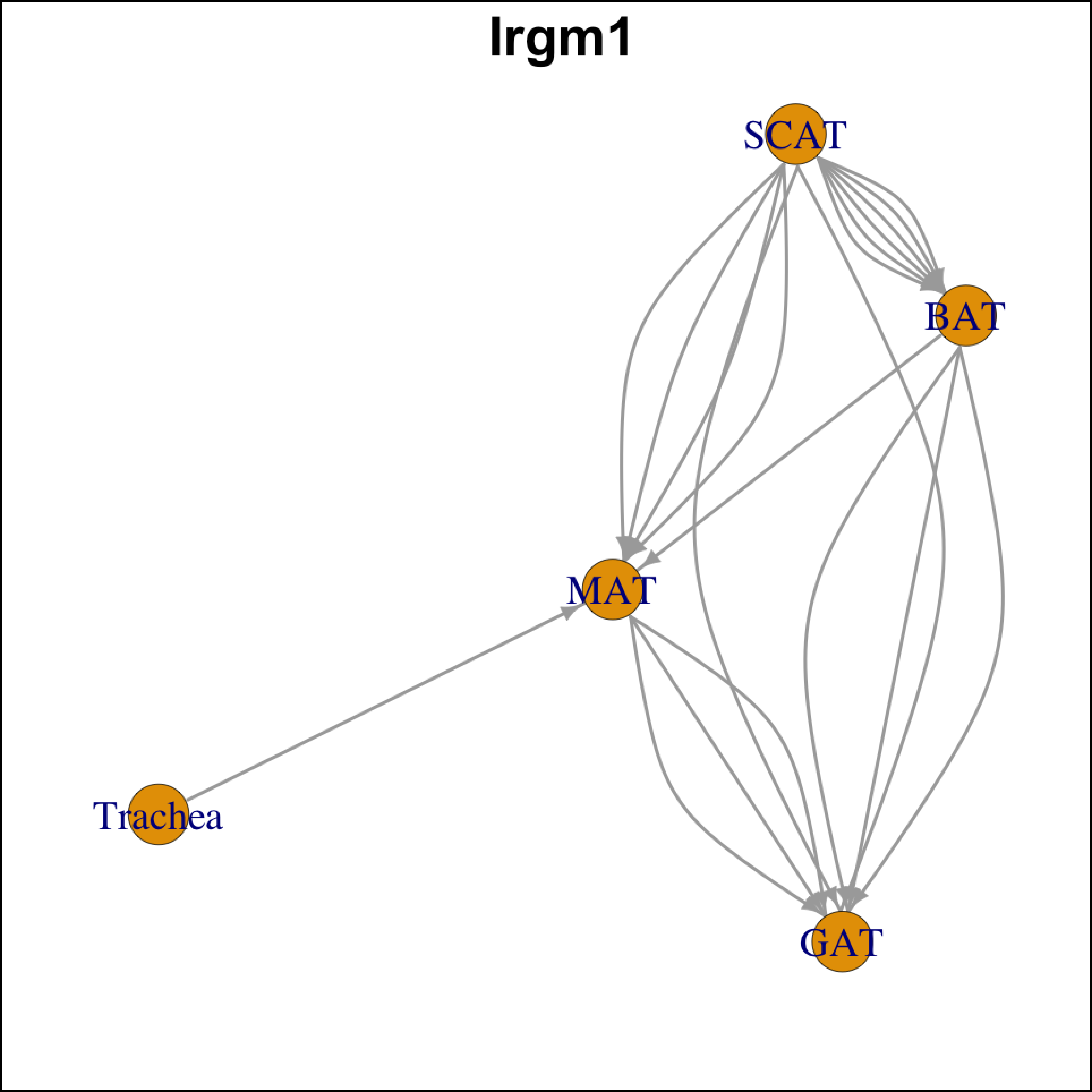
Irgm1
SCAT
BAT
MAT
Trachea
GAT

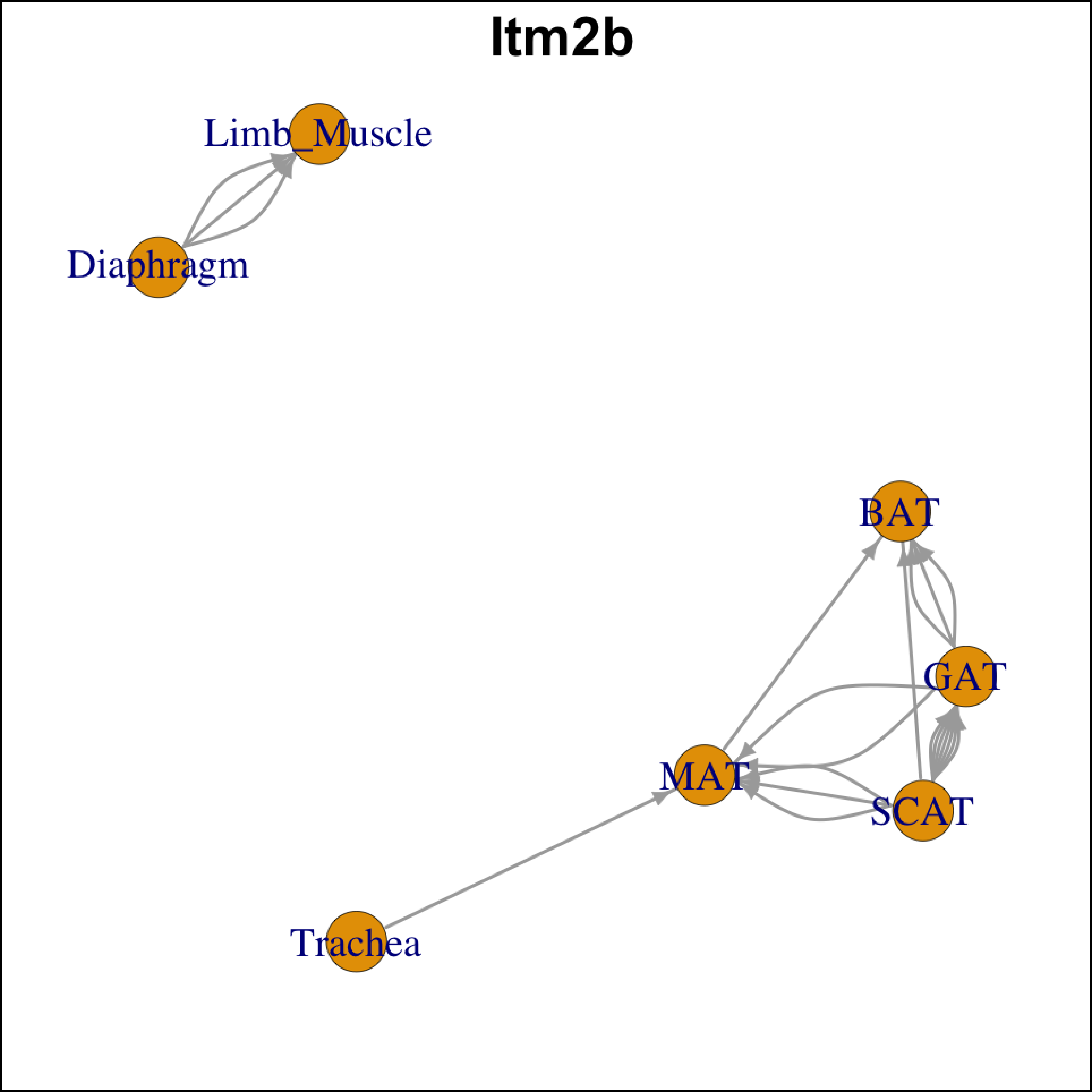
Itm2b
Limb_Muscle
Diaphragm
BAT
GAT
MAT
SCAT
Trachea

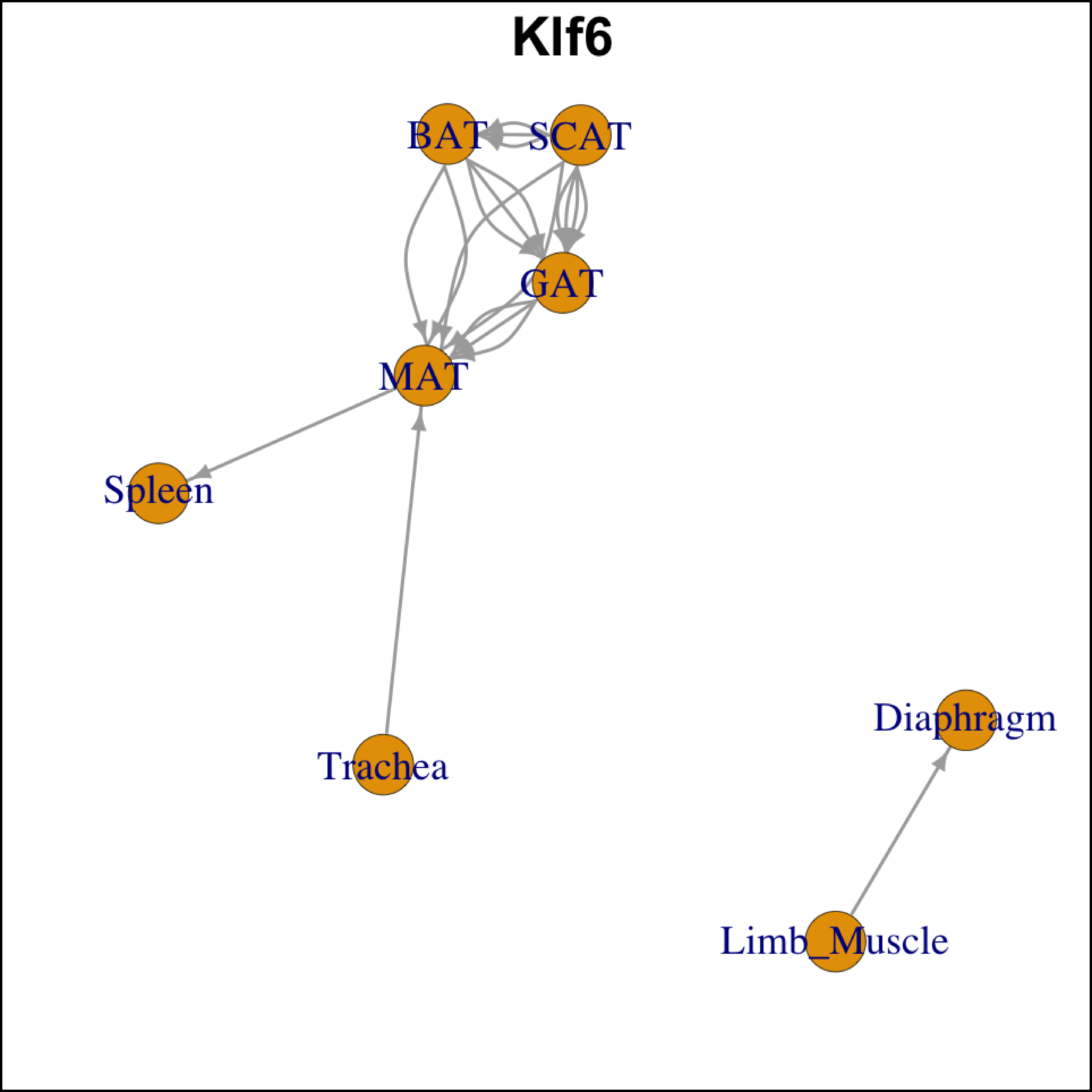
Klf6
BAT
SCAT
GAT
MAT
Spleen
Trachea
Diaphragm
Limb_Muscle

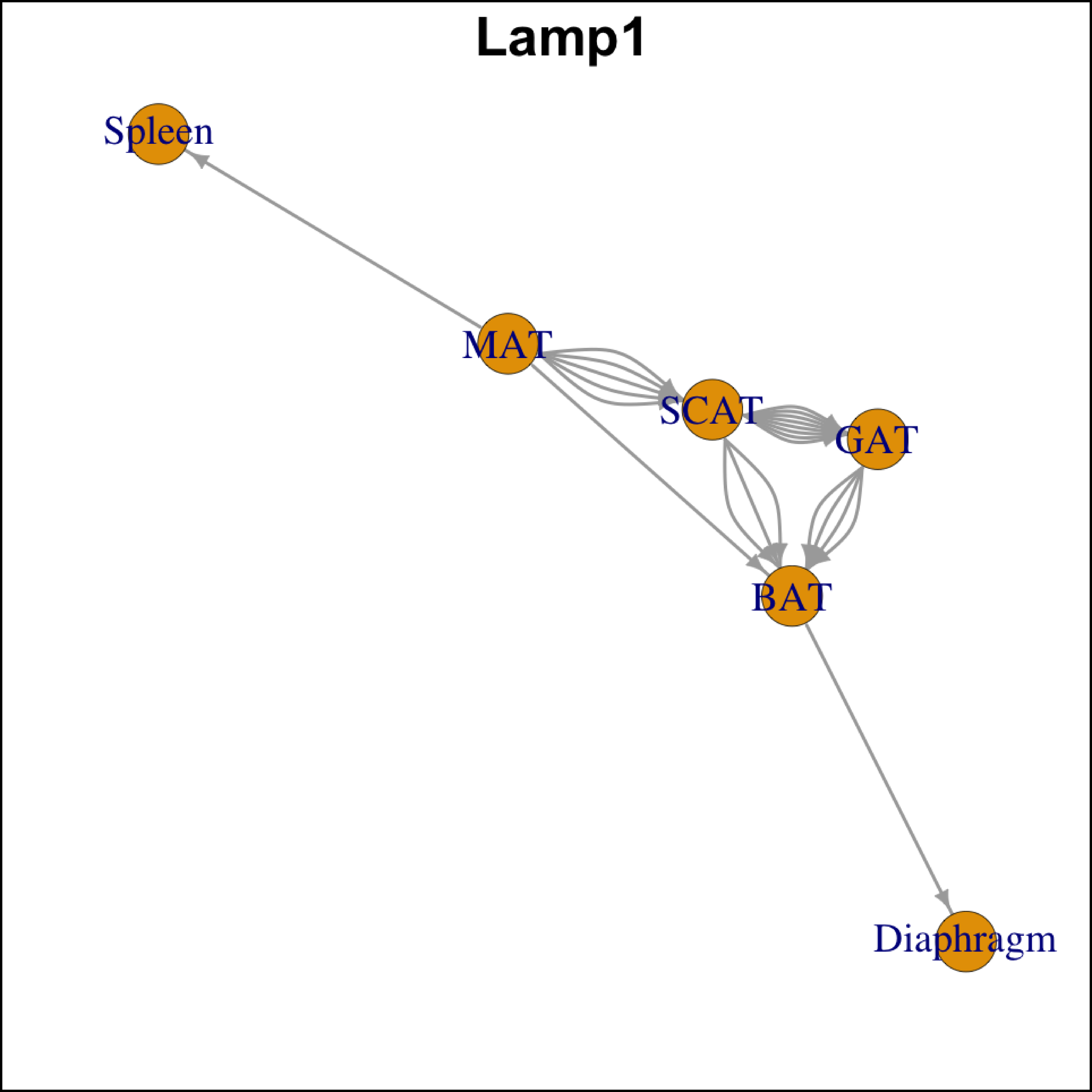
Lamp1
Spleen
MAT
SCAT
GAT
BAT
Diaphragm

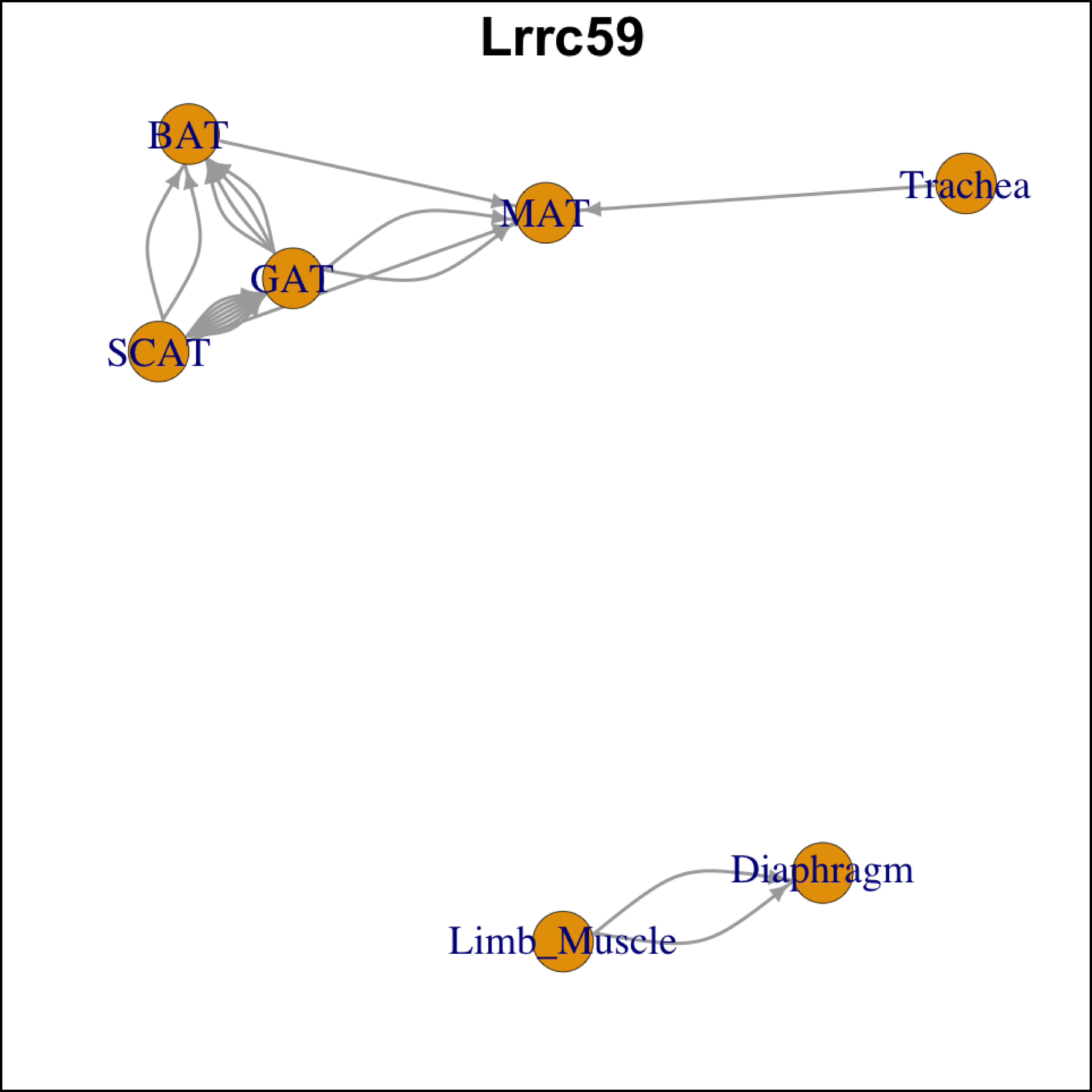
Lrrc59
BAT
MAT
Trachea
GAT
SCAT
Diaphragm
Limb_Muscle

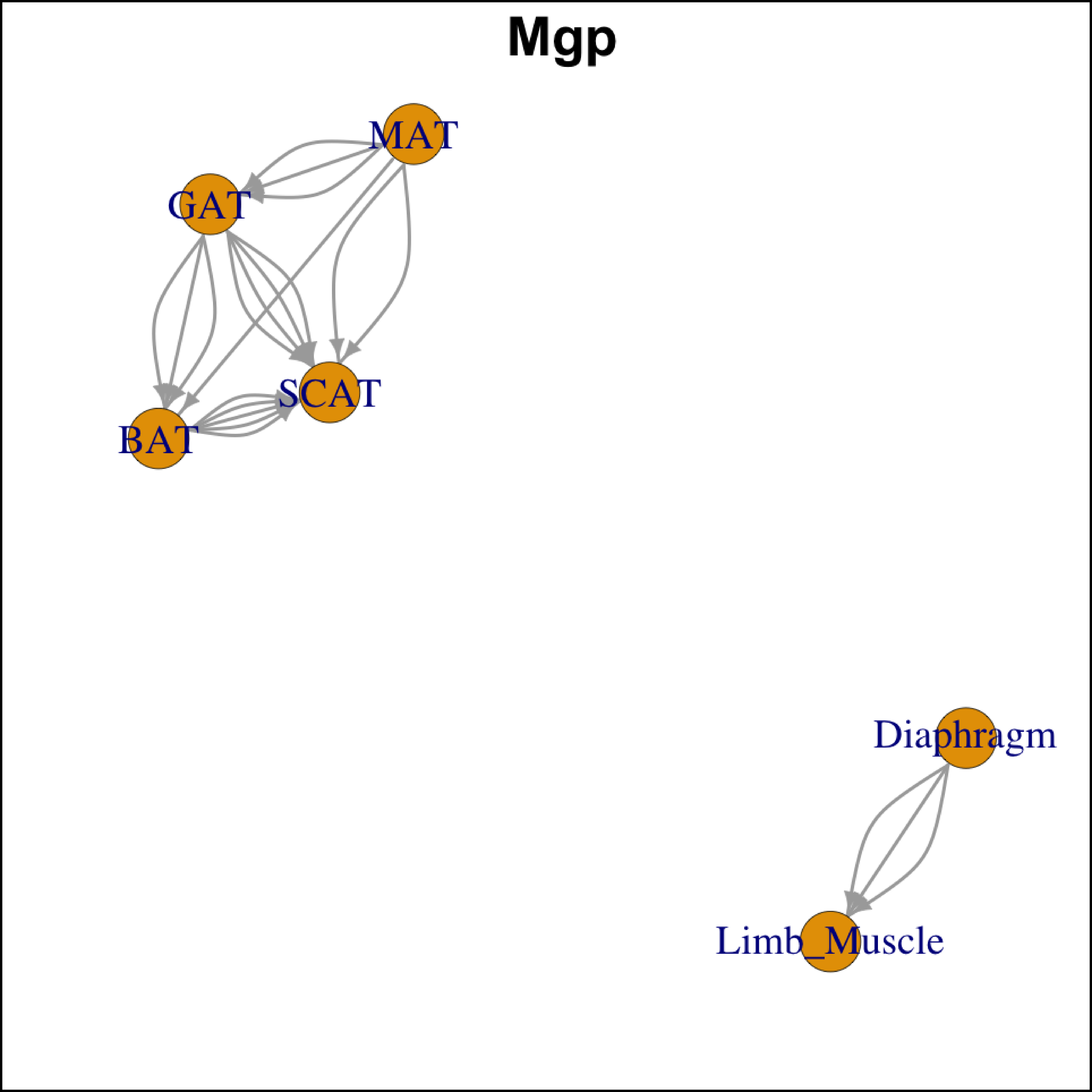

Mgp
MAT
GAT
SCAT
BAT
Diaphragm
Limb_Muscle

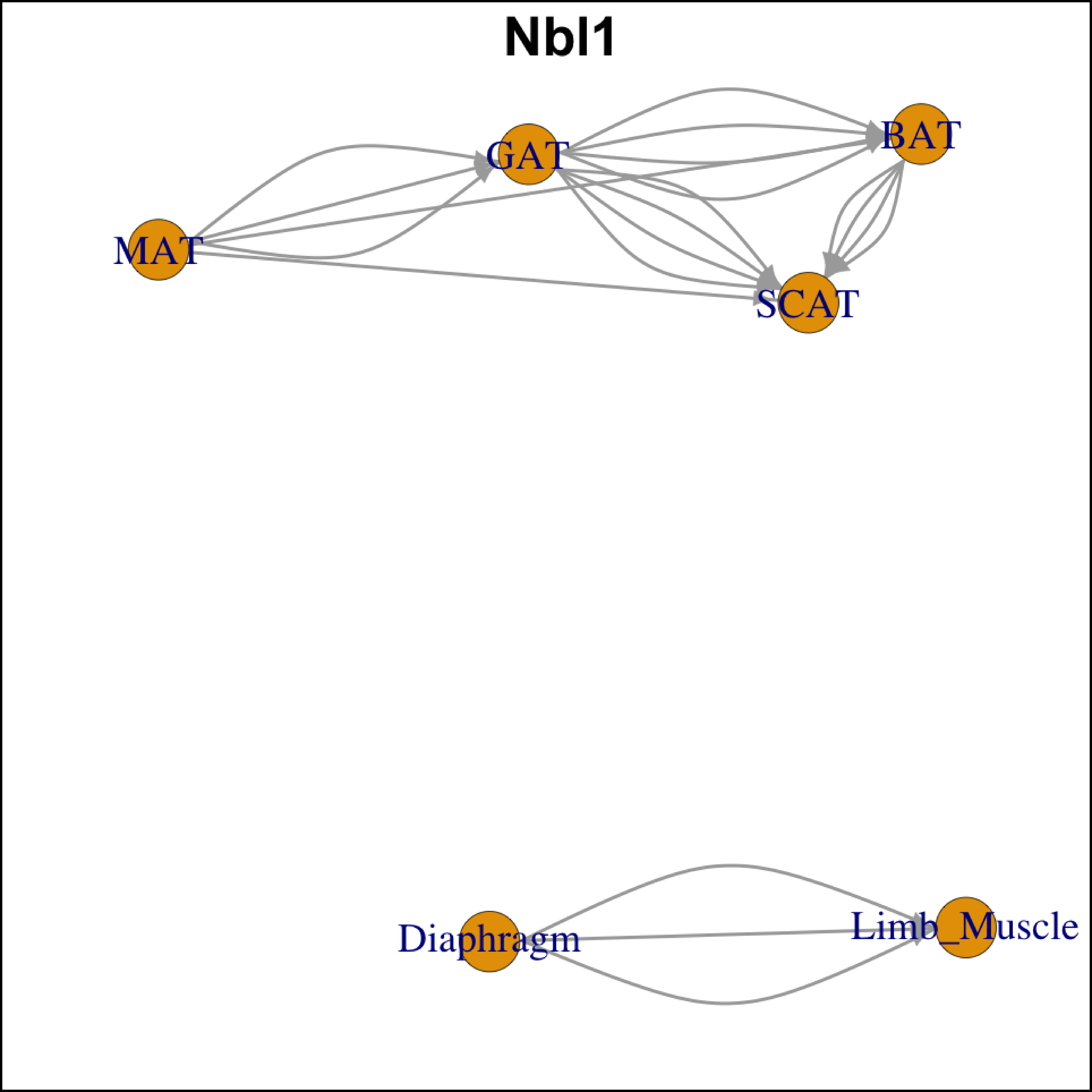
Nbl1
GAT
BAT
MAT
SCAT
Diaphragm
Limb_Muscle

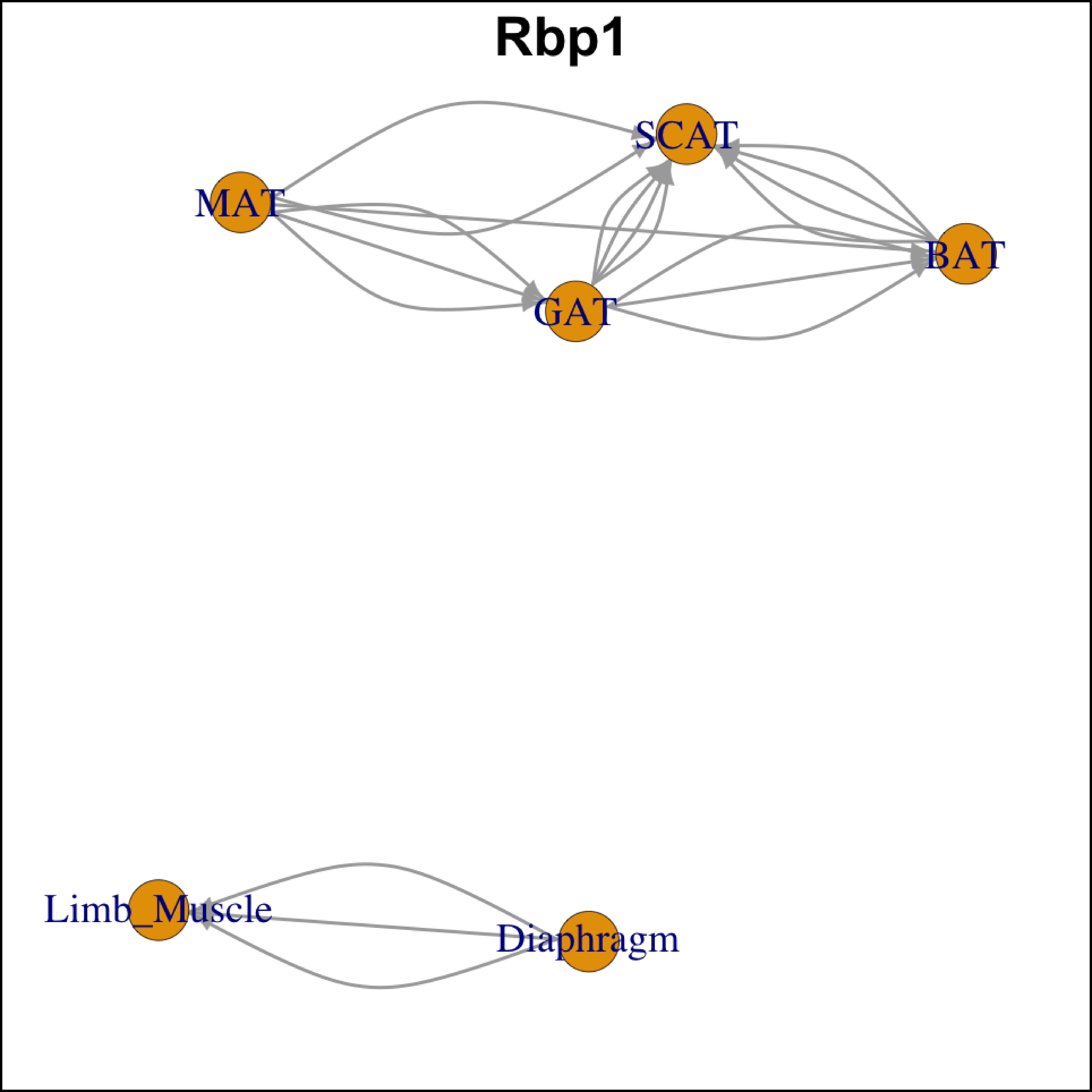
Rbp1
SCAT
MAT
BAT
GAT
Limb_Muscle
Diaphragm

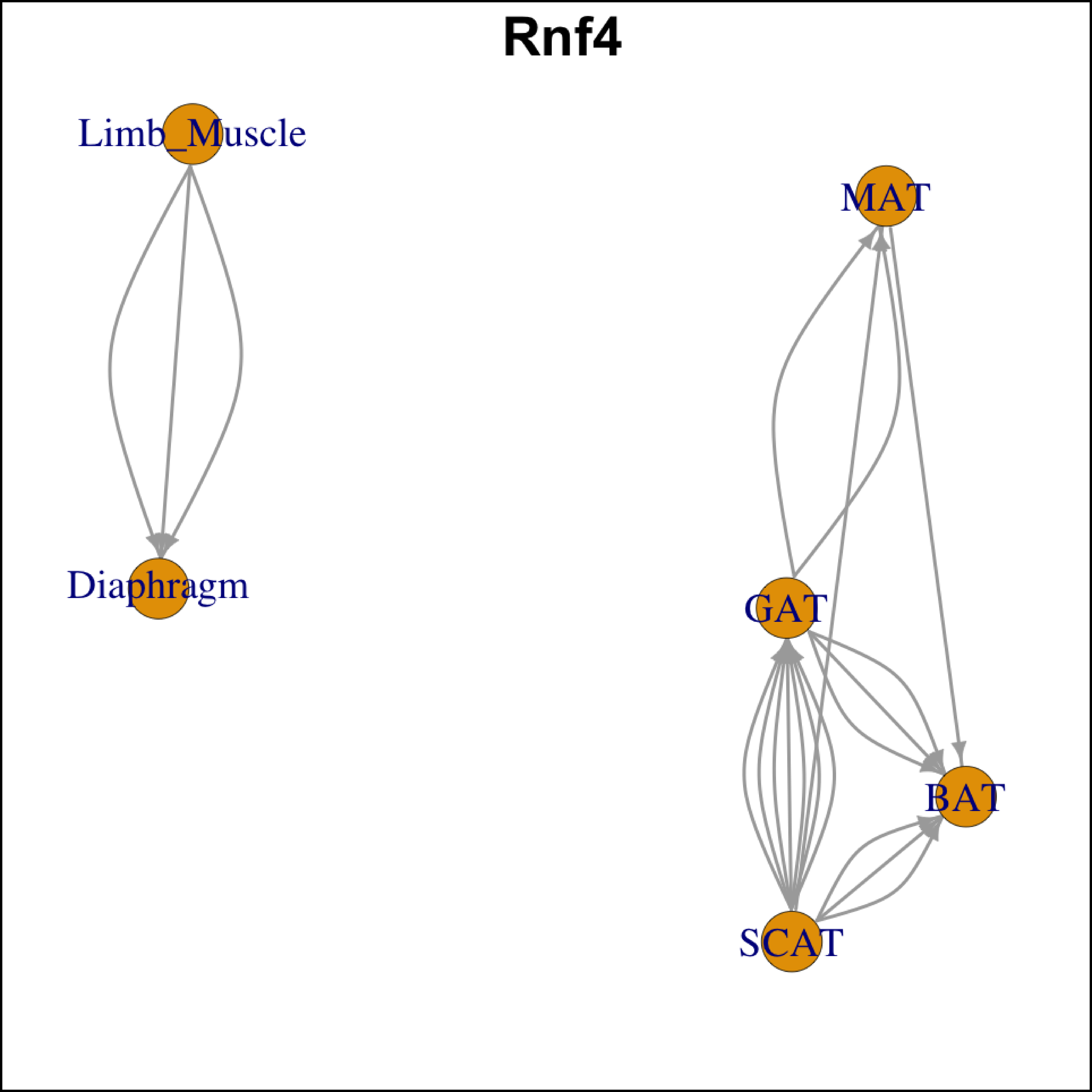
Rnf4
Limb_Muscle
Diaphragm
MAT
GAT
BAT
SCAT

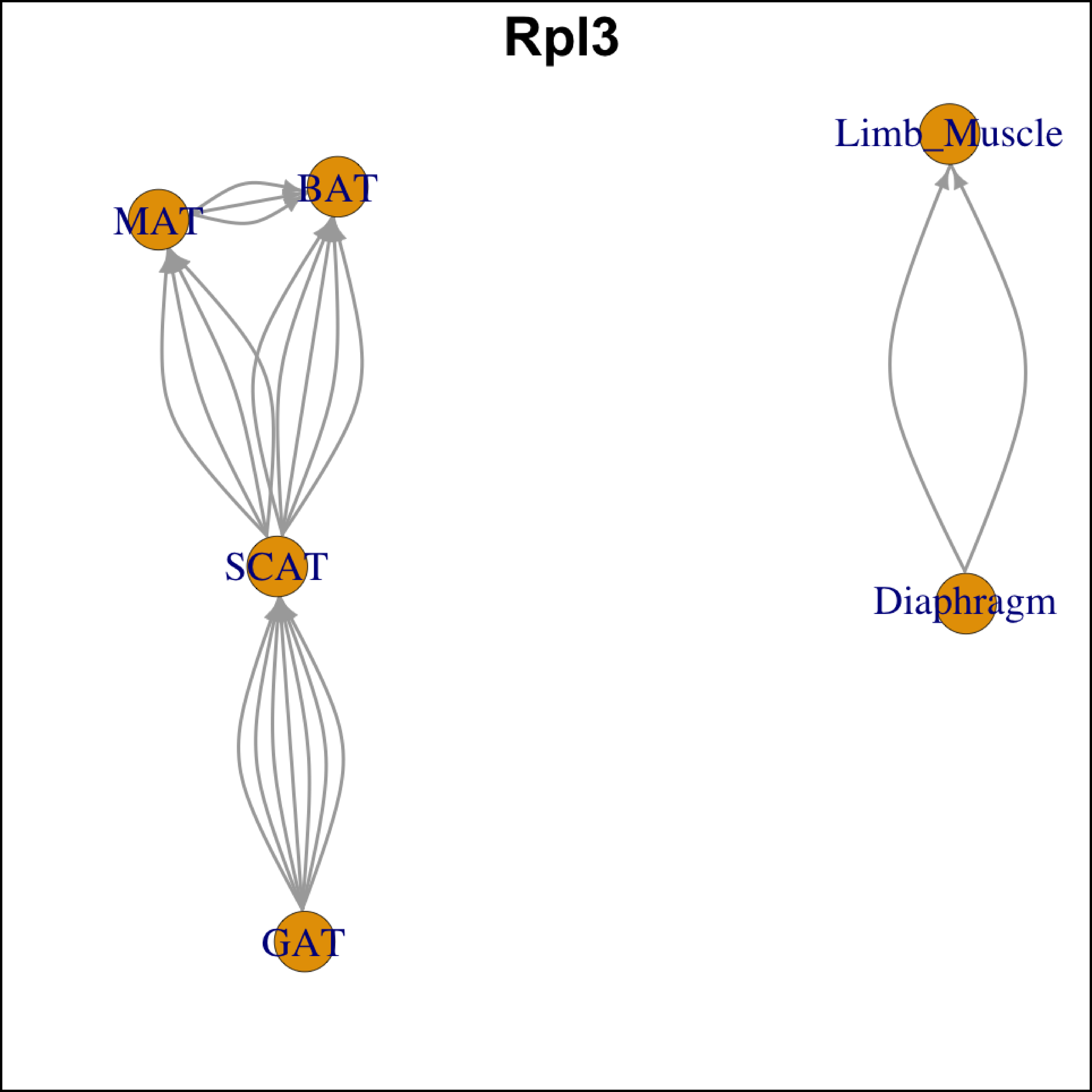
Rpl3
Limb_Muscle
BAT
MAT
SCAT
Diaphragm
GAT

Rpl5

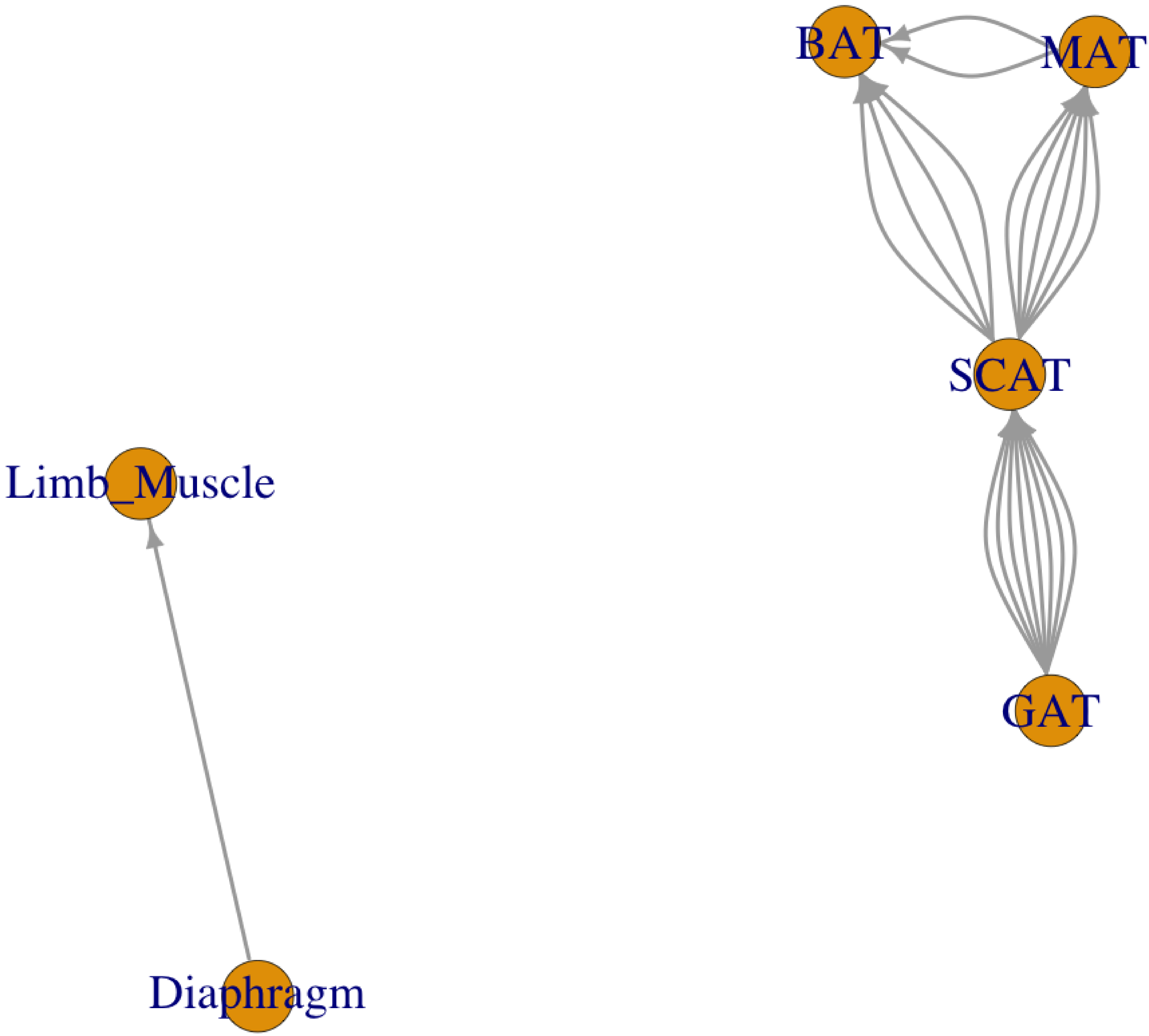

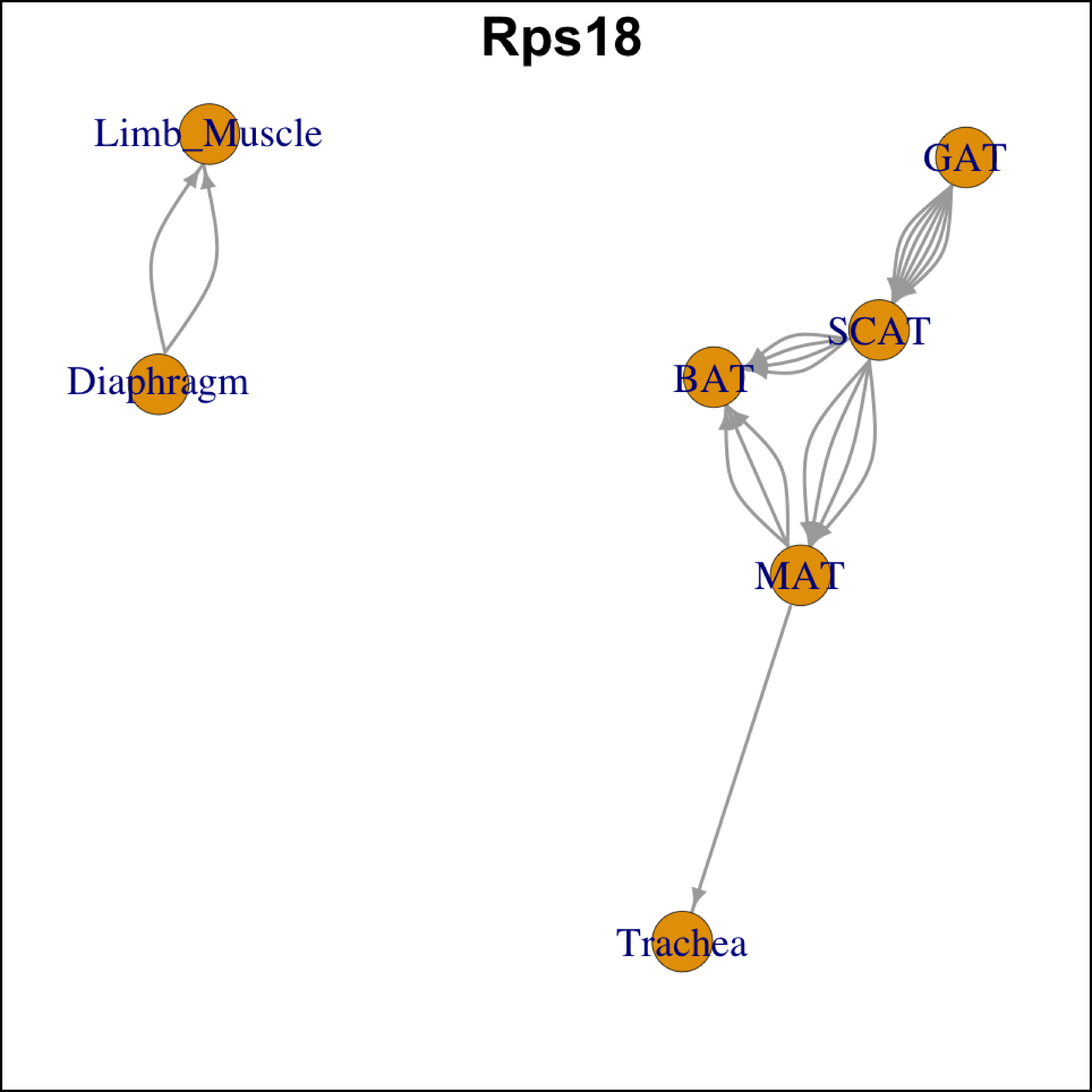
Rps18
Limb_Muscle
Diaphragm
GAT
SCAT
BAT
MAT
Trachea

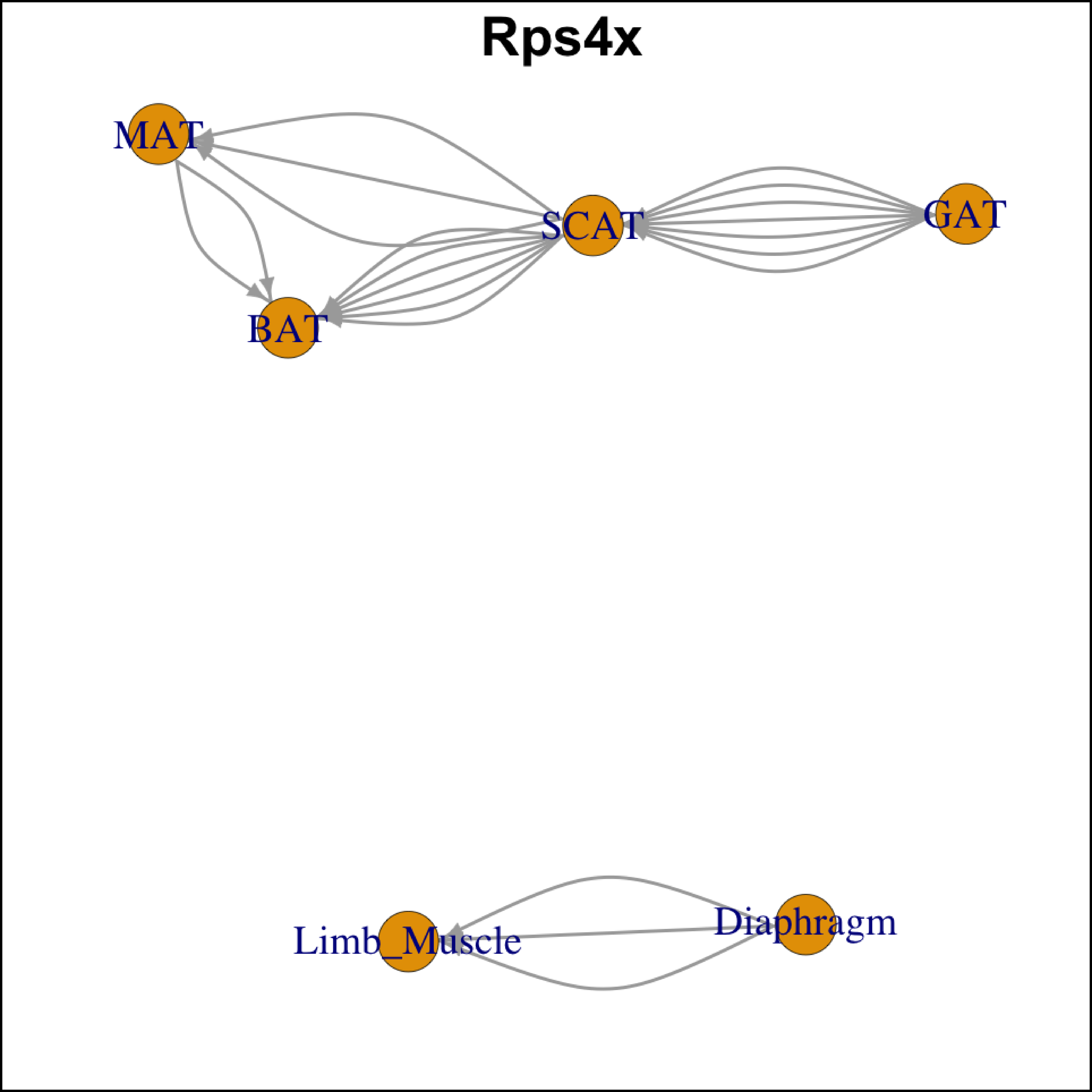
Rps4x
MAT
SCAT
GAT
BAT
Limb_Muscle
Diaphragm

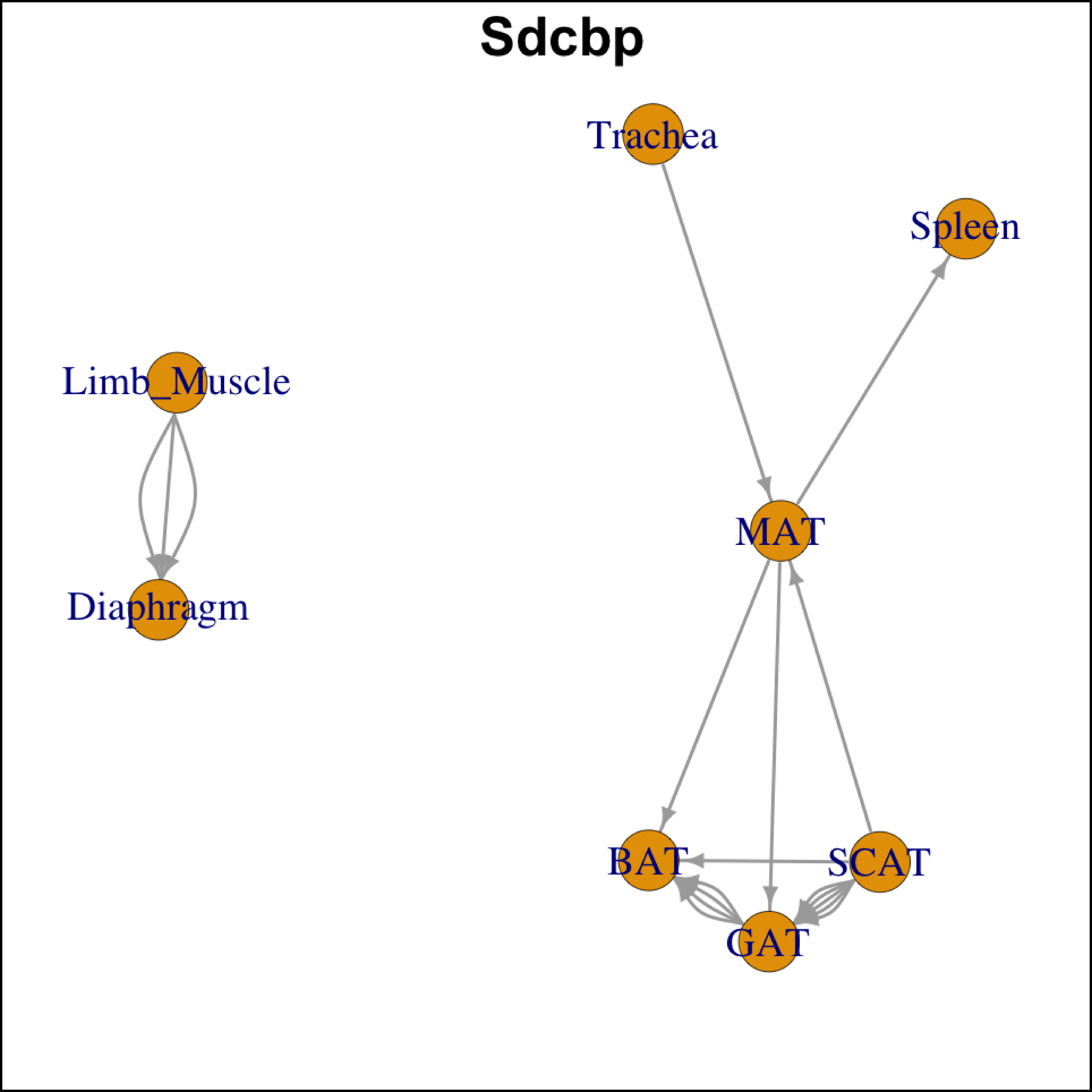
Sdcbp
Trachea
Spleen
Limb_Muscle
MAT
Diaphragm
BAT
SCAT
GAT

Sipa1

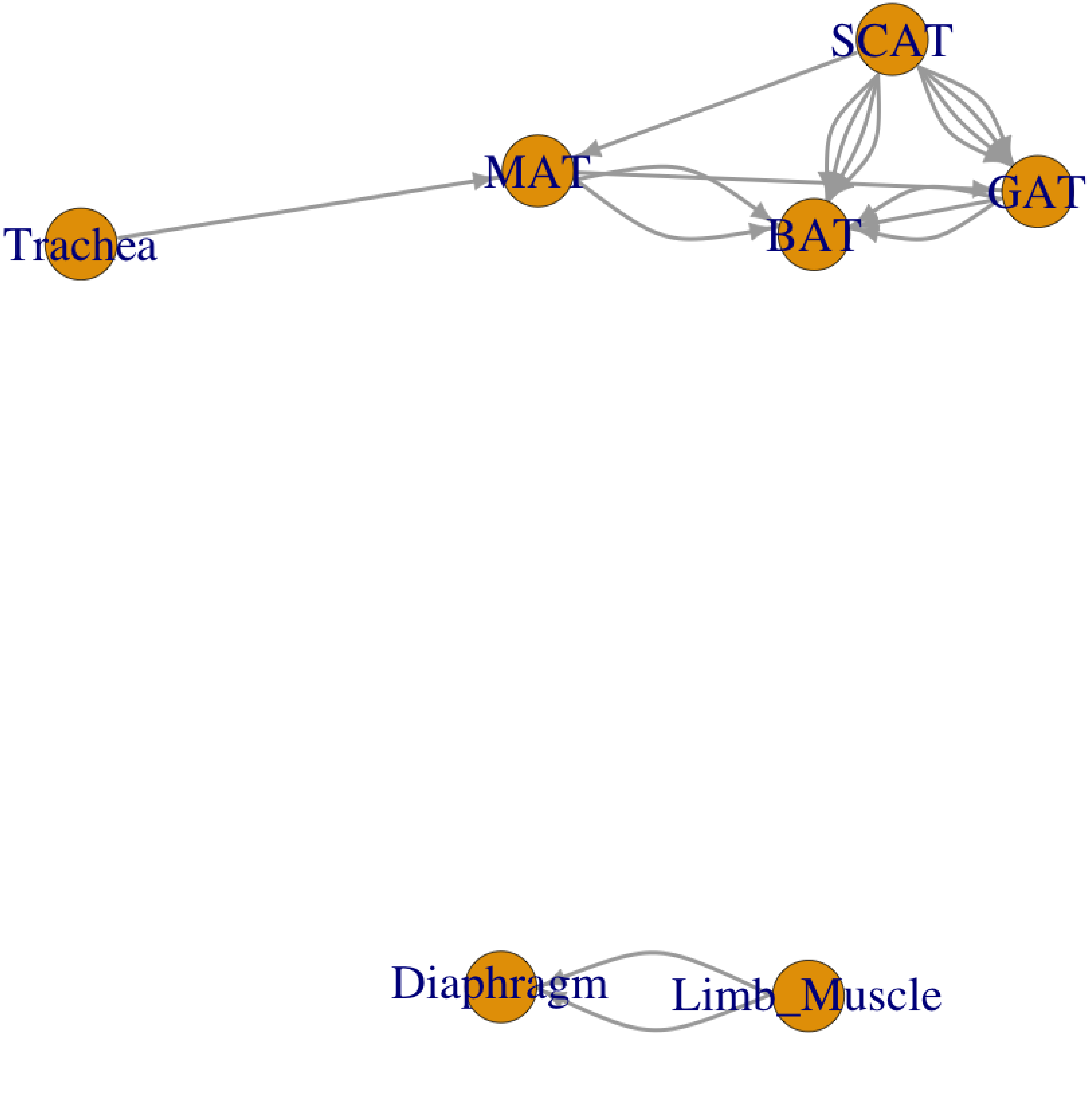

Slc39a14

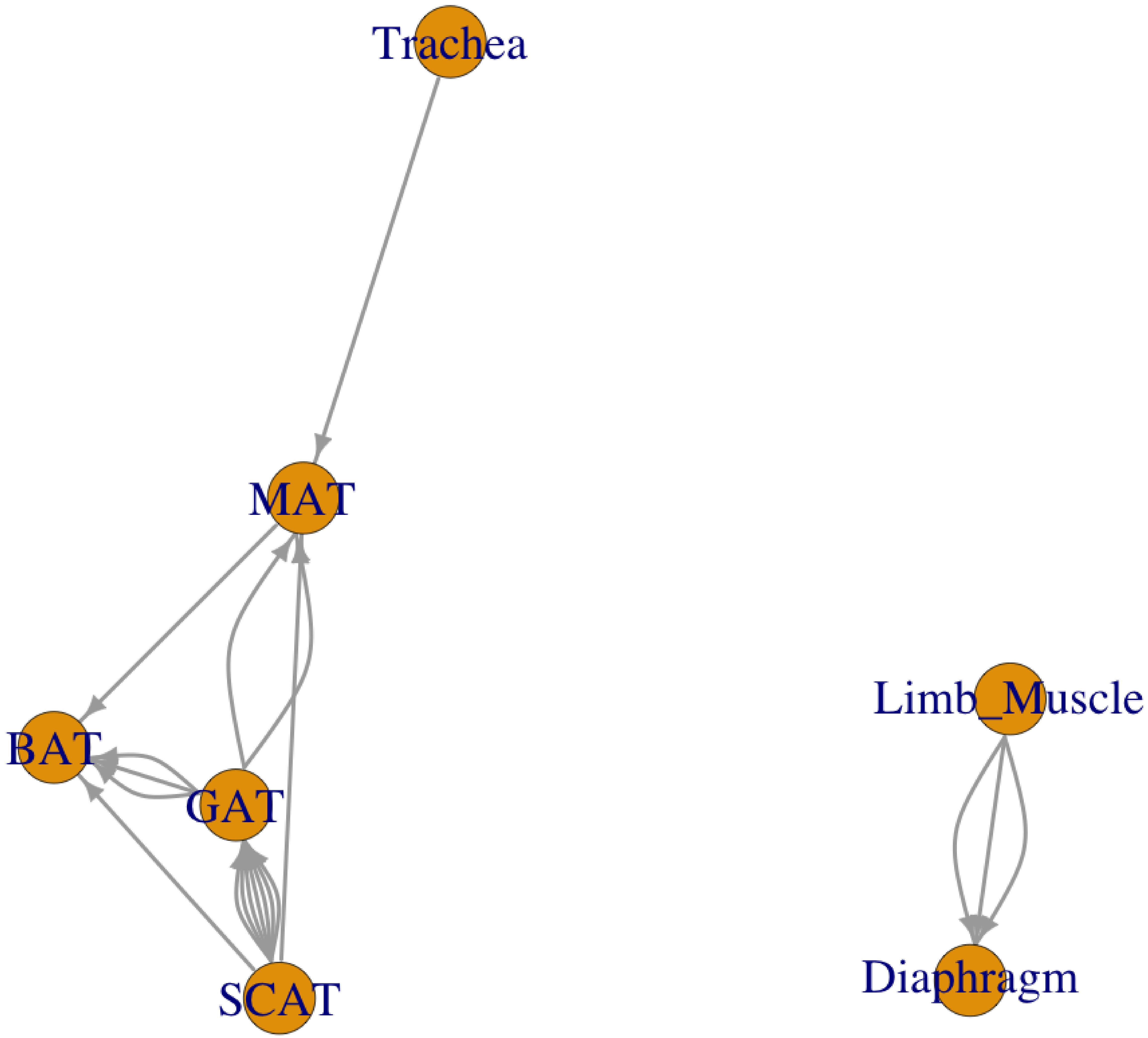

Stt3a

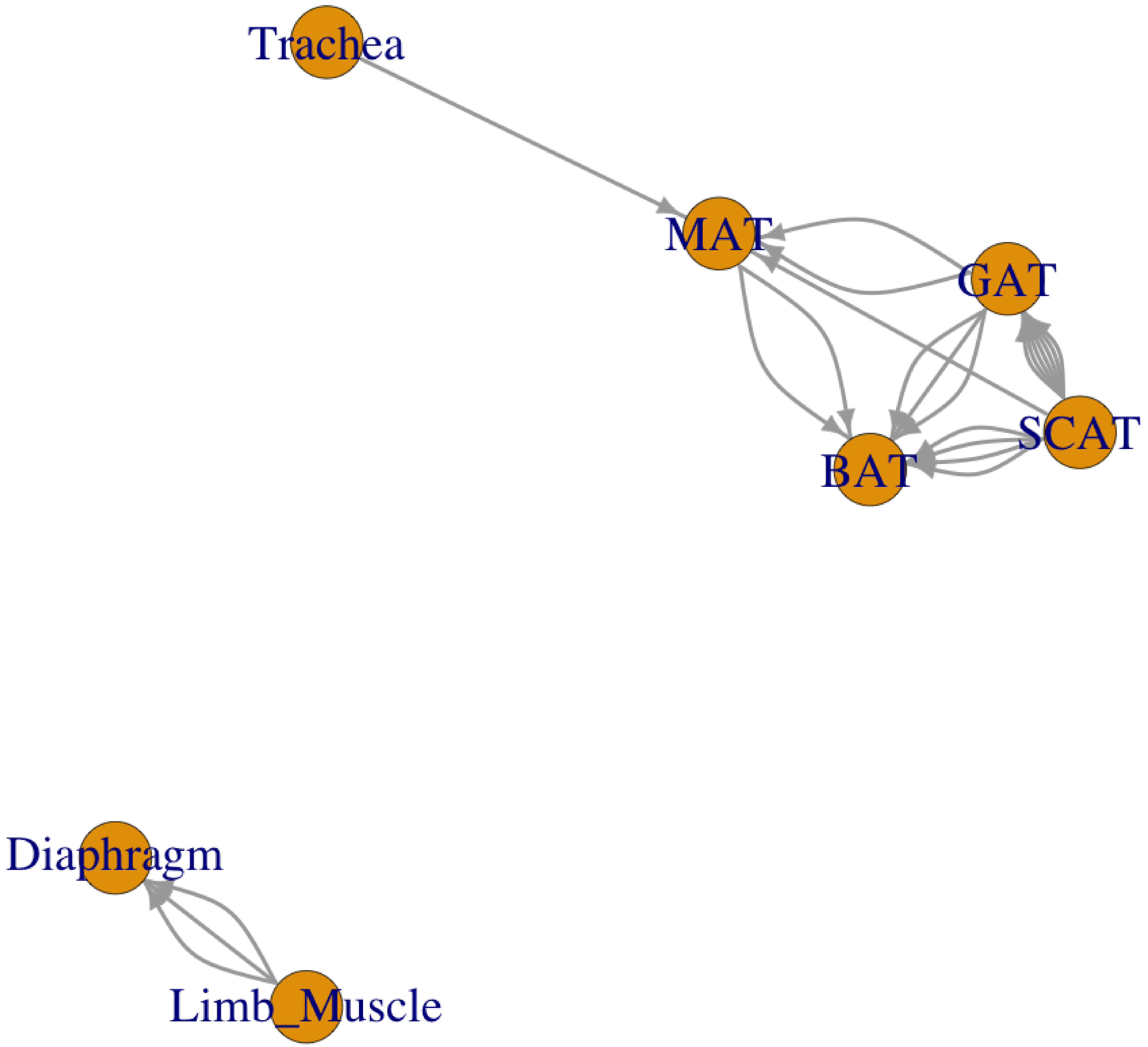

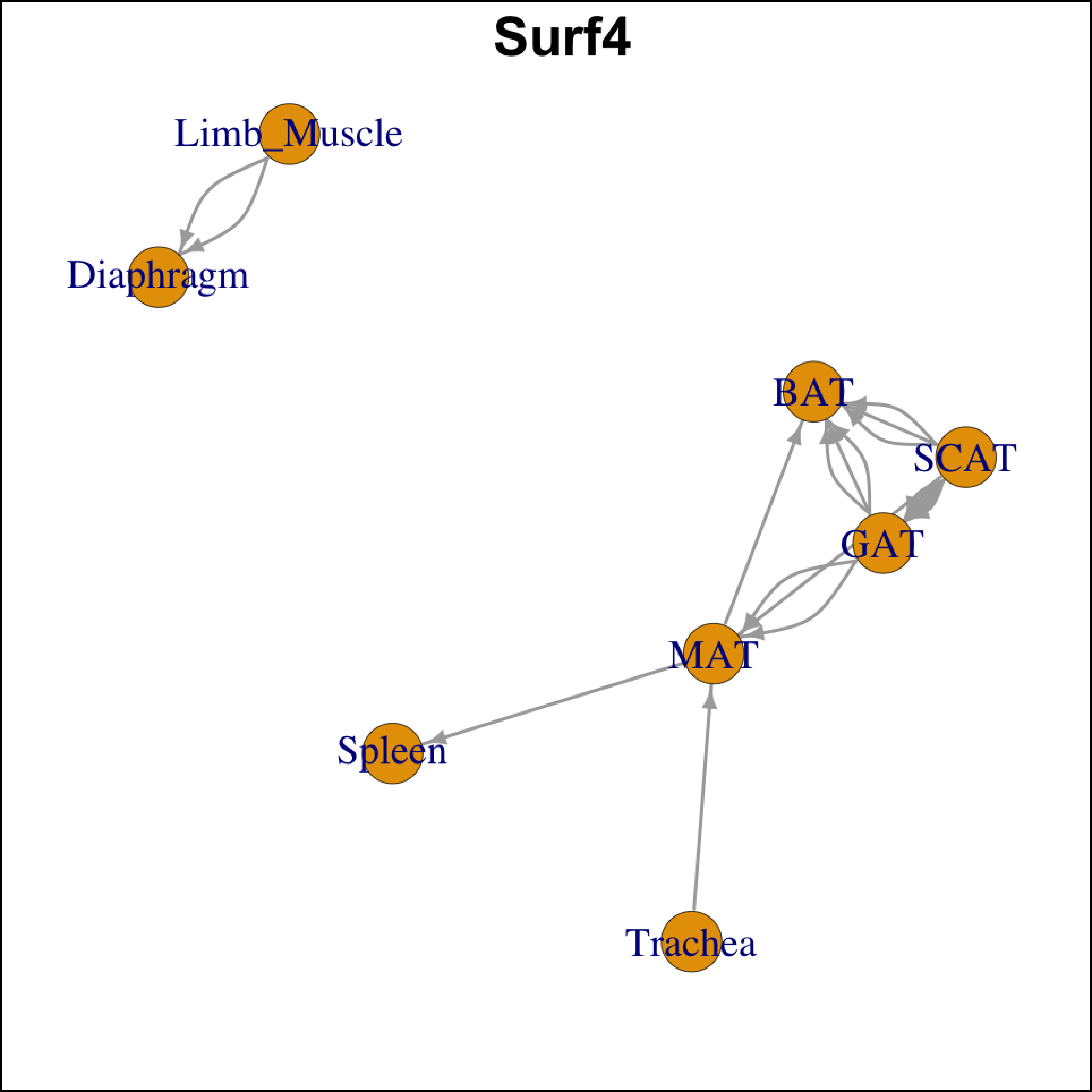
Surf4
Limb_Muscle
Diaphragm
BAT
SCAT
GAT
MAT
Spleen
Trachea

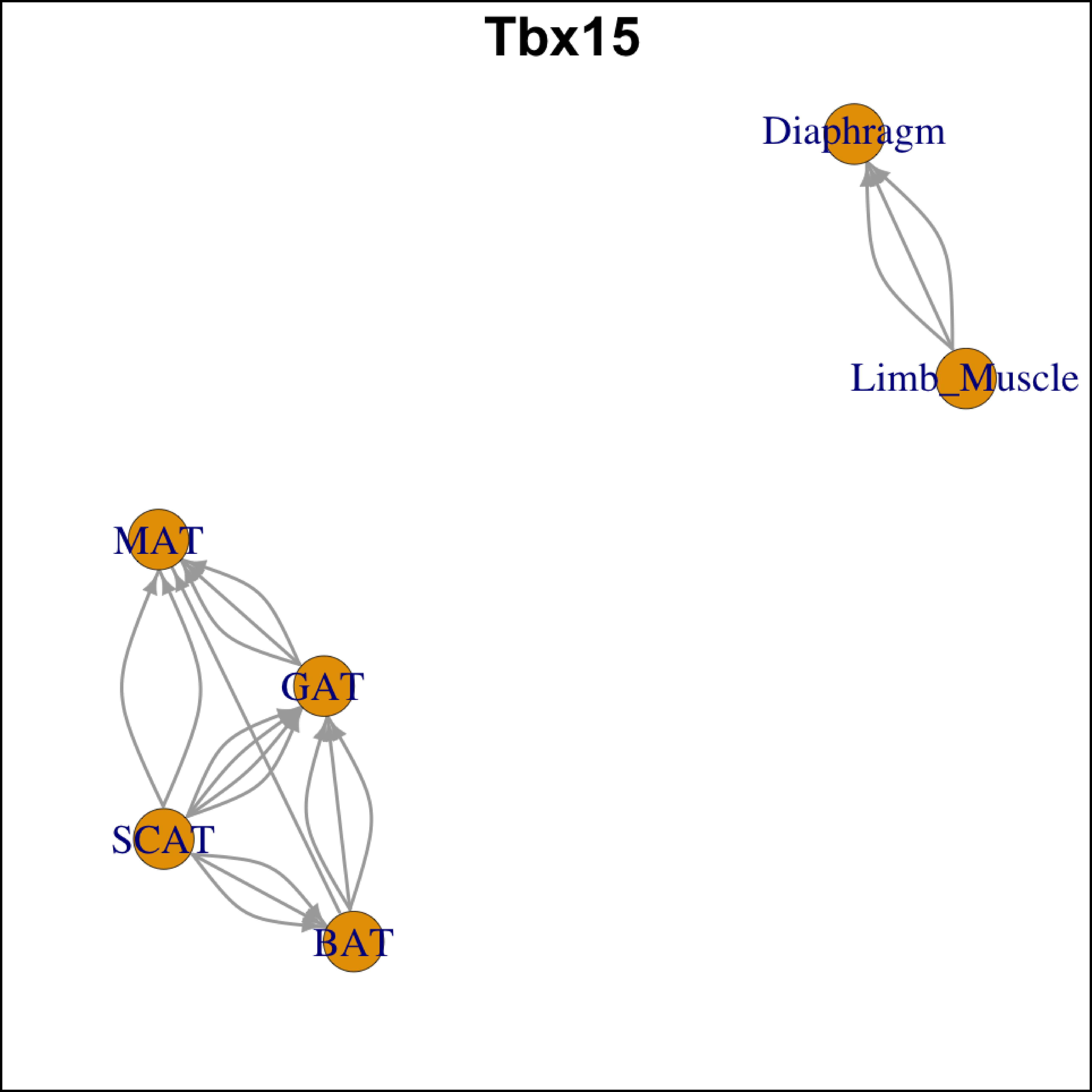
Tbx15
Diaphragm
Limb_Muscle
MAT
GAT
SCAT
BAT

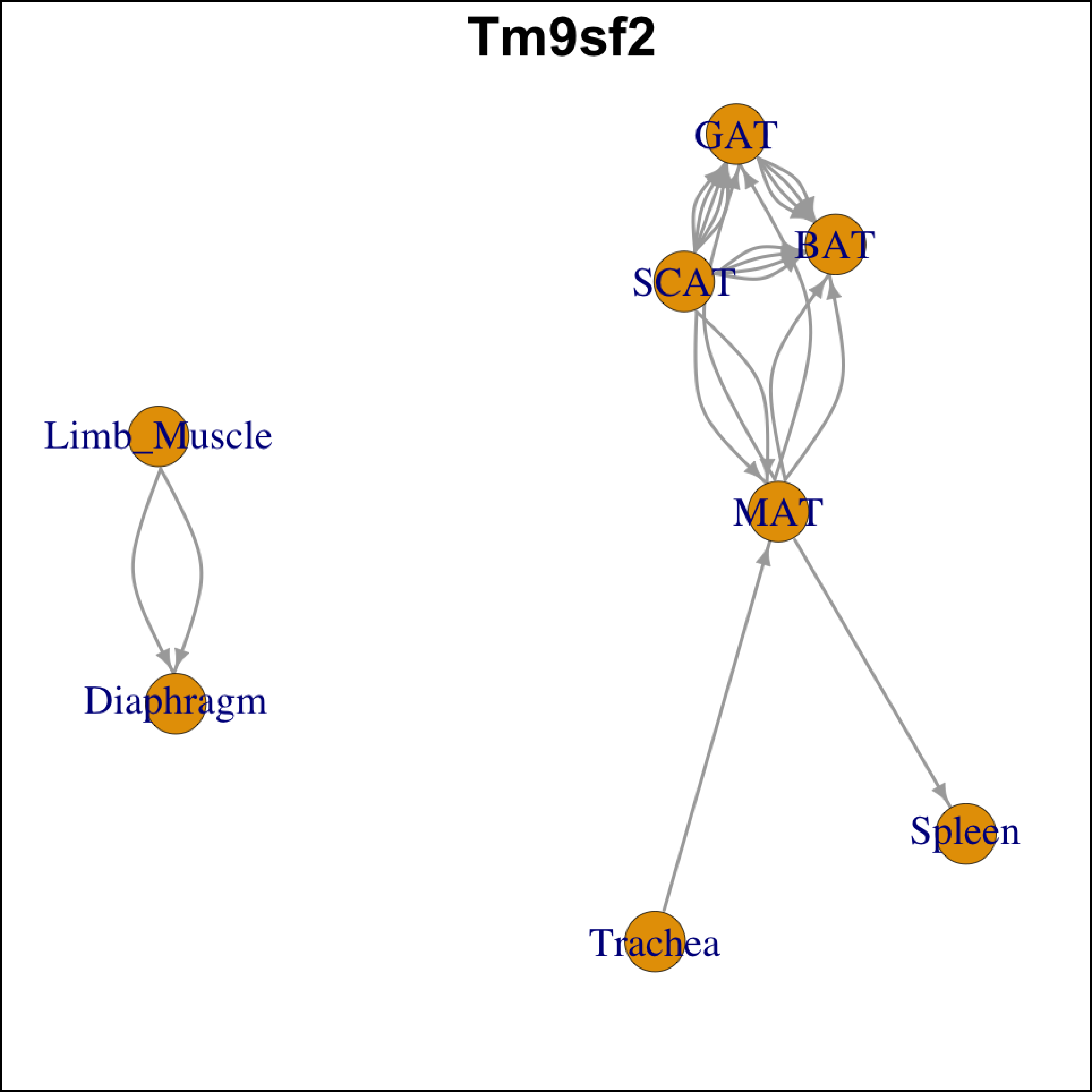
Tm9sf2
GAT
BAT
SCAT
Limb_Muscle
MAT
Diaphragm
Spleen
Trachea

Tmbim6

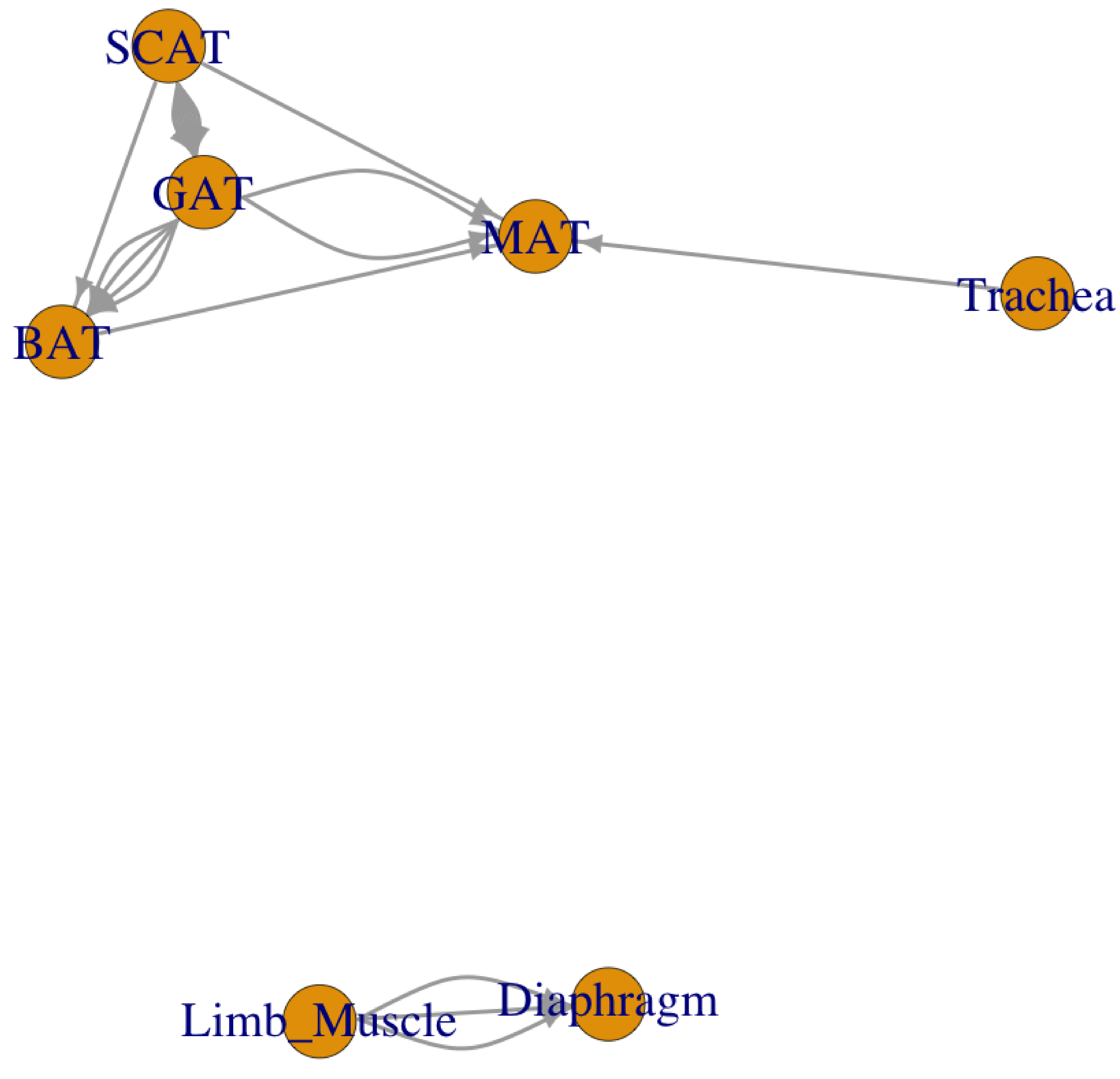

Uap1

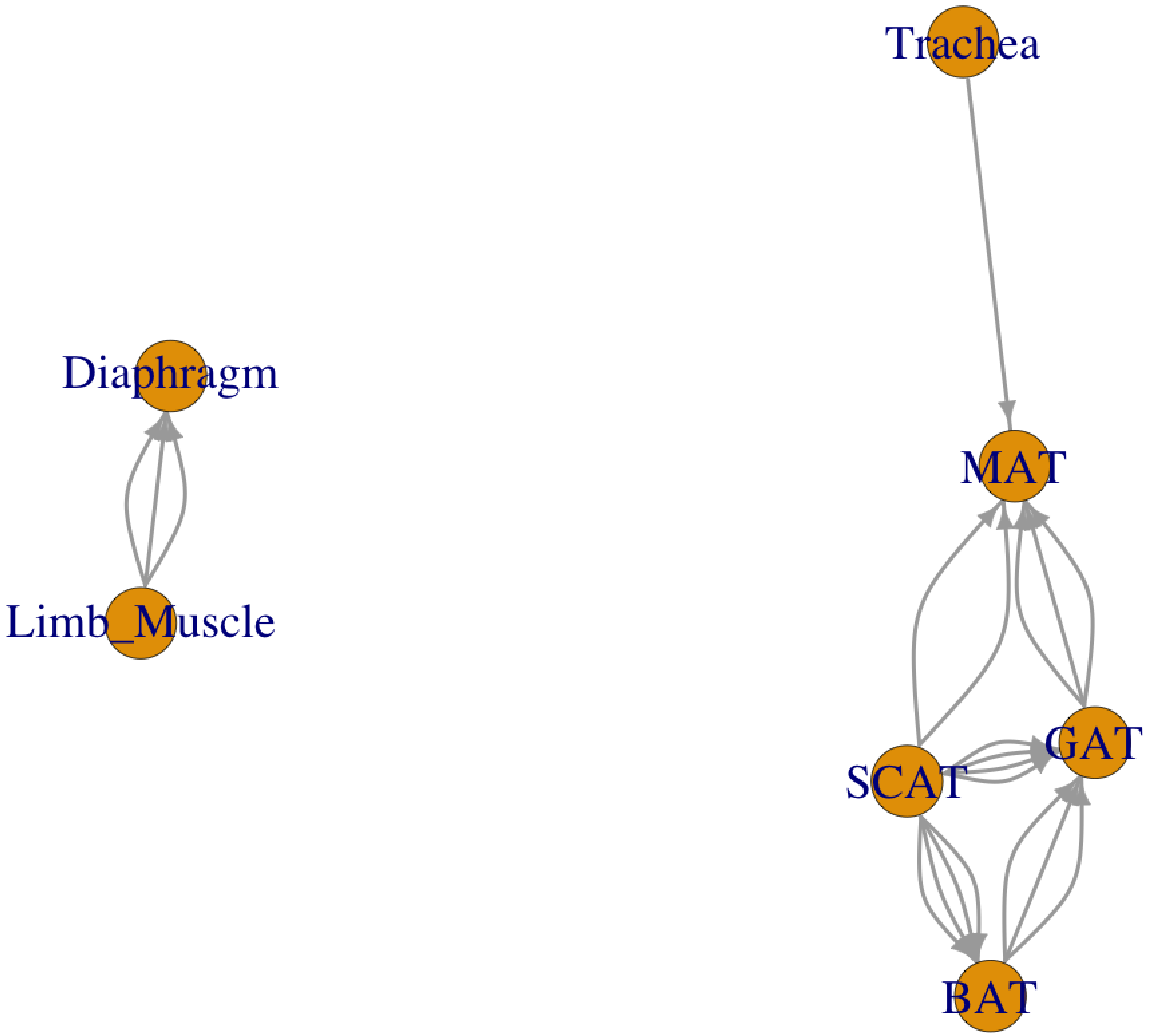

Ubc

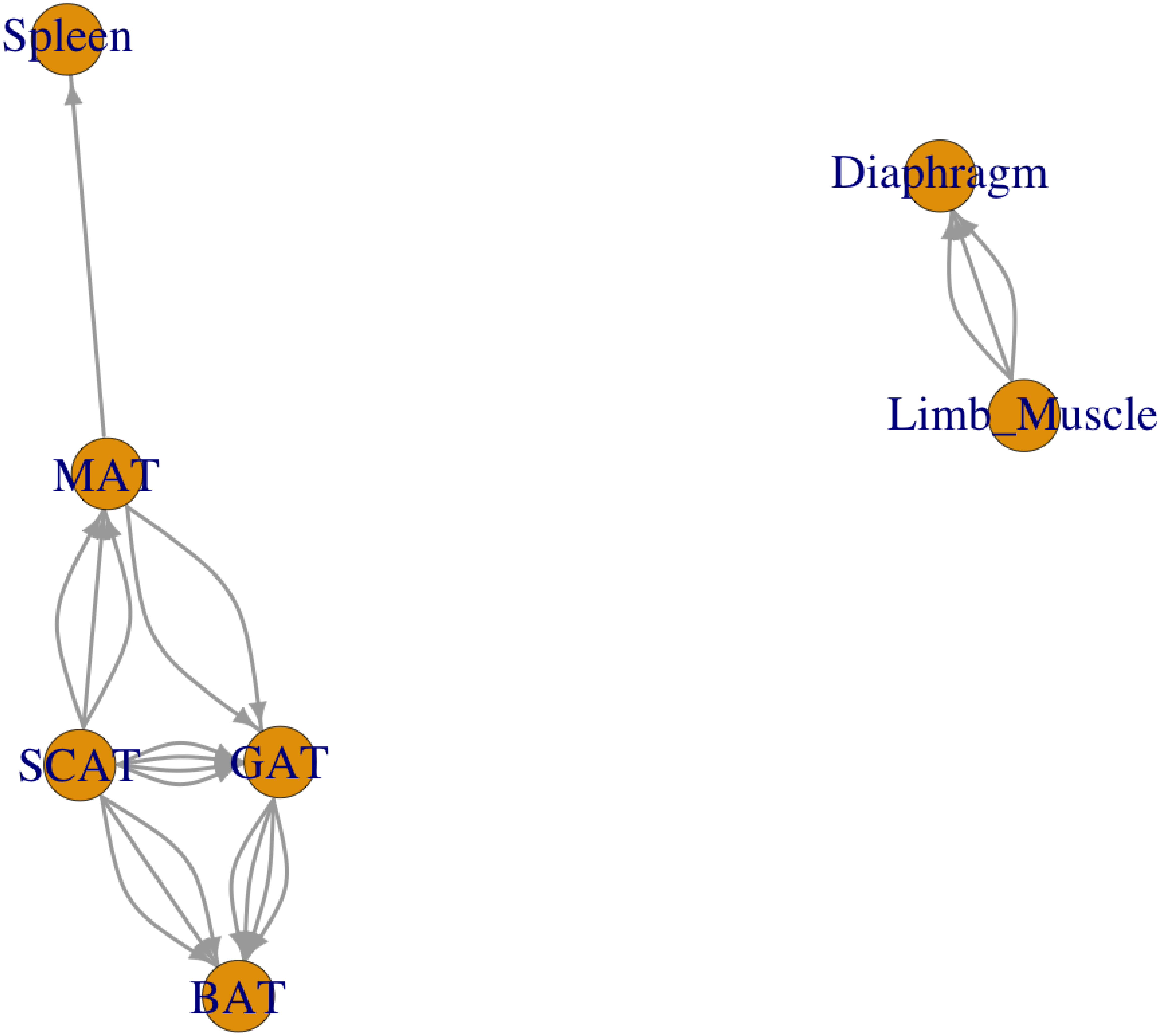

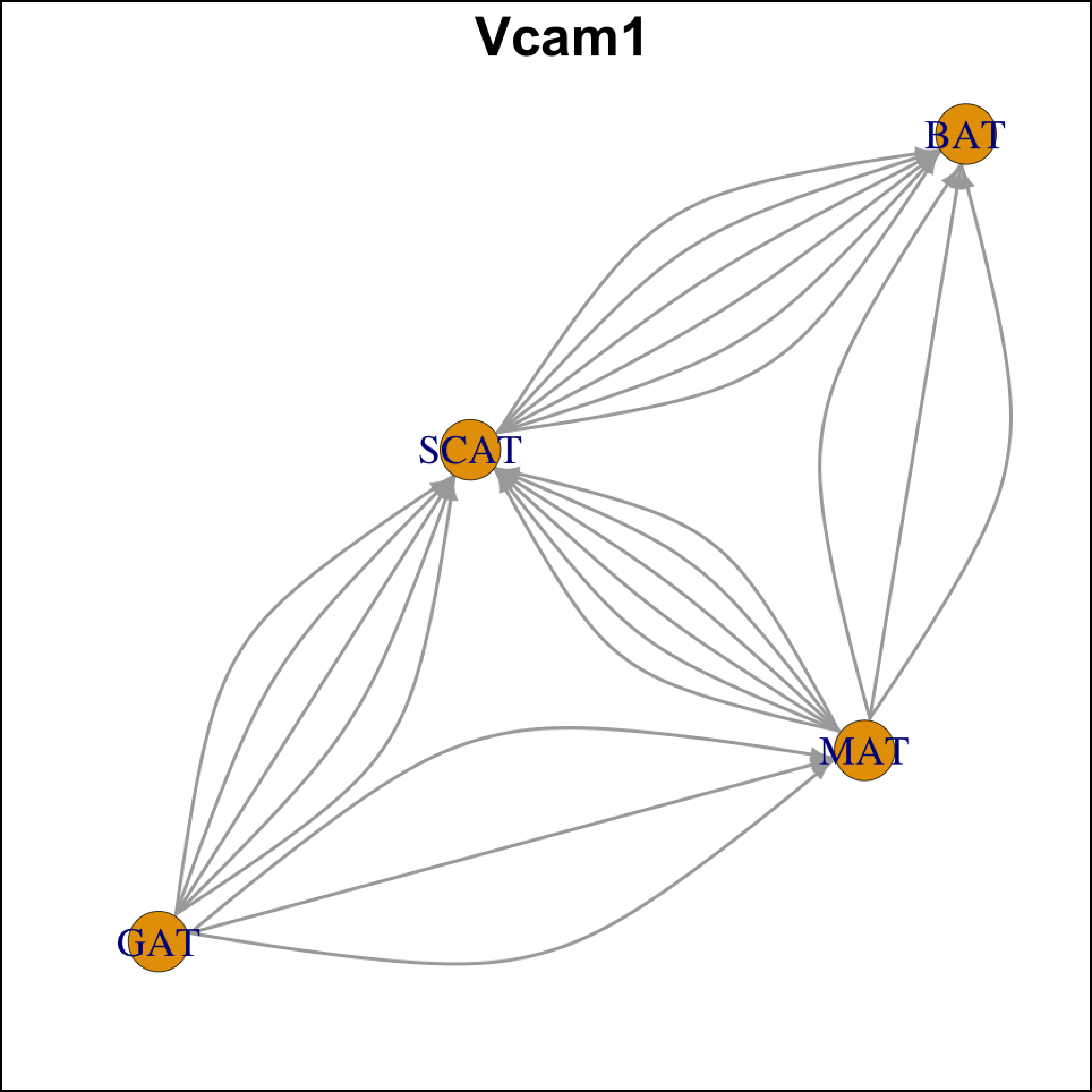
Vcam1
BAT
SCAT
MAT
GAT

Wdr1

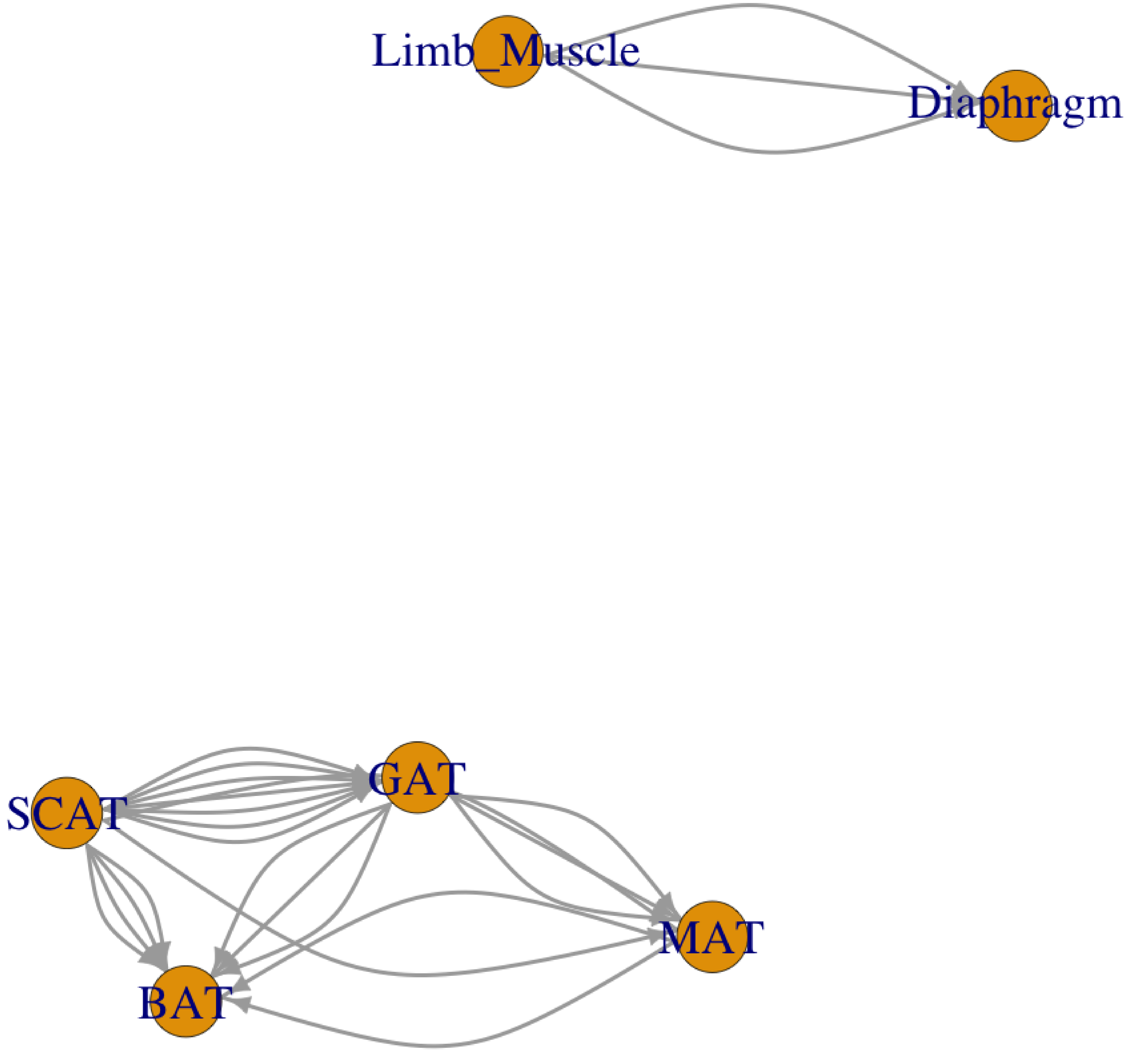

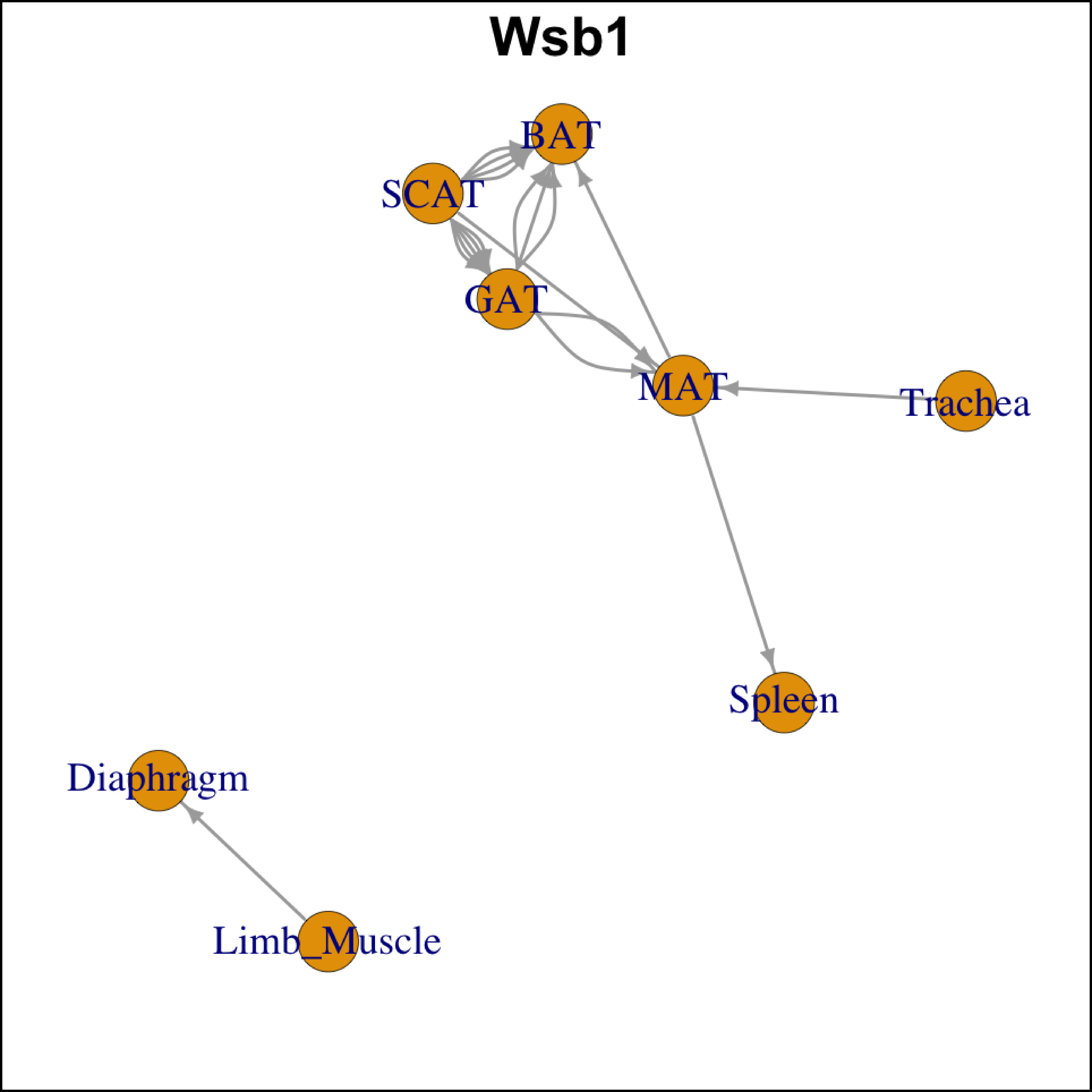
Wsb1
BAT
SCAT
GAT
MAT
Trachea
Spleen
Diaphragm
Limb_Muscle

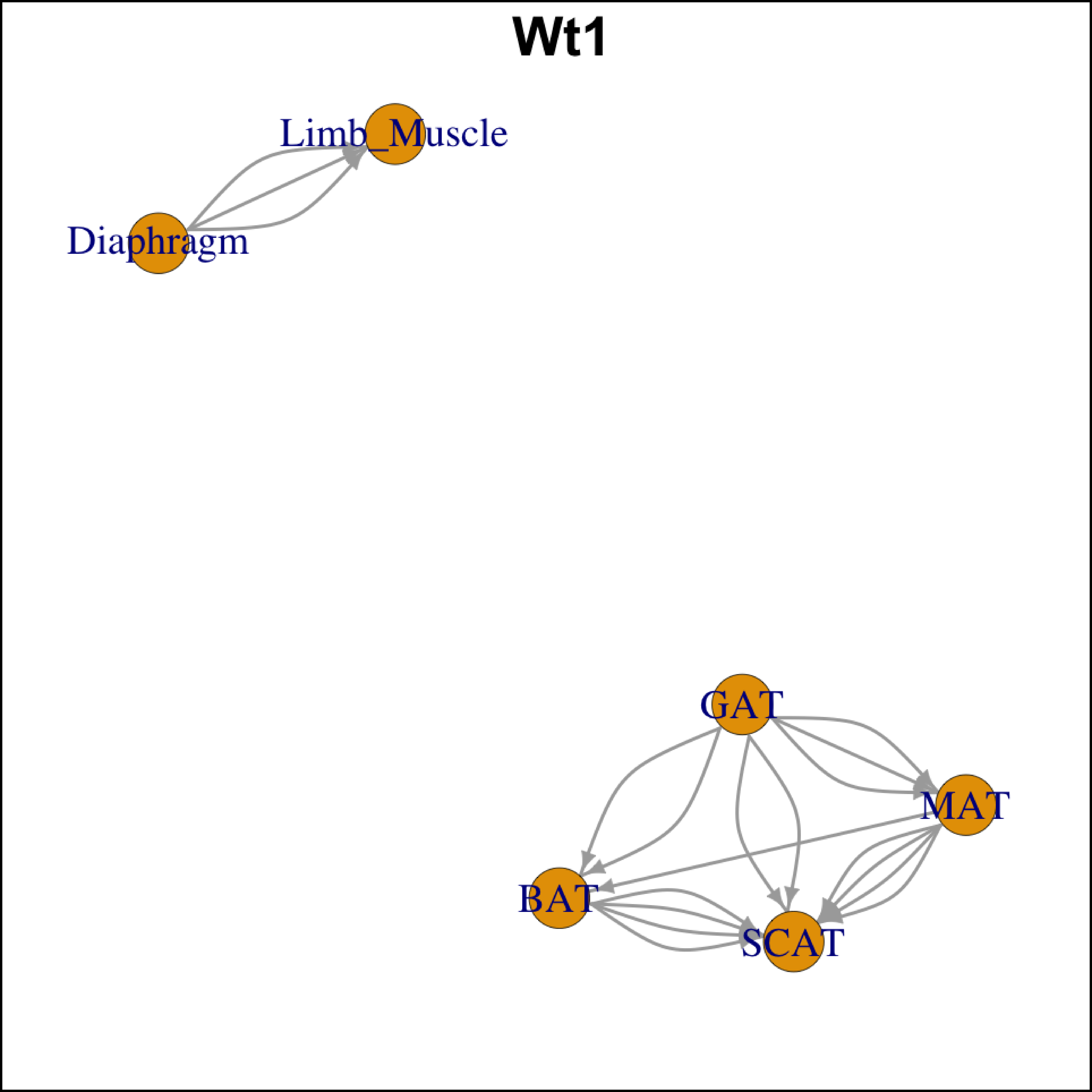
Wt1
Limb_Muscle
Diaphragm
GAT
MAT
BAT
SCAT
